# A Conceptual Review of Loneliness in Adults: Qualitative Evidence Synthesis

**DOI:** 10.3390/ijerph182111522

**Published:** 2021-11-02

**Authors:** Louise Mansfield, Christina Victor, Catherine Meads, Norma Daykin, Alan Tomlinson, Jack Lane, Karen Gray, Alex Golding

**Affiliations:** 1 Centre for Health and Wellbeing across the Lifecourse, College of Health, Medicine & Life Sciences, Brunel University London, Uxbridge UB8 3PH, UK; christina.victor@brunel.ac.uk (C.V.); kcrgray@gmail.com (K.G.); alexgolding73@gmail.com (A.G.); 2 Faculty of Health, Education, Medicine and Social Care, Anglia Ruskin University, Cambridge CB1 1PT, UK; catherine.meads@aru.ac.uk; 3 New Social Research, Faculty of IT and Communication Sciences, Tampere University, 33100 Tampere, Finland; norma.daykin@tuni.fi; 4 Centre for Arts and Wellbeing, School of Humanities, University of Brighton, Brighton BN2 4AT, UK; a.tomlinson@brighton.ac.uk (A.T.); jack.lane@sportnz.org.nz (J.L.)

**Keywords:** loneliness, conceptual review, social loneliness, emotional loneliness, existential loneliness

## Abstract

The paper reports an evidence synthesis of how loneliness is conceptualised in qualitative studies in adults. Using PRISMA guidelines, our review evaluated exposure to or experiences of loneliness by adults (aged 16+) in any setting as outcomes, processes, or both. Our initial review included any qualitative or mixed-methods study, published or unpublished, in English, from 1945 to 2018, if it employed an identified theory or concept for understanding loneliness. The review was updated to include publications up to November 2020. We used a PEEST (Participants, Exposure, Evaluation, Study Design, Theory) inclusion criteria. Data extraction and quality assessment (CASP) were completed and cross-checked by a second reviewer. The Evidence of Reviews of Qualitative Research (CERQual) was used to evaluate confidence in the findings. We undertook a thematic synthesis using inductive methods for peer-reviewed papers. The evidence identified three types of distinct but overlapping conceptualisations of loneliness: social, emotional, and existential. We have high confidence in the evidence conceptualising social loneliness and moderate confidence in the evidence on emotional and existential loneliness. Our findings provide a more nuanced understanding of these diverse conceptualisations to inform more effective decision-making and intervention development to address the negative wellbeing impacts of loneliness.

## 1. Introduction

Most of us will encounter loneliness at some point in our lives. Indeed, some philosophers argue that loneliness is a universal human experience [1,2]. This experience may be momentary or protracted, occur frequently or rarely, and vary in intensity. Loneliness is characterised as a homogeneous, static, and/or linear experience that is quantitatively accessible (i.e., we can measure it), and it is understood as a problem about which ‘something’ can and should be done to prevent or cure it [3]. In public health, for example, loneliness has been problematised and medicalised because of the associations with a range of negative mental and physical health outcomes [4], including increased mortality, morbidity, poorer health behaviours and excess service use [5], cardiovascular disease [6,7], reduced physical activity [8,9], poorer cognitive function [10,11] and depression [12,13]. Distinct but related concepts, most notably loneliness and social isolation, but also living alone, aloneness, and solitude are often conflated despite not being linguistically, empirically, or conceptually interchangeable. Despite the almost universality of the experience of loneliness, and an extensive research literature, it remains an enigmatic concept for individuals, researchers, policymakers, and practitioners since it is the outcome of an individual’s subjective experience.

Understanding loneliness is made more challenging because it is characterised by differing antecedents across varying populations and across individual life courses [14]. There is a lack of clarity about theories of loneliness and their application in empirical studies, and how they should be evaluated, measured, and applied in policy and practice [4,15,16,17]. When interpreting and using evidence about loneliness, these conceptual challenges are important to identify and address because they will have a profound influence on the generation, interpretation, and potential impact of evidence on policy, practice, and research in the field. In addition, there is a growing body of qualitative research that has not been fully explored for its contribution to bringing conceptual clarity to loneliness research. We undertook a synthesis of qualitative studies to start to address this evidence gap in 2018 with the aim of identifying, synthesising, and reporting on how the included studies have conceptualised loneliness as a way of enriching thinking and informing decision-making and practice in the field. We were not developing a new concept of loneliness. Following guidelines that reviews should be updated two years after publication, we updated the previous review to include relevant papers published up to 2020. This paper combines both reviews and summarises all the relevant literature on conceptualisations of social, emotional, and existential loneliness in diverse populations, their positive and negative attributes, and the ways people have found to alleviate loneliness as reported in academic journal articles and grey literature.

## 2. Materials and Methods

### 2.1. Methods

The protocol was registered with the PROSPERO database (registration number: CRD42019124565). We followed PRISMA guidelines for conducting and reporting in this updated evidence review [18]. We employed a two-stage method, based on that proposed by key authors in the field [19]. Stage 1 identified evidence on conceptualisations, models, frameworks, and theories of loneliness and related concepts or domains. We made provision for a Stage 2 process to include additional evidence reviews for specific concepts and theories identified in Stage 1. This was not deemed necessary given the extensive evidence found at Stage 1. Our review was produced with stakeholder engagement on a project advisory board constituting personnel from key UK government departments, colleagues at the What Works Centre for Wellbeing, local and regional public health experts, and community groups. Stakeholder engagement consisted of an inception meeting to agree the protocol for the review and ongoing update meetings to discuss preliminary findings, implications of the evidence, and translation and dissemination activities.

### 2.2. Inclusion Criteria

We used a bespoke framework, PEEST (Participants, Exposure, Evaluation, Study Design, Theory), to identify relevant literature. The inclusion criteria were agreed through peer review with our stakeholder group to reflect the focus of their work on loneliness on adults aged 16 or over and from high- or middle-income countries, using the United Nations (UN) criteria, and including both clinical and community populations. The included studies evaluated participants’ exposure to or experiences of loneliness, however conceptualised, in any setting, reported as outcomes, processes, or both. Any qualitative or mixed-methods study with qualitative component in English, published between 1945 and November 2018 (for the previous review) and December 2018 and December 2020 (for the new review) was eligible if employing an identified theory, model, concept, or framework for understanding loneliness beyond a simple definition. Studies were considered off-PEEST if they included only a simple definition of loneliness with no conceptual analysis or were background literature reviews.

### 2.3. Data Sources and Search Strategy

The search strategy was informed by engaging in the peer review process with our stakeholder group, bringing expertise in policymaking, practice, and academic work on loneliness, the librarians at Brunel University London and the University of Brighton, and through advice from a systematic review expert, in accordance with PRISMA guidelines [18] for reviews in public health. Full details of our search strategy are in Supplementary S1. Eight electronic databases (Scopus, Medline (via Ovid), Eric, PsycINFO (via EBSCO), CINAHL Plus, and the Science, Social Science, Arts, and Humanities Citation Indices (via Web of Science) were searched using a combination of MeSH terms and text words. All database searches were framed by this strategy, but individual searches were appropriately revised to suit the precise requirements for each database. We hand-searched reference lists of reviews and systematic reviews published between 1945 and 2020, following PRISMA guidelines [18]. Grey literature was sought via an online call for evidence, employment of expert input, review of key-sector websites, and a Google search (a keyword search and review of titles of the first 100 hits) for the previous and new reviews.

### 2.4. Study Selection

Search results (titles and abstracts) were independently checked by two review authors. Where eligibility was unclear, the full article was checked. Disagreements were resolved through consensus, or a third team member considered the citation and a majority decision was made.

### 2.5. Data Extraction

We extracted data as reported by authors on: (a) the conceptualisation(s) of loneliness, (b) population defined by age, identity, or context, (c) positive and negative attributes of loneliness identified, and (d) mechanisms for alleviating loneliness. Data were extracted onto standardised forms independently by one reviewer and cross-checked by a second. Discrepancies were resolved by consensus. Our protocol allowed us to contact authors if the required information could not be extracted and if this was essential for interpretation of their results, but we did not need to follow this procedure.

### 2.6. Quality Assessment

The Critical Appraisal Skills Programme (CASP) quality checklist for qualitative studies was used for published studies [20]. Two authors independently applied the criteria to each included study, and disagreements were resolved through discussion. For grey literature, the Public Health England (PHE) Arts for Health and Wellbeing Evaluation Framework [21] was used to judge the quality in terms of the appropriateness of the evaluation design, the rigour of the data collection and analysis and the precision of the reporting. This checklist was employed by agreement with stakeholders in the project on account of the methods and context for collecting evidence in the grey literature being less well established than the published literature.

Confidence in the Evidence of Reviews of Qualitative Research (CERQual) was used to judge confidence in the review findings, specifically the methodological limitations, relevance, coherence, and adequacy of the data [22]. Confidence was decreased if there were serious or very serious limitations in the design or conduct of the study, the evidence was not relevant to the study objectives, the findings/conclusions were not supported by the evidence, or the data were of inferior quality and inadequate in supporting the findings. Confidence was increased if the study was well designed with few limitations, the evidence was applicable to the context specified in the objectives, the findings/conclusions were supported by evidence and provided convincing explanation for any patterns found, or the data supporting findings were rich and of high quality.

### 2.7. Data Synthesis

The synthesis was conducted thematically using the principles of inductive and reflexive methods (see for example [23]) through the development of a broadly inductive and iterative framework or typology that identified three types of loneliness: (i) social loneliness, (ii) emotional loneliness, and (iii) existential loneliness. This framework was drafted by L.M. and N.D. and refined by the research team. Each paper was categorised as representing one or more of the types of loneliness, bringing a more nuanced understanding to the analysis, anchored in a recognition of the complex social, emotional, and contextual factors which characterise loneliness.

## 3. Results

### 3.1. Search Results

**Figure 1:**
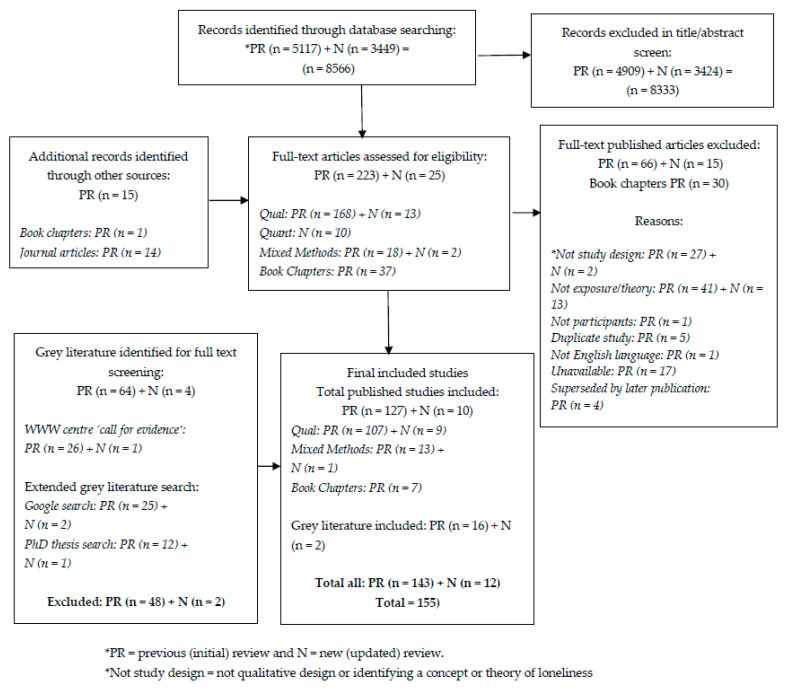
PRISMA flowchart.

The previous review returned 5117 citations after the removal of duplicates and 15 records from additional searches (total 5192) of which 223 full texts were assessed for eligibility. From the previous review, 127 published studies and 16 grey literature reports (total 143) were included. The new review returned 3449 citations after the removal of duplicates, of which 25 full texts were assessed for eligibility. There were 10 published studies and 2 grey literature reports that were included from the new review (a total of 12). In total, 137 published studies and 18 grey literature reports are included in this systematic review, providing 155 sources of evidence conceptualising loneliness: 116 qualitative studies in journal articles and 7 book chapters (including interviews, observation, document analysis, diaries, and focus group methods); 14 mixed-methods studies (only the qualitative findings met inclusion criteria); and 18 grey literature reports. The search-screening process is illustrated in the PRISMA flowchart (see Figure 1). A table of excluded studies with reasons for exclusion can be found in Supplementary S2.

### 3.2. Study Characteristics

A summary of the characteristics of included studies can be found in a list in Supplementary S3 (with full details of study objectives, description, participants, design and analysis employed, how it conceptualises loneliness, the predominant themes, and study conclusions). In terms of populations, 68 studies reported that participants were solely aged 50+ years (43%). However, the included studies also demonstrated considerable heterogeneity including groups specified by youth and middle age, cultural, ethnic, gender and sexual orientation, people living with physical and mental illness, those living in care homes, people in clinical settings, homeless people, healthcare professionals, volunteers, parents, and prisoners. Studies were reported for 26 different countries with the largest representation from the UK (*n* = 37) and the USA (*n* = 33), and the earliest published study, by Jerrome, was dated 1983. Loneliness was conceptualised in the included studies principally by three types: social loneliness (*n* = 108), emotional loneliness (*n* = 27), and existential loneliness (*n* = 20). Studies emphasised one of these three types of loneliness, and some considered the interconnections between two or more different types, which we discuss later in the section on multidimensional concepts of loneliness.

### 3.3. Study Quality

Following the CASP quality checklist, articles were scored out of 8, where 8 is the highest. In general, the quality was good for the published journal articles, with a relatively high number of studies (73/155) receiving scores of 7 and 8, but less so for the book chapters, most likely due to the different publishing requirements. Study quality varied across the types of loneliness studies with ratings of 7 or 8 for 48% of the social loneliness (52/108), 65% of existential loneliness (13/20), and 37% of emotional loneliness (10/27) studies. Methodological weaknesses included a lack of exact details of the researcher’s role, potential bias, and influence on the sample recruitment, setting, and responses of participants. Published studies identified ethical issues but did not always include an official record. The grey literature was of mixed quality with high-quality reports, including details of methods, theoretical analysis and recognition of limitations, and low-quality (credibility) reports providing little detail of the methods, commonly taking participant accounts at face value without theoretical analysis. A summary of quality checklist results and scores is presented in Supplementary S4 for published studies. Supplementary S5 presents quality ratings for the grey literature. The CERQual qualitative evidence profile is shown in Supplementary S6 (providing a succinct summary of the methodological limitations of all the included studies). This shows that there was much more evidence for social loneliness than emotional and existential loneliness, and we have high confidence in the social loneliness results and moderate confidence in the emotional and existential loneliness results.

### 3.4. Social Loneliness

Studies of social loneliness dominate the evidence accounting for 70% (108/155) of sources in the review and included populations of different ages, family carers, and a range of different employment groups [24,25,26,27,28,29,30,31,32,33,34,35,36,37,38,39,40,41,42,43,44,45,46,47,48,49,50,51,52,53,54,55,56,57,58,59,60,61,62,63,64,65,66,67,68,69,70,71,72,73,74,75,76,77,78,79,80,81,82,83,84,85,86,87,88,89,90,91,92,93,94,95,96,97,98,99,100,101,102,103,104,105,106,107,108,109,110,111,112,113,114,115,116,117,118,119,120,121,122,123,124,125,126,127,128,129,130,131]. The typical conceptualisation of social loneliness was as an ‘objective’ condition framed by numbers of social connections and explained as a subjective evaluation of feeling isolated, deprived of companionship, lacking a sense of belonging, and lacking access to a satisfying social network; that is, it describes a sense of disconnection from others. How this concept was manifest and the underpinning contributary mechanisms varied across populations and contexts.

In studies focused on older 50+ years and younger populations, social loneliness was articulated as a feeling of disconnection across various domains of life including devaluation, helplessness, powerlessness, and feelings of stigma and shame. The vulnerability created by social loneliness was highlighted by accounts of financial fraud in elderly people [38]. For older adults, loss, detachment, and boredom were commonly identified as contributors to loneliness in both community and care home settings. Young people noted loneliness to be connected to the need to escape from someone or something and aligned loneliness with submission or resignation to negative feelings, often alongside feelings of shame and stigma [67]. The stigma of loneliness was reported by a wide range of groups, including people with HIV and with cancer [24], homeless people [25,32,74,111], female prisoners [43], men who have sex with other men [53], transgender people [108], and older people living in care settings [29]. To clarify, within the context of loneliness, stigma is understood to mean some kind of marginalisation, feelings of disgrace, or exclusion. If the papers did not directly use the term ‘stigma’ but used one of these concepts, we took it to mean stigmatisation.

There were groups or contexts where the feeling of disconnection associated with social loneliness took specific forms. Cultural difference was reported as a potential source of social loneliness in older population groups [69,82]. For international students, the absence of intimate personal connections combined with a lack of cultural fit created ‘cultural loneliness’, a version of social loneliness generated by the absence of their cultural and linguistic settings [112]. This notion of cultural loneliness could persist even when people had good access to social networks. Studies of social loneliness in the employment context identified a lack of support and employment-related ‘distancing’ or isolation from others from a diverse range of employees including long-haul truck drivers [26], homeworkers [46], school principals [57,60,113], medical educators [96], professional golfers [40], and senior corporate managers [104], as well as family caregivers [45,98]. Homeworkers and family caregivers seem to be especially vulnerable. Both groups experienced a sense of being cut off from their networks, professional and personal, because of the restrictions their respective roles had on their personal life [46,98]. For family caregivers, not feeling understood and being denied recognition for their role was experienced alongside more generic feelings of powerlessness and helplessness to take control of live events.

Experiences of illness and healthcare led to or compounded social loneliness in ways not reported for other population groups. In care homes, feeling lonely was exacerbated by issues preventing carers from providing adequate care (e.g., limited resources, time pressures, and professional rules) [83]. For stroke patients, a lack of support and contact, a sense of being unable to contribute, and not having an intimate relationship contributed to loneliness [109]. The design of healthcare environments can generate social loneliness as illustrated by a stroke ward, which while allowing privacy and supporting efficient clinical care, increased loneliness and created barriers to social connection [25]. This example provides one of the few explanations of loneliness not simply attributable to individual characteristics. In mental health contexts, social loneliness was affected by external environments, activities, and therapies/treatment, as well as people [121]. Social loneliness arose as a result of physical, cognitive, behavioural, and emotional responses following traumatic brain injury since these changes could affect existing relationships, leading to the loss of old friends and creating difficulties in making new ones [85].

Solutions to social loneliness were numerous and diverse in this evidence base. For older adults, services to alleviate social loneliness were largely focussed on increasing social contacts. These included friendship clubs [44], music provision [127], museum-based social prescribing [129,131], local history cafes [119], broadly defined community-based approaches [30,58,89,100,125], and health-messaging services [49,124]. Community-based approaches to alleviating social loneliness were explored where community leaders worked with older women [100]. These suggested that increasing independence, improving communication and developing mentoring, buddying, and intergenerational befriending programmes could provide relevant support to women of older age [100]. Intergenerational approaches using reverse mentoring in which younger adults trained older people in the use of information technology (IT) were reported as successful in alleviating self-reported social loneliness in older people in one study [33]. Provision of social activities to alleviate social loneliness in nursing homes could include a range of activities (e.g., self-awareness programmes, humour sessions, social engagements, and faith-based activities), but these were only associated with self-reported reductions in social loneliness if the activities were relevant to older people [72,80].

Young people used a variety of coping strategies for managing social loneliness and preserving and extending social connections. These included distraction, seeking help from professionals and institutions, support seeking, self-reliance, and problem-solving behaviours [67,112,118]. Although social loneliness in young people was difficult to identify, youth workers could help to prevent a downward spiral by addressing loneliness risk at key moments, which would differ amongst individuals, but may be related to relationship concerns, mental health issues, and a range of perceived stressors in life [128].

Studies reported a range of strategies for addressing workplace social loneliness in different contexts, including provision of opportunities to socialise and maintaining connections with people who provide social support [26]. The use of mobile technologies, such as smartphones, widened possibilities for homeworkers to socialise while retaining access to emails and remaining contactable by clients, although the use of technological devices did not necessarily address professional isolation in homeworkers [46]. Strategic responses to alleviating social loneliness at the organisational level were noted in the context of academic institutions as part of a wider examination of the role of social support in improving mental wellbeing [6]. In wider work contexts, the extent to which senior managers felt lonely was also dependent on coping strategies they used, including mental and physical disconnection, adopting a healthy lifestyle, gaining support from one’s network, and affecting and influencing others.

### 3.5. Emotional Loneliness

A total of 27 of the 155 included studies conceptualised emotional loneliness with a ‘loss model’ or ‘primary relationship deficit’ approach, which was used in all but two of the studies as the explanatory framework [132,133,134,135,136,137,138,139,140,141,142,143,144,145,146,147,148,149,150,151,152,153,154,155,156,157,158]. A common theme in conceptualisations of emotional loneliness was the connection with social isolation and a loss or lack of good quality social relationships in all the included studies in this theme. However, in contrast to social loneliness, the sense of loss, disconnection, withdrawal, detachment, or alienation from people and places and feelings of abandonment and exclusion was resultant from a lack of a sense of belonging or recognition and, for older adults, perceptions of agism and stereotyping. Negative emotions identified in conceptualisations of emotional loneliness included sadness, fear, anxiety, and worry. Although it overlaps with social loneliness, the emotional aspect within this conceptualisation of loneliness requires addressing in a distinct way because these negative feelings occur even when one is in close contact with people. Positive emotions connected to emotional loneliness were also conceptualised in terms of optimistic perceptions of aloneness and solitude associated with learning to cope with loneliness and adjusting to imposed loneliness. Emotional loneliness was reported as both acute, temporary, and subject to negotiation and change, but also permanent, long-lasting, and associated with detrimental mental and physical health.

Emotional loneliness was described by older people as a type of inner pain or suffering, a feeling to be kept hidden and silent because of fears of being stigmatised as lonely and old, becoming a burden on family and friends, and feeling responsible for controlling emotional aspects of loneliness [140,141,150,151]. Negative feelings associated with loss [133,134,135,137,140,141,150,154,156], disconnection, withdrawal, detachment, or alienation from people and places [135,150,152,156], a feeling of abandonment [137,143], exclusion [141], and a sense of losing or being in conflict with ones’ established identity [135,140,144,145] were descriptions of emotional loneliness in old age. Two studies focussed on young people, highlighting a dearth of studies in this area [139,149]. Both studies related to young people growing up in particular family contexts: living with a parent diagnosed with cancer and having parents who were Holocaust survivors, highlighting specific exclusionary contexts. For the parental cancer patient context, key exclusionary processes included failure of professionals to explain the situation to them, being left out of decisions and conversations connected to diagnoses, and treatment of their parents. Alongside this, feelings of uncertainty about the future, fear of losing a parent, and a sense that they were not equipped to cope generated a sense of distance or disconnection from others. For children of parents who were Holocaust survivors, emotional loneliness was a cognitive reaction to parental trauma [149] and a consequence of negative self-comparison to families without such trauma. Alleviating this kind of emotional loneliness was associated with support and comfort offered by family members and being given accurate information by healthcare professionals, which combined to provide a sense of relief from emotional loneliness for the young people.

Positive feelings associated with emotional loneliness were observed only for older adults and were associated with the perceived benefits of solitude or aloneness [137,143,147]. Solitude was connected to feelings of freedom and a sense of comfort in old age in one study and defined as an ‘at homeness’. Being able to cope with emotional loneliness in old age was associated with a sense of joy and pride in oneself [147] and emotional self-management [143]. The included studies examined processes of negotiating and adapting to loneliness in old age. Alleviating emotional loneliness was connected to establishing new routines to account for the loss of loved ones or social networks [135,152], including developing opportunities for meaningful social contact [156], engaging in therapy for those living with long-term mental health conditions [141], and taking part in meaningful activities, such as reading, gardening, and social meals [143,151].

Of the 27 studies on emotional loneliness, seven studies explored emotional loneliness as a consequence of relational issues [132,136,138,142,148,153,155,157], further supporting the idea that social and emotional loneliness are interconnected. Four of these identified that complex family dynamics including responsibility for childcare, older children leaving home, and bereavement (for mothers) [132]; poor childhood attachment (to fathers), and weak sibling relationships [153]; being placed in a care home [148]; and loss of intimate partner relationships [136,155]. These all resulted in the deeply emotional character of loneliness connected to low mood, a lack of sense of purpose and feelings of suffering, family abandonment, and pervasive worry. Such negative consequences of emotional loneliness could be further entrenched by disadvantaged living conditions, such as low income and limited access to spaces/places with good amenities [132]. This evidence points to the significance of high quality and meaningful relationships and an understanding of the role of place/space in countering social and emotional loneliness. When relationships (particularly intimate ones) are not high quality, there is a negative impact on emotional loneliness, shaped through feelings of disappointment, abandonment, and feeling devalued or powerless [157]. In studies of people living with learning disabilities or mental health conditions [142,146], the structuring of exclusion was identified as a contributory factor to emotional loneliness, characterised by a sense of exclusion and lack of acceptance even in the presence of people. In one of these studies, the potential of reframing loneliness in terms of the benefits of solitude was considered possible [142].

Approaches to alleviating emotional loneliness for those living with physical and mental health conditions included nostalgic activities, allowing remembering and reminiscing about happy times, keeping busy, and engaging in activities involving meeting people, which could lead to feelings of pride in self-management [141,147,151,152]. Participating in therapy was also considered to help strengthen a sense of connection to the world and alleviate emotional loneliness for those living with mental health conditions [141]. Alleviating emotional loneliness associated with insecurity or a lack of attachment to place, either rural or urban, through place-based strategies was reported in one study [154]. Creating place-based opportunities for the development of a sense of neighbourhood was similarly suggested in one study as a potential solution to emotional loneliness [156].

### 3.6. Existential Loneliness

A total of 20 the 155 included studies conceptualised existential loneliness [159,160,161,162,163,164,165,166,167,168,169,170,171,172,173,174,175,176,177,178]. In contrast to emotional or social loneliness, existential loneliness was defined as a feeling of fundamental separateness from others and the wider world, not simply as the absence of meaningful relationships and negative emotional experience. Participants described it as a feeling that occurred when important others were absent through some form of psychological rejection or absence [159,161,162], or when people felt left behind by life events, such as death or divorce, and/or experiences of physical or mental decline or limitation through illness, traumatic experience, aging, and a sense of one’s mortality [160,163,164,165,166,167,168,169,170,171,172,173,174,176,177,178]. Similar explanations of existential loneliness were reported by healthcare professionals supporting older people experiencing this type of loneliness [175]. This conceptualisation of existential loneliness was connected, in all studies examining the concept, to feelings of separateness from other human beings, feelings of loss and longing, and/or a sense of being an outsider against a need for connectedness, belonging, and companionship. Studies indicated that existential loneliness may also be felt while with others, as a sense of disconnection from a group [159,161,163,167,168,170,171,172,174]. In such situations, participants reported that existential loneliness was experienced through being misunderstood, psychologically and emotionally detached, and more deeply as a perception of being without others. For AIDS patients in one study, this was shown to lead to stigma for this population group [159]. Negative feelings of existential loneliness were connected to healthcare contexts via the concept of the ‘lonely patient’ who, while in close proximity to other patients or healthcare professionals, may feel disconnected because of a sense of vulnerability, lack of care [161,163], or issues with communication [169].

Existential loneliness was not always conceived of as a negative experience. Evidence in one study suggested that existential loneliness could be meaningful if developed as part of a voluntary transcendental experience [161]. Such self-directed and potentially positive experiences of existential loneliness were conceptualised as a powerful force for calm and peace, a type of temporary recharging experience adopted when people felt a need to break from human connection for a while. While old age, frailty, and impending death were most often considered in the context of negative feelings of existential loneliness, there was evidence in two studies that such life experiences contributed to an understanding of loneliness as a balance between solitude and meaningful human connections that involved both social and emotional experiences, and the building of new and trusting relationships [173,177].

### 3.7. Multidimensional Models of Loneliness

Perhaps representing an advance in understandings of loneliness, six studies in this review proposed what we would refer to as multidimensional models of loneliness [29,37,63,69,82,94]. Multidimensional models of loneliness in studies of older people reflected the influence of interactions between relationship quality, becoming and being old, personal troubles/personality traits, and sickness on feelings of loneliness [29,37,94]. In these models, social loneliness intertwines with emotional loneliness in complex ways revealing loneliness to be deeply or intensely felt, potentially hidden or masked, and diverse in experience. In two studies, the complexity of social loneliness was explored in terms of cultural difference and diversity [69,82]. Social loneliness was conceptualised as embodied and culturally nuanced in terms of physical, emotional, and spiritual expression [69]. Disrupted cultures and communities were linked with diverse cultural experience of loneliness [82]. Severe mental illness (SMI) had the effect of reducing or changing social networks even though many people with SMI desired to have greater social networks [93]. Social loneliness was also characterised as emotionally as well as socially excluding and reported as the feeling of being somehow removed from life [63]. Such findings demonstrate some overlap with existential types of loneliness for those living with mental health conditions [63].

## 4. Discussion

### Principal Findings and Contribution to Knowledge

The findings in this review support calls for better understandings of the complexity of loneliness in different population groups and social contexts [179,180] to inform policymakers and practitioners in the field. We have thematically synthesised the evidence for three types of loneliness: social, emotional, and existential. This supports but significantly develops a preliminary typology of loneliness, focused only on loneliness in healthcare research [181].

In summary of our findings, social loneliness describes the perception of dissatisfaction with the quality of relationships [182] and as a discrepancy between the actual and desired quantity and quality of social interactions [28,133,183]. This type of loneliness most closely reflects established ways in which loneliness is defined and measured in the current literature, where it is understood as a negative experience in which our social relations are deficient in some way, quantitively or qualitatively [184]. It also reflects the evidence showing correlations between loneliness and poor physical and mental health [5,14].

Emotional loneliness arises from the absence or loss of meaningful relationships, possibly of a primary attachment figure, such as a spouse. Emotional loneliness is also a consequence of a loss of health and social opportunities that are not easily replaceable. It is also interconnected with not meeting the need to be recognised and to belong. This type of loneliness reflects the extant literature, which identifies the connection between feeling lonely and distressed, emptiness, and loss [135]. Moreover, existential loneliness describes an expression of separateness from others that can occur at any time, but particularly so when facing life threatening illnesses, trauma, and one’s mortality [159,185].

Central to our findings is the implication that unlike social loneliness, emotional and existential loneliness may not be relieved by interventions focused on social connectedness and integration into a social community. Hence, a key finding from this review is that it provides a more nuanced understanding of loneliness, which can inform a targeted approach to alleviating loneliness, particularly in policy and intervention work.

Our review identifies some key gaps in the literature on loneliness. We found only two published studies focussed on the link between emotional loneliness in young people living with a parent with cancer [139]; children of Holocaust survivor parents, [149]). Innovative work reported in the grey literature shows that emotional loneliness in youth is complex and associated with a variety of life experiences [118,186]. Indeed, feelings of loneliness arise during key moments of transition in life, and they are unlikely to be fully captured by the current static and unidimensional definitions that solely focus on the quality and quantity of social relationships. This links to another key gap we found in that there is a dearth of studies on loneliness across the life course since this could show how loneliness is experienced distinctly at different points in time and through different life events [187,188,189] The review also identified a lack of studies on loneliness and inequalities, such as socio-economic status and disability, since the included studies present thin demographic data. This, therefore, provides a direction for future research in this area.

More specifically, our review also highlights the importance of conceptualising existential loneliness in order to design effective interventions in circumstances and contexts in which people feel hopelessly detached from social life or misunderstood [190]. The extant literature on existential loneliness has predominantly focussed on chronic illness [172] and end-of-life care [181]. However, our review reveals other contexts in which existential loneliness requires attention. In studies of mental health, existential loneliness appears to be conceived as the sense of physical separation from others, sometimes as a development from having a disturbed self-image or as a consequence of being unable to form social connections, resulting in feelings of exclusion from normal life, stigma, emptiness, and being an outsider.

Each type of loneliness and the potential overlaps between them illustrates the diverse and complex interplay between social, psychological, and contextual factors which contribute to loneliness and potentially its alleviation in people’s lives. Understanding these conceptual differences demonstrates the need for theoretical development in multidimensional studies of loneliness, critical reviews of and methodological developments in both how loneliness is measured, and the interventions that need to be designed and implemented. At present, the current definition of loneliness is not sufficiently broad in its scope or sophisticated in its conceptualisation to capture the range, diversity, and depth of experiences that people define as lonely [17,191]. It also often confuses loneliness, a subjective phenomenon, with social isolation, an objective condition arising from quantitatively diminished social networks [28,44]. Imprecise definitions and, indeed, generalised and broad measures will limit understanding of the impact of loneliness on health.

Most studies in our review focused on social loneliness (*n* = 108), reflecting the current academic focus. Using the CERQual rating, we have high confidence in this evidence. However, there was a dearth of studies examining emotional loneliness (*n* = 27) and existential loneliness (*n* = 20), and both these types of loneliness have been found to be associated with negative health outcomes. This reflects both the more recent emergence of these two types of loneliness as distinct conceptual and empirical entities. The most established measures of loneliness implicitly or explicitly focus on social loneliness. These findings suggest a need for detailed studies of these different types of loneliness and points to the scope for public health policy and practice developments rooted in an understanding of loneliness types, interactions, and settings. We need to comprehend more clearly who feels lonely, when, where, and in what context.

## 5. Conclusions

### 5.1. Strengths and Limitations

The rigorous and systematic search strategy and comprehensive nature of this review is a strength. We focused on loneliness only and did not include social isolation and other similar concepts. The pre-publication of our protocol on PROSPERO ensured methodological transparency and mitigated any potential post-hoc decision-making, which may have introduced bias. Dual screening of the searches and data extraction and independent quality assessment of the included reviews ensured a rigorous process.

Systematic reviews and evidence syntheses of conceptual frameworks and models are unusual, and methods for such reviews are not yet well-developed. Our use of a PEEST inclusion criteria meant that the types of studies we were interested in were clear to all team members. The focus on concepts, models, theories, and frameworks of loneliness means that it is possible that some relevant evidence was not included. Seventeen potentially includable studies were unavailable, and they may have influenced findings had they been available. There is also a potential risk of publication lag where possible important new evidence has not yet been included in published articles and reports, and thus not identified and included. The grey literature search was used to mitigate that risk in part. The use of the CERQual criteria also acts as a checklist as much as an evaluative tool or measure of quality. A consistent approach to judgements across the different interventions has been applied, but it should be recognised that these judgements are open to interpretation.

### 5.2. Implications for Policymakers and Future Research

Our review identified an extensive qualitative literature conceptualising social, emotional, and existential loneliness, providing theoretical frameworks for understanding loneliness that are adaptable for decision-making in policy and practice. The literature is dominated by research conceptualising social loneliness, which understates the potential significance of emotional and existential loneliness. We suggest that a parallel review evaluating how loneliness is conceptualised in our commonly used measurement tools would be valuable.

Improved measures and high-quality mixed-methods studies would help to address this. We would recommend rigorous and systematic quantitative methods, longitudinal process evaluations, and cost effectiveness evaluations alongside appropriate qualitative methodologies and analytical techniques. Research, policy, and practice approaches to loneliness can be enhanced through co-production methods involving mutually beneficial working practices in service design, implementation, and evaluation.

### 5.3. Concluding Remarks

This systematic review summarises all the available qualitative literature on conceptualising social, emotional, and existential loneliness in diverse adult populations. Our evidence base would benefit from an improved understanding of these different types of loneliness, their inter-relationships, and how these may vary across the life course. Such evidence would underpin more effective decision-making and intervention development and influence developments in measuring loneliness. This would enable policymakers and practitioners to determine what intervention would work, for whom, and in what context.

Our findings showed that there is a lack of detail in the literature on loneliness and inequalities (gender, ethnicity, disability, and socio-economic status) because of limited reporting of demographic data in the included studies. There remains, therefore, a need for future rigorous research on loneliness and inequality to understand how loneliness may be experienced differently and thus how to address the detrimental health effects of the interaction of these issues. The findings will be useful for decision-making in public policy and practice that seeks to identify who is lonely, why loneliness occurs, how it is experienced, and the most effective ways to alleviate loneliness or optimise the positive aspects of solitude for enhanced health and wellbeing outcomes. If public health interventions and policy decisions for alleviating loneliness are not based on accurate definitions and understandings, it is unlikely that they will be effective.

## Data Availability

All summaries of data are available in the Supplementary Material.

## Supplementary Materials

The following are available online at https://www.mdpi.com/article/10.3390/ijerph182111522/s1, Supplementary S1: Search strategy; Supplementary S2: Table of excluded studies; Supplementary S3: Characteristics of included studies conceptualising loneliness (published and unpublished literature); Supplementary S4: Quality checklist scores for qualitative studies (published); Supplementary S5: Quality ratings for grey (unpublished) studies; Supplementary S6: CERQual qualitative evidence profile.

## References

[1] Rotenberg K., Rotenberg K., Hymel S. (1999). Childhood and adolescent loneliness: An introduction. Loneliness in Childhood and Adolescence.

[2] Mijuskovic B. (1981). Loneliness and human nature. Psychol. Perspect..

[3] Russell D., Peplau L.A., Cutrona C.E. (1980). The revised UCLA Loneliness Scale: Concurrent and discriminant validity evidence. J. Pers. Soc. Psychol..

[4] Smith K.J., Victor C. (2018). Typologies of loneliness, living alone and social isolation, and their associations with physical and mental health. Ageing Soc..

[5] Gerst-Emerson K., Jayawardhana J. (2015). Loneliness as a public health issue: The impact of loneliness on health care utilization among older adults. Am. J. Public Health.

[6] Holt-Lunstad J., Smith T.B., Baker M., Harris T., Stephenson D. (2015). Loneliness and social isolation as risk factors for mortality: A meta-analytic review. Perspect. Psychol. Sci..

[7] Christensen A.V., Juel K., Ekholm O., Thrysøe L., Thorup C.B., Borregaard B., Mols R.E., Rasmussen T.B., Berg S.K. (2020). Significantly increased risk of all-cause mortality among cardiac patients feeling lonely. Heart.

[8] Kharicha K., Iliffe S., Harari D., Swift C., Gillmann G., Stuck A.E. (2007). Health risk appraisal in older people 1: Are older people living alone an “at-risk” group?. Br. J. Gen. Pract..

[9] Perissinotto C.M., Stijacic Cenzer I., Covinsky K.E. (2012). Loneliness in older persons: A predictor of functional decline and death. Arch. Intern. Med..

[10] Cacioppo J.T., Hawkley L.C. (2009). Perceived social isolation and cognition. Trends Cogn. Sci..

[11] Zunzunegui M.V., Alvarado B.E., Del Ser T., Otero A. (2003). Social networks, social integration, and social engagement determine cognitive decline in community-dwelling Spanish older adults. J. Gerontol. B Psychol. Sci. Soc. Sci..

[12] Park B., Kim S.Y., Shin J.Y., Sanson-Fisher R.W., Shin D.W., Cho J., Park J.H. (2013). Prevalence and predictors of anxiety and depression among family caregivers of cancer patients: A nationwide survey of patient-family caregiver dyads in Korea. Support. Care Cancer.

[13] Hawthorne G. (2008). Perceived social isolation in a community sample: Its prevalence and correlates with aspects of peoples’ lives. Soc. Psychiatry Psychiatr. Epidemiol..

[14] Leigh-Hunt N., Bagguley D., Bash K., Turner V., Turnbull S., Valtorta N., Caan W. (2017). An overview of systematic reviews on the public health consequences of social isolation and loneliness. Public Health.

[15] Windle K., Francis J., Coomber C. (2011). Preventing loneliness and Social Isolation: Interventions and Outcomes.

[16] Courtin E., Knapp M. (2017). Social isolation, loneliness and health in old age: A scoping review. Health Soc. Care Community.

[17] Victor C., Mansfield L., Kay T., Daykin N., Lane J., Grigsby Duffy L., Tomlinson A., Meads C. An Overview of Reviews: The Effectiveness of Interventions to Address Loneliness at All Stages of the Life-Course. What Works Wellbeing. https://whatworkswellbeing.org/wp/wp-content/uploads/woocommerce_uploads/2018/10/Full-report-Tackling-loneliness-Oct-2018.pdf.

[18] Moher D., Liberati A., Tetzlaff J., Altman D.G. (2009). PRISMA Group. Preferred reporting items for systematic reviews and meta-analyses: The PRISMA statement. PLoS Med..

[19] Walsh K., Scharf T., Keating N. (2017). Social exclusion of older persons: A scoping review and conceptual framework. Eur. J. Ageing.

[20] CASP CASP Checklists. https://casp-uk.net/casp-tools-checklists/.

[21] Daykin N., Joss T. Public Health England Arts and Health Evaluation Framework. https://www.gov.uk/government/uploads/system/uploads/attachment_data/file/496230/PHE_Arts_and_Health_Evaluation_FINAL.pdf.

[22] Lewin S., Glenton C., Munthe-Kaas H., Carlsen B., Colvin C.J., Gülmezoglu M., Noyes J., Booth A., Garside R., Rashidian A. (2015). Using qualitative evidence in decision making for health and social interventions: An approach to assess confidence in findings from qualitative evidence syntheses (GRADE-CERQual). PLoS Med..

[23] Braun V., Clarke V., Hayfield N., Terry G., Liamputtong P. (2018). Thematic Analysis. Handbook of Research Methods in Health Social Sciences.

[24] Adams R., Mosher C., Abonour R., Robertson M., Champion V., Kroenke K. (2016). Cognitive and situational precipitants of loneliness. A qualitative analysis. Oncol. Nurs. Forum.

[25] Anaker A., von Koch L., Heylighen A., Elf M. (2018). It’s lonely: Patients experiences of the physical environment at a newly built stroke unit. Health Environ. Res. Des. J..

[26] Apostolopoulos Y., Sanmez S., Hege A., Lemke M. (2016). Work strain, social isolation and mental health of long-haul truckers. Occup. Ther. Ment. Health.

[27] Ballin L., Balandin S. (2007). An exploration of loneliness: Communication and the social networks of older people with cerebral palsy. J. Intellect. Dev. Disabil..

[28] Bantry-White E., O’Sullivan S., Kenny L., O’Connell C. (2018). The symbolic representation of community in social isolation and loneliness among older people: Insights for intervention from a rural Irish case study. Health Soc. Care Community.

[29] Barbosa N., Sanders A., Kokanović R. (2019). “It’s the worst bloody feeling in the world”: Experiences of loneliness and social isolation among older people living in care homes. J. Ageing Stud..

[30] Barke J. (2017). Community-based research and approaches to loneliness prevention. Work Older People.

[31] Bess K., Doykos B. (2014). Tied together: Building relational well-being and reducing social isolation through place-based parent education. J. Community Psychol..

[32] Bower M., Conroy E., Perz J. (2017). Australian homeless persons experiences of social connectedness, isolation and loneliness. J. Health Soc. Care Community.

[33] Breck B., Dennis C., Leedahl S. (2018). Implementing reverse mentoring to address social isolation among older adults. J. Gerontol. Soc. Work.

[34] Canham S. (2015). What’s loneliness got to do with it? Older women who use benzodiazepines. Australas. J. Ageing.

[35] Cela E., Fokkema T. (2017). Being lonely later in life: A qualitative study among Albanians and Moroccans in Italy. J. Ageing Soc..

[36] Cloutier-Fisher D., Kobayashi K., Smith A. (2011). The subjective dimension of social isolation: A qualitative investigation of older adults’ experiences in small social support networks. J. Aging Stud..

[37] Cohen-Mansfield J., Eisner R. (2020). The meanings of loneliness for older persons. J. Aging Ment. Health.

[38] Cross C. (2016). ‘They’re very lonely’: Understanding the fraud victimisation of seniors. Int. J. Crime Justice Soc. Democr..

[39] Esposito M. (2015). Women in prison: Unhealthy lives and denied well-being between loneliness and seclusion. J. Crime Law Soc. Chang..

[40] Fry J., Bloyce D. (2017). ‘Life in the travelling circus’: A study of loneliness, work stress, and money issues in touring professional golf. Sociol. Sport J..

[41] Goll J., Charlesworth G., Scior K., Stott J. (2015). Barriers to social participation among lonely older adults: The influence of social fears and identity. PLoS ONE.

[42] Hauge S., Kirkevold M. (2012). Variations in older persons’ descriptions of the burden of loneliness. Scand. J. Caring Sci..

[43] Heenan D. (2011). How local interventions can build capacity to address social isolation in dispersed rural communities: A case study from Northern Ireland. Ageing Int. J..

[44] Hemingway A., Jack E. (2013). Reducing social isolation and promoting well being in older people. Qual. Ageing.

[45] Hinton W., Levkoff S. (1999). Constructing Alzheimer’s: Narratives of lost identities, confusion and loneliness in old age. J. Cult. Med. Psychiatry.

[46] Hislop D., Axtell C., Collins A., Daniels K., Glover J., Niven K. (2015). Variability in the use of mobile ICTs by homeworkers and its consequences for boundary management and social isolation. J. Inf. Organ..

[47] Hollenbeck C., Patrick V. (2017). Alleviating survivor loneliness: The value of expressive gift systems in peer-to-peer online patient survivor networks. Rev. Mark. Res..

[48] Holtz C., Sowell R., Velasquez G. (2012). Oaxacan Women with HIV/AIDS: Resiliency in the face of poverty, stigma, and social isolation. J. Women Health.

[49] Honigh-de Vlaming R., Haveman-Nies A., Ziylan C., Renes R. (2013). Acceptability of the components of a loneliness intervention among elderly Dutch People: A qualitative study. Am. J. Health Educ..

[50] Houston K. (2016). Social isolation and health in widowhood: A qualitative study of Nepali widows’ experiences. Health Care Women Int..

[51] Howard A., Tan de Bibiana J., Smillie K., Goddard K., Pritchard S., Olson R., Kazanjian A. (2014). Trajectories of social isolation in adult survivors of childhood cancer. J. Cancer Surviv..

[52] Howard A., Agllias K., Bevis M., Blakemore T. (2018). How social isolation affects disaster preparedness and response in Australia: Implications for social work. Aust. Soc. Work.

[53] Hubach R., DiStefano A., Wood M. (2012). Understanding the influence of loneliness on HIV risk behavior in young men who have sex with men. J. Gay Lesbian Soc. Serv..

[54] Hurtado-de-Mendoza A., Gonzales F., Serrano A., Kaltman S. (2014). Social isolation and perceived barriers to establishing social networks among Latina immigrants. Am. J. Community Psychol..

[55] Janta H., Lugosi P., Brown L. (2014). Coping with loneliness: A netnographic study of doctoral students. J. Furth High. Educ..

[56] Jerrome D. (1983). Lonely women in a friendship club. Br. J. Guid. Couns..

[57] Kelchtermans G., Piot L., Ballet K. (2011). The lucid loneliness of the gatekeeper: Exploring the emotional dimension in principals’ work lives. Oxf. Rev. Educ..

[58] Kharicha K., Iliffe S., Manthorpe J., Chew-Graham C., Cattan M., Goodman C., Kirby-Barr M., Whitehouse J., Walters K. (2017). What do older people experiencing loneliness think about primary care or community-based interventions to reduce loneliness? A qualitative study in England. J. Health Soc. Care Community.

[59] Kirkevold M., Moyle W., Wilkinson C., Meyer J., Hauge S. (2013). Facing the challenge of adapting to a life ‘alone’ in old age: The influence of losses. J. Adv. Nurs..

[60] Korumaz M. (2016). Invisible barriers: The loneliness of school principals at Turkish elementary schools. S. Afr. J. Educ..

[61] Lanyon L., Worrall L., Rose M. (2018). Combating social isolation for people with severe chronic aphasia through community aphasia groups: Consumer views on getting it right and wrong. Aphasiology.

[62] Lee H., Coenen A., Heim K. (1994). Island living: The experience of loneliness in a psychiatric hospital. J. Appl. Nurs. Res..

[63] Lindgren B., Sundbaum J., Eriksson M., Graneheim U. (2014). Looking at the world through a frosted window: Experiences of loneliness among persons with mental ill-health. J. Psychiatr. Ment. Health Nurs..

[64] Lou V., Ng J. (2012). Chinese older adults’ resilience to the loneliness of living alone: A qualitative study. J. Aging Ment. Health.

[65] Mackowicz J., Wnek-Gozdek J. (2018). Centenarians’ experience of (non-) loneliness life lessons. J. Educ. Gerontol..

[66] McHugh Power J., Hannigan C., Carney S., Lawlor B. (2017). Exploring the meaning of loneliness among socially isolated older adults in rural Ireland: A qualitative investigation. J. Qual. Res. Psychol..

[67] McLaughlin C., Sillence E. (2018). Buffering against academic loneliness: The benefits of social media-based peer support during postgraduate study. Active Learn. High. Educ..

[68] Milsom J., Beech A., Webster S. (2003). Emotional loneliness in sexual murderers: A qualitative analysis. Sex. Abuse J..

[69] Morgan T., Wiles J., Moeke-Maxwell T., Black S., Park H.J., Dewes O., Williams L.A., Gott M. (2020). ‘People haven’t got that close connection’: Meanings of loneliness and social isolation to culturally diverse older people. Aging Ment. Health.

[70] Ojembe B., Kalu M. (2018). Describing reasons for loneliness among older people in Nigeria. J. Gerontol. Soc. Work.

[71] Ozawa-de Silva C. (2008). Too lonely to die alone: Internet suicide pacts and existential suffering in Japan. J. Cult. Med. Psychiatry.

[72] Paque K., Bastiaens H., Van Bogaert P., Dilles T. (2018). Living in a nursing home: A phenomenological study exploring residents’ loneliness and other feelings. Scand. Caring Sci..

[73] Park H., Morgan T., Wiles J., Gott M. (2018). Lonely ageing in a foreign land: Social isolation and loneliness among older Asian migrants in New Zealand. J. Health Soc. Care Community.

[74] Pedersen P., Andersen P., Curtis T. (2012). Social relations and experiences of social isolation among socially marginalized people. J. Soc. Pers. Relat..

[75] Pettigrew S., Donovan R., Boldy D., Newton R. (2014). Older people’s perceived causes of and strategies for dealing with social isolation. J. Aging Ment. Health.

[76] Piat M., Sabetti J., Padgett D. (2018). Supported housing for adults with psychiatric disabilities: How tenants confront the problem of loneliness. J. Health Soc. Care Community.

[77] Pirhonen J., Tiilikainen E., Pietila I. (2018). Ruptures of affiliation: Social isolation in assisted living for older people. J. Ageing Soc..

[78] Pramuditha M. (2014). Exploring the experience of loneliness among older Sinhalese immigrant women in Canada. Perspectives.

[79] Rokach A. (1988). The experience of loneliness: A tri-level model. J. Psychol. Interdiscip. Appl..

[80] Roos V., Klopper H. (2010). Older persons’ experiences of loneliness: A South African perspective. J. Psychol. Afr..

[81] Roos V., Malan L. (2012). The role of context and the interpersonal experience of loneliness among older people in a residential care facility. J. Glob. Health Action.

[82] Roos V., Keating N., Kahl C. (2019). Loneliness of older black South African women subjected to forcible relocation. Glob. Health Action.

[83] Russell C., Schofield T. (1999). Social isolation in old age: A qualitative exploration of service providers’ perceptions. J. Ageing Soc..

[84] Sa’ar A. (2001). Lonely in your firm grip: Women in Israeli-Palestinian families. J. R. Anthropol. Inst..

[85] Salas C., Casassus M., Rowlands L., Pimm S., Flanagan D. (2018). ‘Relating through sameness’: A qualitative study of friendship and social isolation in chronic Traumatic Brain Injury. J. Neuropsychol. Rehabil..

[86] Schirmer W., Michailakis D. (2015). The lost Gemeinschaft: How people working with the elderly explain loneliness. J. Aging Stud..

[87] Smith C. (1998). ‘Men don’t do this sort of thing’: A case study of the social isolation of househusbands. J. Men Masc..

[88] Smith J. (2012). Loneliness in older adults: An embodied experience. J. Gerontol. Nurs..

[89] Stanley M., Moyle W., Ballantyne A., Jaworski K., Corlis M., Oxlade D., Stoll A., Young B. (2010). ‘Nowadays you don’t even see your neighbours’: Loneliness in the everyday lives of older Australians. J. Health Soc. Care Community.

[90] Sullivan M., Victor C., Thomas M. (2016). Understanding and alleviating loneliness in later life: Perspectives of older people. Qual. Ageing.

[91] Taube E., Jakobsson U., Midlov P., Kristensson J. (2016). Being in a bubble: The experience of loneliness among frail older people. J. Adv. Nurs..

[92] Tiilikainen E., Seppanen M. (2017). Lost and unfulfilled relationships behind emotional loneliness in old age. J. Ageing Soc..

[93] Topor A., Ljungqvist I., Strandberg E. (2016). The costs of friendship: Severe mental illness, poverty and social isolation. Psychosis.

[94] Tuominen K., Pirhonen J. (2019). “Who would take a 90-year-old?” Community-dwelling nonagenarians’ perceptions of social relationships. Int. J. Ageing Later Life.

[95] Van Bergen D., Van Balkom A., Smit J., Saharso S. (2012). I felt so hurt and lonely. J. Transcult. Psychiatry.

[96] van den Berg J., Verberg C., Scherpbier A., Jaarsma A., Lombarts K. (2017). Is being a medical educator a lonely business? The essence of social support. J. Med. Educ..

[97] van der Zwet J., Koelewijn-van Loon M., van den Akker M. (2009). Lonely patients in general practice: A call for revealing GPs’ emotions? A qualitative study. J. Fam. Pract..

[98] Vasileiou K., Barnett J., Barreto M., Vines J., Atkinson M., Lawson S., Wilson M. (2017). Experiences of loneliness associated with being an informal caregiver: A qualitative investigation. Front. Psychol..

[99] Vasileiou K., Barnett J., Barreto M., Vines J., Atkinson M., Long K., Bakewell L., Lawson S., Wilson M. (2019). Coping with loneliness at university: A qualitative interview study with students in the UK. J. Ment. Health Prev..

[100] Walkner T., Weare A., Tully M. (2018). “You get old. You get invisible”: Social isolation and the challenge of communicating with aging women. J. Women Aging.

[101] Warren B. (1993). Explaining social isolation through concept analysis. Arch. Psychiatr. Nurs..

[102] Winterstein T., Eisikovits Z. (2005). The experience of loneliness of battered old women. J. Women Aging.

[103] Wong A., Chau A., Fang Y., Woo J. (2017). Illuminating the psychological experience of elderly loneliness from a societal perspective: A qualitative study of alienation between older people and society. Int. J. Environ. Res. Public Health.

[104] Zumaeta J. (2019). Lonely at the top: How do senior leaders navigate the need to belong?. J. Leadersh. Org. Stud..

[105] Chile L., Black X., Neill C. (2014). Experience and expression of social isolation by inner-city high-rise residents. J. Hous. Care Support.

[106] Dong X., Chang E., Wong E., Simon M. (2011). Perception and negative effect of loneliness in a Chicago Chinese population of older adults. Arch. Gerontol. Geriatr..

[107] Finlay J., Kobayashi L. (2018). Social isolation and loneliness in later life: A parallel convergent mixed-methods case study of older adults and their residential contexts in the Minneapolis metropolitan area, USA. J. Soc. Sci. Med..

[108] Heinz M. (2018). Communicating while transgender: Apprehension, loneliness, and willingness to communicate in a Canadian sample. SAGE Open.

[109] Hinojosa R., Haun J., Hinojosa M., Rittman M. (2011). Social isolation poststroke: Relationship between race/ethnicity, depression, and functional independence. Top. Stroke Rehabil..

[110] Parigi P., Henson W. (2014). Social isolation in America. Annu. Rev. Sociol..

[111] Rew L. (2002). Relationships of sexual abuse, connectedness, and loneliness to perceived well-being in homeless youth. J. Spec. Paediatr. Nurs..

[112] Sawir E., Marginson S., Deumert A., Nyland C., Ramia G. (2008). Loneliness and international students: An Australian study. J. Stud. Int. Educ..

[113] Tahir L., Thakib M., Hamzah M., Said M., Musah M. (2017). Novice head teachers’ isolation and loneliness experiences: A mixed-methods study. J. Educ. Manag. Adm. Leadersh..

[114] de Jong Gierveld J., van Tilburg T., Dykstra P., Vangelisti A., Perlman D. (2018). New ways of theorizing and conducting research in the field of loneliness and social isolation. The Cambridge Handbook of Personal Relationships.

[115] Gedvilaite-Kordusiene M., Sagan O., Miller E. (2018). Loneliness in Lithuanian transnational families: I am happy if my children are happy?. Narratives of Loneliness: Multidisciplinary Perspectives from the 21st Century.

[116] Lake T. (1986). Loneliness–Why It Happens and How to Overcome It.

[117] Rook K., Peplau L., Goldston S. (1984). Interventions for loneliness: A review and analysis. Preventing the Harmful Consequences of Severe and Persistent Loneliness.

[118] Batsleer J., Duggan J., McNicol S., Spray S., Angel K. (2018). Loneliness Connects US: Young People Exploring and Experiencing Loneliness and Friendship. 42nd Street.

[119] Brown J. (2019). Local History Cafes: Evaluation of the initial programme. Museum Development, East Midlands.

[120] Essex R. (2010). Parental Experiences of the ‘Time Together’ Home Visiting Intervention: An Attachment Theory Perspective. Ph.D. Thesis.

[121] Huijbers K. (2018). The Advocacy Project User Focused Monitoring Evaluation Report: Loneliness and Social Isolation.

[122] Lukes-Dyer N. (2018). Social Isolation Risk among Older Adults Who Live Alone. Ph.D. Thesis.

[123] Macomber C.A. (2017). Understanding the Intersection of Loneliness and Recovery Setting in Older Cardiac Patients. Ph.D. Thesis.

[124] Moore S., Preston C. (2015). The Silver Line: Tackling Loneliness in Older People.

[125] The Mental Health Foundation (2018). An Evaluation of the Standing Together Project.

[126] Qin Y. (2012). The Effects of Loneliness on Consumers’ Digital Engagement with Social Media Ads. Ph.D. Thesis.

[127] Quinn J., Blandon C. (2014). The Power of Songs: An Evaluation of Plymouth Music Zone’s ‘Keep Singing, Keepsake’ Project.

[128] Sital-Singh P., Nicklin A., Fry B. (2018). A Place to Belong: The Role of Local Youth Organisations in Addressing Youth Loneliness.

[129] Todd C. (2017). Exploring the Role of Museums for Socially Isolated Older People. Ph.D. Thesis.

[130] Zubairi K. (2018). The Zubairi Report: The Lived Experience of Loneliness and Social Isolation in Scotland.

[131] The Red Cross (2016). Trapped in a Bubble: An Investigation into Triggers for Loneliness in the UK.

[132] Bates J., Machin A. (2015). Locality, loneliness and lifestyle: A qualitative study of factors influencing women’s health perceptions. Health Soc. Care Community.

[133] Bennett K., Victor C. (2012). ‘He wasn’t in that chair’: What loneliness means to widowed older people. Int. J. Ageing Later Life.

[134] Costello J. (2002). Grief and loneliness in older people: Case study accounts of conjugal bereavement. Qual. Ageing.

[135] Davies N., Crowe M., Whitehead L. (2016). Establishing routines to cope with the loneliness associated with widowhood: A narrative analysis. J. Psychiatr. Ment. Health Nurs..

[136] Florczak K.L., Lockie N. (2019). Losing a partner: Do continuing bonds bring solace or sorrow?. J. Death Stud..

[137] Graneheim U., Lundman B. (2010). Experiences of loneliness among the very old: The Umea 85+ project. Aging Ment. Health J..

[138] Heravi-Karimooi M., Anoosheh M., Foroughan M., Sheykhi M., Hajizadeh E. (2010). Understanding loneliness in the lived experiences of Iranian elders. Scand. J. Caring Sci..

[139] Karlsson E., Andersson K., Ahlstrom B. (2013). Loneliness despite the presence of others: Adolescents’ experiences of having a parent who becomes ill with cancer. Eur. J. Oncol. Nurs..

[140] McInnis G., White J. (2001). A phenomenological exploration of loneliness in the older adult. Arch. Psychiatr. Nurs..

[141] Muir J., McGrath L. (2018). Life lines: Loss, loneliness and expanding meshworks with an urban walk and talk group. J. Health Place.

[142] Nunkoosing K. (2013). Commentary on ‘Resisting loneliness’ dark pit: A narrative therapy approach’. Tizard Learn. Disabil. Rev..

[143] Pettigrew S., Roberts M. (2008). Addressing loneliness in later life. J. Aging Ment. Health.

[144] Riches G., Dawson P. (1996). ‘An intimate loneliness’: Evaluating the impact of a child’s death on parental self-identity and marital relationships. J. Fam. Ther..

[145] Rokach A. (1989). Loneliness: The Experience and Its Antecedents. J. Psychol..

[146] Sagan O. (2008). The loneliness of the long-anxious learner: Mental illness, narrative biography and learning to write. J. Psychodyn. Pract..

[147] Theeke L., Mallow J., Gianni C., Legg K., Glass C. (2015). The experience of older women living with loneliness and chronic conditions in Appalachia. J. Rural Ment. Health.

[148] Wijesiri H.S.M.S.K., Samarasinghe K., Edberg A.K. (2019). Loneliness among older people living in care homes in Sri Lanka. Int. J. Older People Nurs..

[149] Wiseman H. (2008). On failed intersubjectivity: Recollections of loneliness experiences in offspring of holocaust survivors. Am. J. Orthopsychiatry.

[150] Barg F., Huss-Ashmore R., Wittink M., Murray G., Bogner H., Gallo J. (2006). A mixed-methods approach to understanding loneliness and depression in older adults. J. Gerontol..

[151] Drageset J., Eide G., Dysvik E., Furnes B., Hauge S. (2015). Loneliness, loss, and social support among cognitively intact older people with cancer, living in nursing homes: A mixed-methods study. Clin. Interv. Aging.

[152] Marcille L., Cudney S., Weinert C. (2012). Loneliness as experienced by women living with chronic illness in rural areas. J. Holist. Nurs..

[153] Merz E., de Jong Gierveld J. (2016). Childhood memories, family ties, sibling support and loneliness in ever-widowed older adults: Quantitative and qualitative results. J. Ageing Soc..

[154] Corcoran R., Marshall G., Sagan O., Miller E. (2017). From lonely cities to prosocial places: How evidence-informed urban design can reduce the experience of loneliness. Narratives of Loneliness: Multidisciplinary Perspectives from the 21st Century.

[155] de Jong Gierveld J., Broese Van Groenou M.I., Bookwala J. (2016). Older couple relationships and loneliness. Couple Relationships in Mid and Late Life: Their Nature, Complexity and Role in Health and Illness.

[156] Haines S. (2018). An Evaluation of Rainbow Services Community Builder Project.

[157] Hall V. (2012). The Experience of ‘Coupled Loneliness’: A Phenomenological Investigation with Nine Women. Ph.D. Thesis.

[158] Heinrich M. (2019). “It Was Like the Titanic, with the Iceberg”: Middle-Aged (40–65yo) Men’s Lived Experiences of Loneliness within Intimate Partner Relationships (LIPRs). Ph.D. Thesis.

[159] Cherry K., Smith D. (1993). Sometimes I cry: The experience of loneliness for men with AIDS. J. Health Commun..

[160] Chung B., Olofsson J., Wong F., Rämgård M. (2020). Overcoming existential loneliness: A cross-cultural study. BMC Geriatr..

[161] Dahlberg K. (2007). The enigmatic phenomenon of loneliness. Int. J. Qual. Stud. Health Well-Being.

[162] Goldberg C. (2001). Loneliness and dread as time sense disturbances. J. Contemp. Psychother..

[163] Goossens J., Delbaere I., Beeckman D., Verhaeghe S., Van Hecke A. (2015). Communication difficulties and the experience of loneliness in patients with cancer dealing with fertility issues: A qualitative study. Oncol. Nurs. Forum.

[164] Hemberg J., Nyqvist F., Nasman M. (2018). Homeless in life loneliness experienced as existential suffering by older adults living at home: A caring science perspective. Scand. J. Caring Sci..

[165] Larsson H., Ramgard M., Bolmsjo I. (2017). Older persons’ existential loneliness, as interpreted by their significant others: An interview study. BMC Geriatr..

[166] Larsson H., Edberg A.K., Bolmsjö I., Rämgård M. (2019). Contrasts in older persons’ experiences and significant others’ perceptions of existential loneliness. Nurs. Ethics.

[167] Nilsson B. (2008). The tune of want in the loneliness melody: Loneliness experienced by people with serious mental suffering. Scand. J. Caring Sci..

[168] Nortvedt L., Lohne V., Kumar B., Hansen H. (2016). A lonely life: A qualitative study of immigrant women on long-term sick leave in Norway. Int. J. Nurs. Stud..

[169] Nystrom M. (2006). Aphasia—An existential loneliness: A study on the loss of the world of symbols. Int. J. Qual Stud. Health Well-Being.

[170] Rosedale M. (2009). Survivor loneliness of women following breast cancer. Oncol. Nurs. Forum.

[171] Sagan O. (2017). The loneliness of personality disorder: A phenomenological study. Ment. Health Soc. Incl..

[172] Sand L., Strang P. (2006). Existential loneliness in a palliative home care setting. J. Palliat. Med..

[173] Sjoberg M., Edberg A., Rasmussen B., Beck I. (2018). Being acknowledged by others and bracketing negative thoughts and feelings: Frail older peoples’ narrations of how existential loneliness is eased. Int. J. Older People Nurs..

[174] Stein J., Tuval-Mashiach R. (2015). Loneliness and isolation in life-stories of Israeli veterans of combat and captivity. Psychol. Trauma.

[175] Sundstrom M., Edberg A., Ramgard M., Blomqvist K. (2018). Encountering existential loneliness among older people: Perspectives of health care professionals. Int. J. Qual. Stud. Health Well-Being.

[176] Sundström M., Blomqvist K., Edberg A.K. (2020). Being a volunteer encountering older people’s loneliness and existential loneliness: Alleviating loneliness for others and oneself. Scand. J. Caring Sci..

[177] Kvaal K., Halding A., Kvigne K. (2014). Social provision and loneliness among older people suffering from chronic physical illness. A mixed-methods approach. Scand. J. Caring Sci..

[178] Le Roux E. (1999). Loneliness in the Therapeutic Dialogue: An Interpretation According to the Concepts of Winnicott and Heidegger. Ph.D. Thesis.

[179] Bekhet A.K., Zauszniewski J.A., Nakhla W.E. (2008). Happiness: Theoretical and empirical considerations. Nurs. Forum.

[180] Rahimzadeh S., Pour E’etemad H., Asgari A., Hojjat M. (2012). Conceptual basics of loneliness: A qualitative research. J. Evol. Psychol. Iran. Psychol..

[181] Ettema E.J., Derksen L.D., van Leeuwen E. (2010). Existential loneliness and end-of-life care: A systematic review. Theor. Med. Bioeth..

[182] Adams R.N., Mosher C.E., Winger J.G., Abonour R., Kroenke K. (2018). Cancer-related loneliness mediates the relationships between social constraints and symptoms among cancer patients. J. Behav. Med..

[183] Peplau L.A., Perlman D. (1982). Loneliness: A Sourcebook of Current Theory, Research, and Therapy.

[184] Perlman D., Peplau L.A., Gilmour R., Duck S. (1981). Toward a social psychology of loneliness. Personal Relationships: Relationships in Disorder.

[185] Moustakas C. (1961). Loneliness.

[186] ONS Exploring Loneliness in Children, Great Britain: 2018. https://www.ons.gov.uk/peoplepopulationandcommunity/wellbeing/articles/exploringlonelinessinchildrengreatbritain/2018.

[187] Victor C.R., Yang K. (2012). The prevalence of loneliness among adults: A case study of the United Kingdom. J. Psychol..

[188] Coyle C.E., Dugan E. (2012). Social isolation, loneliness and health among older adults. J. Aging Health.

[189] Karnick P.M. (2005). Feeling lonely: Theoretical perspectives. Nurs. Sci. Q..

[190] Pinel E.C., Long A.E., Murdoch E., Helm P. (2017). A prisoner of one’s own mind: Identifying and understanding existential isolation. Pers. Individ. Differ..

[191] Long C., Seburn M., Averill M., More T. (2003). Solitude experiences: Varieties, settings, and individual differences. Pers. Soc. Psychol. Bull..

