# Supplementary material for: A Conceptual Review of Loneliness in Adults: Qualitative Evidence Synthesis
Source: Int J Environ Res Public Health. 2021 Nov 2;18(21):11522. doi: 10.3390/ijerph182111522 (PMC8582800; doi:10.3390/ijerph182111522)
Supplement: Supplementary file 1 [file ijerph-18-11522-s001.zip › SupplementaryMaterial S1.MansfieldSearchStrategydocx.pdf]

**Supplementary Material S1: Search Terms and Strings**

**Electronic searches**

**Databases searched**

Scopus
Ovid
Eric
PsychInfo
CINAHL Plus
Arts and Humanities Citation Index (Web of Science)
Social Science Citation Index (Web of Science)
Science Citation Index (Web of Science)

**Search Terms and strings for each database**

**Ovid Medline**
Lonel*.mp
Social Isolation.mp
Model*.mp
Framework.mp
Concept*.mp
Theory.mp

String
(1 or 2) AND (4 or 5 or 6 or 7)

**Scopus**
String
Lonel* OR “social Isolation” AND model* OR framework OR concept OR theory

**Web of Science**
String
Lonel* OR “social Isolation” AND model* OR framework OR concept OR theory

**Eric**
String
Lonel* OR “social Isolation” AND model* OR framework OR concept OR theory

**APA PsycInfo**
String
Lonel* OR “social Isolation” AND model* OR framework OR concept OR theory
