# Supplementary material for: A Conceptual Review of Loneliness in Adults: Qualitative Evidence Synthesis
Source: Int J Environ Res Public Health. 2021 Nov 2;18(21):11522. doi: 10.3390/ijerph182111522 (PMC8582800; doi:10.3390/ijerph182111522)
Supplement: Supplementary file 1 [file ijerph-18-11522-s001.zip › SupplementaryMaterial S2.Mansfield.ExcludedStudies.pdf]

Supplementary Material S2: Table of excluded studies and reasons for exclusions		
**Authors (Year)**	**Title**	**Reason for Exclusion**
Andersson et al (2014)	Aloneness and loneliness - persons with severe mental illness and experiences of being alone.	Participants
Andrews et al (2003)	Assisting friendships, combating loneliness: users views on a befriending scheme.	Study Design
Arpin & Moyer (2019)	Transient Loneliness and the Perceived Provision and Receipt of Capitalization Support Within Event-Disclosure Interactions	Theory
Ausín et al (2019)	Confirmatory factor analysis of the Revised UCLA Loneliness Scale (UCLA LS-R) in individuals over 65	Theory
Barer (1990, MM)	Loneliness and Solitude in Late Late Life.	Study Design
Baumbusch (2004)	Unclaimed treasures: Older women's reflections on lifelong singlehood.	Exposure
Bennett (2016, Book Chapter)	Loneliness in cultural context: A look at the life-history narratives of older Southeast Asian refugee women.	Unavailable
Bergland et al (2016)	Experiences of older men living alone: A qualitative study.	Exposure
Berkman (2016, Book Chapter)	Social networks and social isolation.	Unavailable
Blake (1995)	The Social Isolation of Young Men with Quadriplegia	Exposure
Bucher et al (2019)	Together is Better: Higher Committed Relationships Increase Life Satisfaction and Reduce Loneliness	Theory
Cacioppo (2013, Book Chapter)	Perceived social isolation within personal and evolutionary timescales	Superseded by recent publication
Cacioppo & Cacioppo (2018, Book Chapter)	Loneliness in the Modern Age: An Evolutionary Theory of Loneliness (ETL)	Unavailable
Cao et al (2020)	Correlation among psychological resilience, loneliness, and internet addiction among left-behind children in China: A cross-sectional study.	Study Design
Casey & Holmes (1995)	The inner ache: An experiential perspective on loneliness	Study Design
Cattan et al (2003)	Alleviating Social Isolation and Loneliness among Older People	Unavailable
Courtney & Meyer (2020)	Self-other representation in the social brain reflects social connection	Theory
Davidson & Stayner (1997)	Loss, loneliness and the desire for love: perspectives on the social lives of people with schizophrenia.	Exposure
de Beer (2016)	Men's experience of loneliness after the loss of a partner: a description of a narrative pastoral involvement	Study Design
de Jong Gierveld (2016, Book Chapter)	Loneliness and Social Isolation	Superseded by recent publication
Dell et al (2019)	Loneliness and depressive symptoms in middle aged and older adults experiencing serious mental Illness	Theory
DiTommaso (2016, Book Chapter)	Chronic loneliness within an attachment framework: Processes and interventions.	Unavailable
Duck et al (1994)	Loneliness and the Evaluation of Relational Events	Study Design
Düzel et al (2019)	Structural brain correlates of loneliness among older adults	Theory
Elmose (2019)	Understanding loneliness and social relationships in autism: the reflections of autistic adults	Theory
Ettridge et al (2017)	Prostate cancer is far more hidden: Perceptions of stigma, social isolation and help-seeking among men with prostate cancer	Exposure
Ewertzen et al (2012)	A lonely life journey bordered with struggle: Being a sibling of an individual with psychosis	Exposure
Feng et al (2019)	Connectome-based individualized prediction of loneliness	Theory
Fokkema & Knipscheer (2007)	Escape loneliness by going digital: A quantitative and qualitative evaluation of a Dutch experiment in using ECT to overcome loneliness among older adults.	Study Design

Forsbrey et al (2005)	Social isolation among caregivers of court-involved youths: A qualitative investigation	Exposure
Fuentes et al (2014)	Promoting Self-Reflection of Social Isolation Through Persuasive Mobile Technologies: The Case of Mother Caregivers of Children With Cancer	Exposure
Gannon-Leary et al (2011)	The loneliness of the long distance researcher	Exposure
Gedvilaitė-Kordušienė *(2018, Book Chapter)*	Loneliness in Lithuanian transnational families: â€˜I am happy if my children are happyâ€™?	Duplicate
Gill et al (2016)	Loneliness during inpatient rehabilitation: Results of a qualitative study.	Exposure
Greene (1978)	Aspects of loneliness in the therapeutic situation.	Study Design
Hauge & Kirkevold (2010)	Older Norwegians' understanding of loneliness	Study Design
Hawkley (2011, Book Chapter)	Perceived social isolation: Social threat vigilance and its implications for health.	Study Design
Hill (2017, Book Chapter)	Loneliness as an occupational hazard: Academic identities and the neoliberal work ethic	Unavailable
Helm (2020)	Existential isolation, loneliness, and attachment in young adults	Theory
Hussain et al (2018, MM)	Caregiving, employment and social isolation: Challenges for rural carers in Australia	Exposure
Jefferies & Clifford (2011)	Aloneness: the lived experience of women with cancer of the vulva.	Exposure
Jerusalem et al (1996)	Social bonding and loneliness after network disruption: A longitudinal study of East German refugees	Study Design
Johansson & Andreasson (2017)	The web of loneliness: A netnographic study of narratives of being alone in an online context	Not English Language
Kauten et al (2017, Book Chapter)	Loneliness and suicide	Unavailable
Koller & Gosden (1984, MM)	On living alone, social isolation and psychological disorder	Exposure
Laryea & Gien (1993)	The Impact of HIV-Positive Diagnosis on the Individual, Part 1: Stigma, Rejection, and Loneliness	Exposure
LeGrand et al (2014)	If you build it will they come? Addressing social isolation within a technology-based HIV intervention for young black men who have sex with men	Exposure
Levy (2001)	All the Lonely People...Where Do They All Belong?	Exposure
Long et al (2001)	Fear and social isolation as consequences of tuberculosis in Vietnam: a gender analysis.	Exposure
Longman et al (2013)	The role of social isolation in frequent and/or avoidable hospitalisation: Rural community-based service providers' perspectives	Exposure
Lovanio (2015, Book Chapter)	Social isolation	Study Design
Machielse (2005, Book Chapter)	Theories on social contacts and social isolation	Unavailable
Machielse (2015)	The Heterogeneity of Socially Isolated Older Adults: A Social Isolation Typology	Exposure

MacKinlay (2002)	Ageing and isolation: Is the issue social isolation or is it lack of meaning in life?	Exposure
McHugh power et al (2019)	Loneliness and social engagement in older adults: A bivariate dual change score analysis.	Study Design
Maeda (2017)	Self-Efficacy Reduces Impediments to Classroom Discussion for International Students: Fear, Embarrassment, Social Isolation, Judgment, and Discrimination	Exposure
Martina (2018)	Change and stability in loneliness and friendship after an intervention for older women.	Study Design
Martin-Maria et al (2020)	Effects of transient and chronic loneliness on major depression in older adults: A longitudinal study	Theory
Massimo & Caprino, (2015, Book Chapter)	Psychosocial effects of loneliness on the parents of health migrants: Stress and anger within	Exposure
Mellado et al (2016)	Social isolation in women with endometriosis and chronic pelvic pain	Exposure
McGlone et al (2020)	Are young adults with long-standing illness or disability at increased risk of loneliness? Evidence from the UK Longitudinal Household Study.	Study Design
Mijuskovic (2015, Book Chapter)	Cognitive and motivational roots of universal loneliness	Unavailable
Mikulincer & Segal (1990)	A Multidimensional Analysis of the Experience of Loneliness	Exposure
Miles (2011)	Silent endurance and profound loneliness: Socioemotional suffering in African Americans living with HIV in the rural South.	Exposure
Moyle et al (2011)	Dementia and loneliness: an Australian perspective.	Exposure
Mund et al (2020a)	Loneliness is associated with the subjective evaluation of but not daily dynamics in partner relationships	Theory
Murrock (2016)	Depression, Social Isolation, and the Lived Experience of Dancing in Disadvantaged Adults.	Exposure
Neto (2005)	Sex differences in Portuguese Lonely Hearts advertisements	Exposure
Nzabona et al (2016, MM)	Loneliness among older persons in Uganda: Examining social, economic and demographic risk factors	Exposure
Oliffe et al (2018)	Unpacking Social Isolation in Men’s Suicidality	Exposure
Ozaki et al (2016)	Social isolation and cancer management after the 2011 triple disaster in Fukushima, Japan: A case report of breast cancer with patient and provider delay	Exposure
Pals (2006)	The cult of the dead and leisure: Escaping loneliness	Exposure
Patron (2015, Book Chapter)	Students' loneliness during cross-cultural adjustments.	Unavailable
Pitkala et al (2014, Book Chapter)	Group dynamics in older people's closed groups: Findings from Finnish psychosocial group rehabilitation for lonely older people	Unavailable
Power et al (2017)	Exploring the meaning of loneliness among socially isolated older adults in rural Ireland	Duplicate (late)
Power et al (2019)	Comparisons of the discrepancy between loneliness and social isolation across Ireland and Sweden: findings from TILDA and SNAC-K.	Study Design
Riches & Dawson (1996)	'An intimate loneliness': Evaluating the impact of a child's death on parental self-identity and marital relationships	Exposure
Riley (2014)	The lonely congressmen: Gender and politics in early Washington, D.C.	Study Design
Rokach (1996)	The subjectivity of loneliness and coping with it	Study Design
Rokach & Sha'ked (2013, Book Chapter)	Together and lonely: Loneliness in intimate relationships - causes and coping	Study Design
Rook (1988, Book Chapter)	Toward a more differentiated view of loneliness.	Unavailable

Rubin (2009, Book Chapter)	Loneliness	Study Design
Rudolf (2017, Book Chapter)	Psychology of Loneliness: New Research	Study Design
Sadler (1978)	Dimensions in the Problem of Loneliness: A Phenomenological Approach in Social Psychology	Study Design
Sagan (2017, Book Chapter)	Narratives of loneliness and mental ill health in a time of neoliberalism	Unavailable
Seefeldt (2016, Book Chapter)	Abandoned families: Social isolation in the twenty-first century	Study Design
Segrin (1998, Book Chapter)	Interpersonal communication problems associated with depression and loneliness.	Study Design
Shiovitz-Ezra (2013, Book Chapter)	Confidant networks and loneliness	Study Design
Slettebö (2008)	Safe, but lonely: Living in a nursing home.	Exposure
Smith (2012)	Toward a better understanding of loneliness in community-dwelling older adults	Duplicate
Smith (2012a)	Portraits of loneliness: emerging themes among community-dwelling older adults	Duplicate
Squires (2015)	To a Deeper Understanding of Loneliness amongst Older Irish Adults.	Study Design
Stacciarini et al (2015, MM)	Rural Latinos Mental Wellbeing: A Mixed-Methods Pilot Study of Family, Environment and Social Isolation Factors	Exposure
Stein (2017, Book Chapter)	The veteran's loneliness: Emergence, facets, and implications for intervention	Superseded by recent publication
Stein & Solomon (2018, Book Chapter)	The lonely side of war's aftermath: Traumatization and isolation among veterans	Unavailable
Stewart (2007, MM)	'Left Out': Perspectives on social exclusion and social isolation in low-income populations.	Exposure
Tarbi & Meghani (2019)	Existential Experience in Adults with Advanced Cancer: A Concept Analysis	Theory
Traeen & Sorensen (2000)	Breaking the speed of the sound of loneliness: Sexual partner change and the fear of intimacy	Exposure
Uotila et al (2010)	Lonely older people as a problem in society - construction in Finnish media	Study Design
Van Der Geest (2004)	"They don't come to listen": The experience of loneliness among older people in Kwahu, Ghana	Exposure
van Ravesteijn et al (2008)	GPs' experiences with loneliness	Duplicate
Victor (2015, Book Chapter)	Loneliness and later life: Concepts, prevalence, and consequences.	Unavailable
Victor & Sullivan (2015, Book Chapter)	Loneliness and isolation	Superseded by recent publication
Victor et al (2015, MM)	Dancing with loneliness in later life: A pilot study mapping seasonal variations	Study Design
Wells (1990)	The "Terrible Loneliness": Loneliness and worry in settler women's memoirs from East and South-Central Africa, 1890-1939	Study Design
Wilson (2018, MM)	Is it love or loneliness? Exploring the impact of everyday digital technology use on the wellbeing of older adults	Exposure
Wright (2009, Book Chapter)	In a lonely place: The experience of loneliness in the workplace	Unavailable
Yannakopoulos (2010)	Cultural meanings of loneliness: Kinship, sexuality and (homo)sexual identity in contemporary Greece	Exposure

Yodovich & Lahad (2018)	I don't think this woman had anyone in her life: Loneliness and singlehood in Six Feet Under	Study Design
Yue et al (2011, Book Chapter)	Being lonely in a crowd: Population density contributes to perceived loneliness in China	Unavailable
Zamir et al (2018)	Video-calls to reduce loneliness and social isolation within care environments for older people: An implementation study using collaborative action research	Exposure
