# Supplementary material for: A Conceptual Review of Loneliness in Adults: Qualitative Evidence Synthesis
Source: Int J Environ Res Public Health. 2021 Nov 2;18(21):11522. doi: 10.3390/ijerph182111522 (PMC8582800; doi:10.3390/ijerph182111522)
Supplement: Supplementary file 1 [file ijerph-18-11522-s001.zip › SupplementaryMaterial S3.Mansfield.IncludedStudies.pdf]

Supplementary Material S3: Characteristics of included studies (published and unpublished literature)

Authors, date & location	Objectives	Description of study and study Participants	Study Design and Analysis	Definition or conceptualisation of loneliness	Themes or domains of loneliness	Conclusions
[24]Adams et al (2016, USA)	To identify situations and thoughts that may precipitate or protect against loneliness experienced by patients with cancer.	Purposive sample N=15 patients with cancer currently undergoing treatment. Balanced for gender and age. First author conducted individual semi-structured interviews. Participants given definition of loneliness and asked about experiences of it / situations and thoughts that protect against it. All participants 18+, English speaking.	Semi-structured interviews transcribed verbatim. Iterative sampling and coding process using two coders. Saturation by agreement. ATLAS.ti used for theoretical thematic analysis (Braun & Clarke, 2006).<br><br>Analysis identified situations that lead to loneliness, and precipitating & protective thoughts.	Participants given definition to respond to: "Loneliness means feeling isolated or disconnected from others around you. It isn't about how much time you spend with others or how many friends you have, but more about how connected you feel with others overall." (p157)<br><br>Loneliness theory guided analysis (Cacioppo et al., 2006; Cacioppo & Hawkley, 2009; Peplau & Perlman, 1982).	Study explored precipitants of loneliness. Findings were consistent with suggestion that perception of social isolation, rather than actual isolation, underlies loneliness (Cacioppo & Hawkley, 2009; Peplau & Perlman, 1982)<br><br>Also linked to cognitive theoretical framework for depression - interpretations of environment affect mood (Beck 2011).	Developed conceptual model of patients' loneliness.<br><br>Negative social cognitions are linked with greater loneliness (Cacioppo & Hawkley 2009).<br><br>Perceptions of social situations, rather than situations themselves had greatest impact. Patients with negative explanations for others' behaviour and negative social expectations tended to endorse loneliness.<br><br>Future studies could explore whether cognitions identified predict loneliness. If so, interventions could be developed to address them.
[25] Anaker et al (2018, Sweden)	The aim of this study was to explore patients' experiences of the physical environment at a newly built stroke unit.	Interview with patients at a stroke unit in Sweden who were asked to describe their experience of the physical environment of their room and of the stroke unit as a whole.<br><br>Patients (N =16) participated in semi-	Qualitative design with an inductive analytic approach. Interviews transcribed verbatim and analysed using content analysis. Inductive content analysis performed using methods of Elo	Effective stroke unit design (which encourages and provides space for communal activities) can contribute to alleviating loneliness	Patients reported being lonely in single rooms. This issue mentioned repeatedly by majority of patients interviewed. Patients said that being cared for in a single room was lonely and empty and that they had no one to talk with during the day.	To reduce loneliness, stroke units should be designed to enable patients to visit communal areas where they can experience social interactions and have access to a stimulating environment that includes, e.g. books, games, and computers.

		structured individual interviews. (F, 7; M, 9). Two of the participants were wheelchair users.	and Kyngäs (2008) and Krippendorff (2004).		Most felt single-room design as both facilitator of privacy and barrier to reducing loneliness. Patients expressed desire for both community and privacy in design of the environment.	
[26] Apostolopoulos et al (2016, USA)	To examine the social isolation and inherent difficulties involved with long haul trucking, and the subsequent mental health consequences.	The authors provide an initial examination of long-haul truckers' mental health risks and the multifaceted ramifications, including social isolation.<br><br>N=60 long-haul truckers.	Ethnography with qualitative interviews.<br><br>Analysis included conceptual mapping to graphically explore relationships among themes.	U.S. long-haul truckers' chronic and immediate work strains create a chronic state of transience and social isolation and are linked with weak professional and community support systems and substance misuse — influencing their mental health, particularly during traumatic life events. These strains are exacerbated by perpetual mobility and chronic isolation.	Social isolation caused by truckers' jobs emerged as a recurring theme in all interviews. The prolonged periods of loneliness have significant impacts on drivers while also having a detrimental effect on family dynamics, as drivers miss out not only on important events but more importantly on the day-today interactions vital for family bonding. Drivers expressed the huge strain placed on their relationships as a result of these prolonged absences.	Findings support the statement that social isolation and inherent difficulty of establishing and maintaining strong social ties while on the road exact a heavy toll on drivers' mental health. Truckers struggle with loneliness, particularly related to the lack of reliable support systems. They are overstressed as a result of their work pressures, excess physical and mental overload, the need to maintain constant vigilance, and associated sleep problems.<br><br>Socialising at a truck stop may help a trucker feel less lonely in the moment, but he may still be far away from family members who provide meaningful emotional, moral, and/or social support. This distance exacerbates chronic feelings of isolation, contributing to depression.
[27] Ballin & Balandin (2009, Australia)	To explore loneliness in the communication	Findings presented from larger qualitative study on the loneliness experiences	Qualitative interviews. Initial analysis to identify the overarching themes and recurrent	Deficits in a person's social relationships lead to loneliness. Loneliness differs from social	All participants agreed that the themes of communication and social networks are most important when considering	Results suggest communication and social networks should be considered when discussing loneliness in this population.

	and social networks of older people with cerebral palsy.	of older people with cerebral palsy.<br><br>N=7 older adults with cerebral palsy, M, 4; F, 3 participated in this study. 4 participants were considered to have a severe physical disability, requiring more than 4 hours of daily assistance.	patterns or topics. Topics were coded and grouped under the overarching themes by both authors using consensus (Luborsky, 1994).	isolation; subjective experience – a person may be alone without being lonely or may be lonely in a crowd. Deficits in social relationships can result from lack of involvement in a satisfying social network, absence or loss of meaningful friendships.	loneliness.<br><br>Impact of reduced opportunities for quality communication was an important consideration in any discussion of loneliness in older adults with cerebral palsy.<br><br>Having sufficient social time, using the internet to communicate, having time with friendly staff, friends, family and a partner were all important for alleviating loneliness.	Results demonstrate need to provide support and training in communication to older people with cerebral palsy who experience communication difficulty, and to their communication partners. There may be a need for policy development to assist older adults with cerebral palsy to develop and maintain social networks and form rewarding relationships.
[28] Bantry-White et al (2018, Ireland)	To explore constructions of rural community and how these are embedded within a befriending programme designed to tackle social isolation and loneliness.	Examined how representations of rural community in Ireland influenced the focus, relationships and activities within a befriending intervention designed to tackle social isolation and loneliness.<br><br>N=22 participants, 8 befriended, 11 volunteer befrienders and 3 community workers.<br><br>Participants in receipt of befriending: F, 6; M, 2, aged 58–92 years (median 76.88 years).	Qualitative case study using individual interviews and focus groups.<br><br>Draws upon Taylor's (2004) theorizing of the social imaginary, defined as a common set of beliefs and/or expectations shared among a group of people.	Social isolation and loneliness cannot be singularly viewed as symptomatic of the demise of the social self in later life but as part of a symbolic construction of community.	Findings demonstrated that social isolation and loneliness were understood with reference to a social imaginary of community that idealised former times. Key strands within this construction were place, sameness and solidarity. Social isolation and loneliness were the normative antithesis of community and were perceived to arise from an erosion of traditional community. Self-identifying or being identified as lonely or isolated reflected perceptions of the fit between the person and the idealised community, namely a sense of belonging and engagement in shared activity, as well as a wider discourse about	Proposes a need to consider the role played by understandings of community in shaping context-sensitive interventions to counter social isolation and loneliness in later life.<br><br>With a well-established discourse of ageing-in-place in Western social policies, greater interpretive attention needs to be paid to social networks and relationships, both real and normative, that shape older people's experience of place.

[29] Barbosa Neves, Sanders & Kokanović (2019, Australia)To explore how older people (aged 65+) living in care homes experience, articulate, and respond to social isolation and loneliness.Participant observation and semi-structured interviews, in two stages.

Stage 1 included six months conducting 101 hours of participant observation in two residential care homes (the Bluebell and Rosewood), during mealtimes, in-house programme activities, holiday celebrations, and while participants were engaged in informal activities such as frequenting communal areas.

					contemporary risks within rural communities.	
4. sickness: seen as ill emotional and cognitive health, "the most frightening disease in the world", and you can change by framing things differently. A comprehensive approach to loneliness must study the relationship between agentic and structural dimensions. The findings illustrated this interaction and the importance of contexts: loneliness and social isolation are not merely an individual trouble, nor are they just a social issue; they are both. Loneliness and social isolation are thus multidimensional: such as relating to oldering as a structural dimension of one	To explore experiences of loneliness in a group of older people in order to consider community level approaches to prevent and reduce feelings of loneliness.	Group of local older people (aged 50+) were recruited as community researchers. A core group of eight individuals formed. N=14 people interviewed, aged between 52 and 88 (mean age .72 years).<br><br>Researchers tried to answer the question: How do older people experience loneliness in the Greater Bedminster area and what activities and/or services could be developed in order to reduce/prevent feelings of loneliness?	Qualitative interviews.<br>Co-production with community research group.<br>During each session group divided into pairs and each pair actively read an interview transcript, making notes and coding data. Each pair discussed their codes and identified emergent themes.	Loneliness is complex and multifaceted. It relates to interpersonal social relationships (both in terms of quality and quantity), specific life events, social structures, as well as an individual's social environment (Joping, 2015). Determinants of loneliness are a combination of, and interplay between, internal and external factors. An individual's subjective experience and perception of their social world is key to how they experience loneliness.	Across interviews older people discussed how loneliness had impacted on their own life and the lives of those around them. Some reflected on their own concerns for the future and the plans they might put in place so they were not lonely. Others discussed reconceptualising the meaning of "older" for themselves and talked of a desire to challenge what this meant.<br>Three key themes: connecting and disconnecting, points of vulnerability and older identity. Social connections were important to participants and many felt disconnected at times.	Findings highlight the importance of feeling socially connected to community through older age.<br><br>Evidence suggests that people without meaningful social connections are at risk for loneliness (Masi et al., 2011). Social attitudes about age and individual conceptions of what being older meant impacted upon how older people themselves experienced loneliness. Loneliness is not simply a result of losing social connections but also relates to how people view themselves and how they feel older people are positioned in society.<br><br>Relationship between social connections and loneliness can be reversed; losing social connections may be a result of loneliness; evidence suggests that when people feel lonely their social interactions are likely to change as they think and act differently and their perceptions of the social environment alter (Cacioppo and Hawkley, 2009).

Four-long semi-structured interviews were conducted with residents experiencing or at risk of loneliness and social isolation.

The interviewees (n = 22) (F 16, M 6) were aged 65+ to 90 years. Out of these, (n = 2) single; (n = 2) married; (n = 2) divorced; and (n =16) widowed.

A multi-method qualitative approach.

Field notes were digitised verbatim. Interviews were digitally recorded and transcribed verbatim. Thematic analysis was conducted to uncover themes and patterns describing loneliness and social isolation (Fereday & Muir-Cochrane, 2006). Themes were searched within (individual) and across (collective) cases. Following Braun and Clarke's 6 steps (2006), the authors: 1) read transcripts and field notes, observing initial ideas, 2) generate preliminary codes, 3) combine codes into themes, 4) revise themes in relation to codes, extracts, quotes etc producing a thematic diagram, 5) refine the themes, 6) produce a report containing exemplar cases.

The data was coded independently, then collectively to reach consensus, both deductively and inductively.

Loneliness has been conceptualised into four categories:

1. *relational*
2. *oldering*
3. *personal troubles*
4. *sickness*

From these, participants felt that loneliness was a choice whereas social isolation was imposed and beyond their control. Hence, the two phenomena are conceptually distinct.

A distinction is made between structural (e.g., living settings, norms, etc.) and agentic (e.g., individual's actions, choices) dimensions of loneliness. For instance, the use of Mills (1959) 'personal troubles' emphasised how an agentic understanding of loneliness also draws on and shapes structural issues.

Four themes of loneliness.

1. *Loneliness as Relational*: loneliness is referred to as a loss of close spouse, family ties, quality of relationships. Older adults had less access to meaningful social interaction, this included the norms, functions and expectations around social interactions. "when we would occupy a seat, a resident would quickly inform us of whom that place 'belonged' to.

2. *Oldering*: age-related circumstances, such as reminiscing about past, aged-care dependency, loss of mobility, frailty, institutionalisation, feelings of social abandonment, [illegible]

hand, but resting on an agentic dimension, as individual fault (e.g., for not making friends). Connected to the agentic dimension is seeing loneliness and social isolation as private matters, subjective, and hard to talk about (Schoenmakers & Tindemans, 2017; Stanley et al., 2010). Perceiving these phenomena as individual fault or responsibility explains the stigma, pain, shame, and guilt reported by participants (McHugh et al., 2017; McInnis & White, 2001). It also explains why, despite the structural understandings of the phenomena, most participants still saw it as their responsibility to address it.						

[30] Barke (2017, England)						
[31] Bess & Doykos (2014, USA)	Examines the efforts of one neighborhood-based human service organisation to counter the effects of social isolation through a place-based parent education initiative,	Investigates the experiences and perceptions of parents who participated in and graduated from Tied Together, a place-based parent education initiative with a focus on their experience of it as a setting for making social and institutional connections.<br><br>69 program graduates (F, 58; and M, 11). 51 lived in a public housing community, 15 lived in other neighborhoods or were homeless, and three did not report where they lived. Participant demographics reflect those of the local community: 85% African American, 87% women, 81% single, 84% unemployed. Participants ranged in age from 15 to 57 years.	Qualitative case study.<br><br>Authors examine graduate narratives related to an increased sense of connection and reduced isolation. They subsequently explore the range of experience related to specific nature of connections made through the program. There is also a focus on the contextual aspects of the program that may have facilitated the development and maintenance of new connections or impeded them. Attention is paid to the role of "place" in building and sustaining—and at times preventing—connections among participants.	Conceptualises connections as being formed through participation across multiple settings or contexts that make up a person's microsystem (Bronfenbrenner, 1979). These include family, school, work, neighborhood, and faith community. Social support is conceived as a resource generated within specific contexts— through both geographic communities and relational communities—flowing dynamically through the network of diverse relations (Wellman & Gulia, 1997).	Expanding network connections: While almost all participants reported a greater sense of connection to others in the program, the narratives reflected the range of experiences related to specific and sustained new connections. Of 69 graduates interviewed, only 14 did not report making new connections as a result of participation.<br><br>Connections with Tied Together staff and MOBC: Graduates attributed much success of program to staff creating a supportive environment. Majority mentioned generally how Tied Together provided support, 34 detailed specific connections with staff and support received. A repeated theme was the sense that there was someone from Tied Together who could be relied upon.	Place-based programs can provide an initial structure and ongoing context required. However, to build sustainable networks of support and communities of practice, structured opportunities for connection must extend beyond the duration of any single program. Stand-alone programs may ameliorate relational deficits, but are limited in their potential to address institutional barriers of poverty that contribute to "relational anemia" (p282).<br><br>Without access to sustainable opportunity structures providing ongoing life contexts for connection, it is less likely that residents will develop and maintain strong supportive networks.
[32] Bower et al (2017, Australia)	To explore how participants understood and constructed their social networks,	In-depth interviews were used to get a nuanced understanding of how social networks and isolation are experienced and understood by	Qualitative. Semi-structured interviews<br><br>Thematic analysis informed by Braun and Clarke (2006), focusing	Loneliness was described by participants and defined as social dissatisfaction linked to the discrepancy between the relationships they	Three themes emerged in relation to participants' social networks: (1) participant's experiences of stigma-driven rejection by non-homeless; (2) how the loss of highly valued	Participants constructed their social networks as being both constrained and enabled by marginalisation. They experienced rejection from the non-homeless: the loss of

	including experiences of social isolation and loneliness, within the context of their lives before, during, and where applicable, after homelessness.	individuals experiencing homelessness.<br><br>N=16 adults, aged 22–70, identifying as M, 6; F, 7; Intersex, 1 and transgender women, 2, who were currently homeless (N = 11) or previously homeless (<5 years) and living in public housing (N = 5).	on participant's meaning making and experiences.	have and those they desire (Peplau & Perlman, 1982). Loneliness can occur when this discrepancy leads to aversive emotions and distress (Peplau & Perlman, 1982), a phenomenon several participants described.	relationships triggered loneliness and isolation; (3) shallow, precarious relationships within homeless community.<br><br>Many:<br>• described how interactions were framed by stigma, with experiences described as rejection, based on their homelessness and other identities, e.g. gender or ethnicity. Experiencing rejection constrained the way they positioned themselves in relation to others and their ability to connect socially<br>• framed social networks around the absence or loss of valued network members e.g. family, friends, intimate partners.<br>• described being unable to "fit in" within the homeless community. Others lost intimate partners prior to homelessness, being unable to find replacement relationships due to circumstances of homeless situation and socioeconomic backgrounds.<br>were in frequent contact with other homeless people – relationships often central to their social lives.	critical network members, including rejection from family and a lack of companionship, and low quality and precarious relationships within the homeless community. These accounts were best conceptualised through loneliness theory.<br><br>Participant's accounts signal that the homeless are likely to continue feeling isolated/lonely if mainstream attitudes towards homelessness remain stigmatising and discriminatory.

[33] Breck et al (2018, USA)	To address social isolation amongst older adults by implementing a reverse mentoring programme.	Reverse mentoring examined within an intergenerational program serving older adults and using the knowledge and skills of young adults who mentor older adult participants. Data collected through young adult mentor logs and through post-surveys collected from participants.<br><br>N=87 older adults, 37 of which completed at least three sessions with the program (for a total of 243 sessions with young adult mentors). 29 completed a post-survey (Mage = 73.45, SD = 5.98).	Mixed methods with qualitative interviews and open-ended survey questions.<br><br>Analysis used a phenomenological approach (Patton, 2015), meaning the researchers analysed the data to understand older adults' and young adult mentors' perceptions of their experience in the program.	Isolation defined using the AARP Foundation, (2012, pp. 11–12) definition: Isolation is the experience of diminished social connectedness stemming from a process whereby the impact of risk factors outweighs the impact of any existing protective factors. A lack of social connectedness is measured by the quality, type, frequency, and emotional satisfaction of social ties. Social isolation can impact health and quality of life, measured by an individual's physical, social, and psychological health; ability and motivation to access adequate support for themselves; and the quality of the environment and community in which they live.	Three themes related to social connection: (1) an increased sense of self-efficacy for older adults as they build confidence in technological use, and for young adults as they develop leadership skills through mentoring, (2) the breaking down of age-related stereotypes, and (3) intergenerational engagement and connection.	Reverse mentoring can be used in various settings to decrease the social isolation of older adults by developing intergenerational connections and increasing older adult usage of technology.
[34] Canham (2015, USA)	To examine the subjective experiences of loneliness and social isolation among women older than 65,	Interviews with N=7 participants, discussing aspects of social isolation or loneliness. Part of a larger study.	Qualitative phenomenological design.<br><br>Codes for social isolation and loneliness emerged from	Loneliness is conceptualised as an individual's subjective evaluation of feeling without companionship, isolated or not belonging. Loneliness is	Three themes emerged:<br>1. Dislike being alone' (being alone and disliking being alone reported by Anne and Lily)<br>2. 'Loneliness and isolation' (feelings of loneliness	Social isolation and loneliness are negative aspects of the lived experience for older benzodiazepine-using women. Loss of companions and transportation is important to this experience. Being isolated

	living the U.S.A. who are using benzodiazepine.	All participants female, older than 65 years. English speaking and self-identifying as using a benzodiazepine ≥5 days/week over previous 3 months to treat a sleep or anxiety problem. Ages ranged from 65 to 86 years. 4 widows, 2 divorcees, 1 never married.	individual transcripts and were considered at group level. This study reports on the theme of social isolation and loneliness and related terms, which emerged following comparison across cases and reflection on participant's subjective meanings.	distinct from social isolation, which is an objective measure of the size and diversity of one's social network and frequency of social interaction.	reported by Eva, Mardie and Christine<br>3. 'Social isolation causes negative feelings' (Christine and Deborah reported being alone caused depression).	can cause depression, fear and insecurity. Future research should consider the role psychotropic medications have in coping with social isolation and loneliness among older adults. Clinicians should be aware of social isolation and loneliness in late life and discuss non-pharmacologic treatment options with ageing patients.
[35] Cela & Fokkema (2017, Italy)	To explore the lived experiences and perceived causes of loneliness amongst older migrants in Italy.	In-depth interviews were conducted between January 2013 and January 2014.<br><br>N=34 migrants living and working in Italy (aged 50+), 19 Albanians and 15 Moroccan. Socially heterogeneous group of participants. Mean age 64 for Albanians, 59 for Moroccans.<br><br>Albanians: F, 10; M, 9.<br>Moroccans: F, 4; M, 11.	Qualitative interviews, 2-3 hours each. Thematic analysis.	Perlman and Peplau (1981, p. 38) definition: "the unpleasant experience that occurs when a person's network of social relations is deficient in some important way, either quantitatively or qualitatively".<br><br>Also Jong Gierveld (1984) definition of loneliness "subjective negative assessment of one's relationships" due to a mismatch between one's actual and desired quality and number of social relations.	• Inter-individual, intra-individual and societal causes of loneliness are discussed. Widowhood is instanced as a particular case of the first of these causes.	"A general pattern emerging from our analysis of the data is that a deficit of social relationships beyond the family setting and in particular the lack of meaningful relationships with co-ethnic peers in the host country seems to generate feelings of loneliness among the participants", p. 1210.
[36] Cloutier-Fisher et al (2011, Canada)	To investigate older adults' social isolation experienced	Interviews with older adults identified as being at risk of social isolation on the basis of the self-reported size of their social	Mixed methods descriptive design.<br><br>Analysis used principles of grounded	Conceptualisation ignores the contributions of a host of other factors, including life experiences, family	Three main themes:<br>1. small social support networks reflect complex patterns of limited socialisation over life course;	There is a need for a more nuanced articulation of the construct of social isolation. Study was based on premise that objective measures of social

	within small social networks	networks. Participants' experiences discussed in the context of significant life course transitions.<br><br>N=28 participants ranged in age from 69 to 92 years with an average age of 80. M, 12; and F, 16.	theory (Strauss & Corbin, 1990). Coded statements grouped into themes and themes into categories on basis of shared meaning (Spencer, Ritchie, & O'Connor, 2005).	dynamics, and long-term patterns of socialisation.	2. meaning of kin and non-kin ties in small social networks are contingent on factors related to life histories of participants;<br>small social networks can protect older adults from social isolation and loneliness, particularly when involving peripheral social ties, e.g. church, club, community organisation.	isolation need to be paired with considerations of subjective dimensions of the phenomenon.<br><br>Findings suggest complex relationships between small social networks, social isolation and loneliness. Several participants actively sought solitude or balanced periods of being alone with opportunities for social interaction. Many described themselves as "loners" or "shy" and indicated a lifelong preference for being on their own and for solitary activities.
[37] Cohen-Mansfield & Eisner (2019, Israel)	To identify and describe meanings of loneliness among older persons, relationships they are seeking in order to alleviate it, and barriers in developing social connections.	Older people recruited through previous study (Cohen-Mansfield et al., 2018). Inclusion criteria were (1) age 65 and above; (2) feeling lonely based on degree and frequency of loneliness; not participating in social activities but expressing desire to have additional company; (3) not having dementia as determined by cognitive function; (4) not having depression as screened by the Geriatric Depression Scale (GDS). Included people with low mood.	Qualitative, Semi-structured interviews<br><br>Thematic Analysis guided by realist approach.<br><br>Used Braun and Clarke (2006) stepped approach (familiarisation, preliminary coding, coding refinement, data extraction, interpretation)	Loneliness defined and experienced as a negative emotional response to absence of company/companionship without adequate alternative, or in relation to a past state that was different (less lonely).<br><br>A complex, multidimensional and nuanced conceptualisation catching individual and institutional/structural influences.	<u>Experience of loneliness</u><br>Feelings – lack of companionship, sense of abandonment, timing (acute when comparing to less lonely times e.g. when partner alive or family around), handling loneliness (seeking contact)<br><u>External reasons</u><br>Death/loss, fear of being a burden, globalisation (people migrating), broken families (not communicating or in contact), loss/lack of activity<br><u>Addressing loneliness – relationships</u><br>Companionship, sociability, regular and in right place, renewing family, close friendship, finding spouse /	Participants' narratives reveal more complex, multi-dimensional, and nuanced concept of loneliness.<br>Individual and institutional factors shape experiences of loneliness.<br>Depth of suffering experienced by lonely older persons and the diversity of those experiences. Public policy, social centers, public media, and social media need to address complex issues of loneliness in older people.<br>.

		N=35, 66-92 years, mean age 75.9 years, F (83%) and M (17%), Israeli (39%), European/Russian (36%), NAfrica/Middle East (15%)			intimacy, people like you - compatibility (gender, SES, culture)<br><u>Barriers</u><br>Emotions, shame/pride, hiding loneliness, fear of rejection, depression/anxiety, lack of competency (technical) decline (physical, cognitive), personality (shyness/ introvert), ageism and ableism (of old people themselves)	
[38] Cross (2016, Canada)	To analyse discourses relevant to older fraud victims, as articulated in volunteer accounts.	Interviews with 21 (F, 12; M, 9) Canadian volunteers (also seniors) who provided telephone support to the fraud victims (all seniors). Average age 74 years, range 60-91 years.<br><br>The volunteers operated in the Senior Support Unit of the Canadian Anti-Fraud Centre (North Bay, Ontario).	Analysis of interviews identifying discourse.<br><br>Coding of transcriptions, identification of emergent themes.	Loneliness conceptualised as a condition of vulnerability for many elderly people living alone.	Many volunteers believed older persons became victims of fraud as a result of loneliness and isolation. This supports the element of social vulnerability within this discourse (Holtfreter, Reisig and Blomberg et al. 2006: 767). Loneliness was perceived to operate: (1) as the initial motivation for older persons to get on the Internet and start communicating; (2) the means through which offenders could establish a relationship with the victim.<br><br>Initial motivation illustrated in:<br>• "Well, a lot of it is loneliness for the seniors, a lot of it is that" (Interview 4).<br>• "They're lonely. You've got seniors living alone, and now we have the Internet, and they're on there … They're	Volunteers perceive fraud to occur out of loneliness and isolation of the victim, and actively resist victim blaming narratives towards these individuals.

					lonely. That is to me the number one, and they want a companion, so they meet and talk online" (Interview 18). "The romance scam is another issue where people are just lonely and somebody's convinced them that you've met your match' (Interview 7)", p. 65.	
[39] Esposito (2015, Italy)	To investigate the health and well-being of female inmates in Italian prisons recognising the important issue of the loneliness and seclusion experienced by inmates.	N=37 semi-structured interviews with women prisoners in prisons in 3 Italian regions – 32 Italians, two Africans, one Albanian, one Spanish, and one Romanian. Average age is 42.6, and 33 are mothers.	Qualitative study<br><br>CAQDAS done with software Atlas.ti, grounded analysis method to generate categories via the generation of Code Families.	Loneliness defined as an unpleasant experience that occurs when a person's network of social relations is deficient in some way.<br><br>Loneliness can be mild and fleeting but also a persisting, distressing experience. Loneliness is linked to a lack of interpersonal relations.<br><br>Weiss: loneliness as social isolation and loneliness as emotional isolation.	"supportive social networks, together with close attachment relations, are fundamental to cope against loneliness, as we described in the theoretical framework in the re-examination of Weiss' typology of loneliness by DiTommaso & Spinner [22]", p. 145.	"Loneliness is linked to: lack of psychological support; depression; long empty days; separation from the family", (p. 153).<br><br>Conclusion includes a diagrammatic network portrayal of the category finding for "loneliness". (Key boxes in the Italian language.)<br><br>Main finding is that loneliness is "lived as a prison disease", abstract, p. 137.
[40] Fry et al (2017, England)	To look at wellbeing angles of the touring lives of professional golfers, so informing the globalisation/sport debate that	Interviews with 20 touring professional golfers.	Qualitative Interviews<br><br>NVivo coding and thematic organization of coded material.	Social isolation and emotional isolation: Social isolation is an absence of an engaging social network of other people; emotional isolation stems from the absence or loss of a close	Golfers' views quoted in the body of the article.	"The central aim of this paper was to use golf as a case study to examine the effects of globalization on the wellbeing of athletes as migrants in professional sport. In doing so, we have sought to explain, firstly, why golfers

	rarely if ever addresses such issues.			attachment relationship (Weiss, 1973).		(as migrant workers) come to feel lonely and isolated, and, secondly, how they attempt to address and make sense of such feelings and their life circumstances", p. 156.
[41] Goll et al (2015, England)	To identify barriers to access to forms of social participation of lonely older adults; to find out how lonely elderly people respond to those barriers; and to see how these barriers and responses relate to social identity.	N=15 participants (M, 5; F, 10), age-range 62-100, all bar one living alone (the one lived at home with her husband who had dementia).	Thematic analysis within framework of Constructivist Grounded Theory, based on analysis of transcribed interviews.<br><br>Independent coding and team corroboration leading to refinement of and consensus on themes.	Loneliness describes the distress that accompanies a perceived lack of social relationships. Older adults experience increased levels of loneliness, with possible links to increased morbidity and mortality.<br><br>Later-life loneliness is increasingly recognised as a major public health problem.	Loss of friends and family; illness and disability; loss of community; perceived lack of opportunity. These four "cluster" findings showed how the condition of loneliness is compounded for old people.<br><br>Telephone conversations provided a lifeline for some, reducing the feeling of being alone.<br><br>Household chores and screen-related activity could, for all subjects, mitigate loneliness.	"The study illuminated subjective barriers to social participation among lonely older adults, including both commonly cited and novel factors. The novel factors suggest that reductions in late-life social participation may reflect commonplace fears of social rejection/exploitation, and fears of losing preferred aspects of identity. Taken together, present results suggest that in order to enhance social participation among lonely older people it is necessary to address individuals' beliefs, fears, values and identities", p. 14.
[42] Hauge & Kirkevold (2010, Norway)	To investigate older people's understanding of loneliness.	An interpretive interview study exploring older people's understanding of loneliness and what they considered appropriate and effective ways of dealing with it.<br><br>N=30 participants, F, 21; and F, 9; aged between 70 and 97. 12 described	Interpretive qualitative design.<br><br>Analysis of qualitative understandings of loneliness, followed by more detailed examination of transcripts to identify themes that capturing participants' understandings, e.g.	Loneliness assumed to be both a universal and a culturally embedded phenomenon.<br><br>Loneliness seen as an experience that every person will, in some way, encounter. Some people may experience severe loneliness for long periods. Others	A striking difference observed between the way ''lonely'' and ''not lonely'' people talked about loneliness.<br><br>The "not lonely'' participants described loneliness as painful, caused by the person's negative way of behaving and a state they should pull themselves out of. The ''lonely'' participants also described loneliness as	This study underlines the importance of subjective experiences in trying to understand a phenomenon like loneliness and of developing support for lonely older people unable to cope on their own.

		themselves as ''lonely'' and 18 as ''not lonely.''	''disconnectedness'' and ''negative attitude.''<br><br>The third step was to identify the connection of the different themes to the participants' experience of being ''lonely'' or ''not lonely.'' This was followed by an analysis and interpretation of the themes.	may experience loneliness in special situations. Some may report an overall lack of personal experiences with loneliness, but may have encountered loneliness in other ways, such as by interacting with lonely people or hearing about others' experiences of loneliness.	painful, with more detailed descriptions of loneliness as disconnection from others, from their former home and from today's society. The ''lonely'' participants were more reserved and subdued in their explanations, attributing loneliness partly to themselves, but mostly to lack of social contact with important others. Some felt able to handle their loneliness, others felt unable to cope.	
[43] Heenan (2011, Northern Ireland)	To examine older peoples' wellbeing focusing on social isolation, loneliness and community capacity building.	Interviews with people 65+ years (N=35) taking part in rural community initiative 'Young at Heart' and with professionals – (e.g. GP, social worker) and volunteers working with them (N=16)	Qualitative interviews. Thematic analysis.	Links loneliness to social relations.	Identifies social networks, perceptions of the group (fitting in), knowledge building, leadership and valuing community as central to reducing social isolation and loneliness.<br><br>Promotes independence, social support, social networks and self-help to alleviate loneliness in rural communities.	Social networks can be developed and supported in rural areas, and local communities empowered and equipped to identify and tackle social isolation and loneliness.
[44] Hemingway & Jack (2013, England)	To explore social isolation in relation to an intervention involving 'friendship clubs' for older people. To explore reasons for attending or volunteering,	Reports on a 3-year research project exploring impacts of intervention seeking to reduce social isolation in older people through a network of 70 "friendship clubs". The study had access to 10 clubs.	Participative approach; Observation and individual/focus group interviews.<br><br>Interpretative analysis. Inductive content analysis.<br>Discussions about findings with the	(Boldy & Grenade, 2008) social isolation as a deficiency in social integration, and emotional isolation as a deficiency in intimacy and attachments. Social isolation regarded as an objective state: when an individual has minimal contact with others	Key themes:<br>1. Risk of becoming isolated<br>2. Feeling isolated<br>3. Friendship<br><br>These themes fell into 3 concepts:<br>1. Wellbeing<br>2. Social relationships<br>Health	The clubs provide the means (transport, location, & support) for older age groups to meet up to form friendships and support each other. Loss of responsibility causes loneliness. These clubs can help tackle the challenge and effects of social isolation for the older age group. The quality of the experience is essential to the

	experiences of barriers and expectations, and impact on wellbeing, mental and physical health. To identify measures to reduce social isolation from an older person's perspective.	The club attendees were over 80% F, average age of 80yrs, over 80% attendees lived alone, 4% attended more than 1 club if within travelling distance from home. 82 club members and 18 volunteers participated = 100.	participants and other stakeholders.	and/or a generally low level of involvement in community life. Measured by the number, type and duration of contacts between individuals and the wider social environment, an individual's social network. Other network-related indicators such as living arrangements (living alone), availability of a confidant, and community involvement.		club's success; must focus on valuing, supporting, educating and enabling attendees to fulfil their potential within their community.
[45] Hinton & Levkoff. (1999, USA)	To explore how family caregivers of those with dementia draw on cultural and personal resources to create stories about the nature and meaning of illness and to ask how ethnic identity may influence the kinds of stories told.	Explores how family caregivers weave together meanings of Alzheimer's disease with key life events, affective dimensions of experience, and personal history in their accounts of illness.<br><br>African-American, Irish-American, and Chinese-American caregivers. Total number of participants unclear; is likely to be F, 7; M, 1, mean age = 46yrs.	Narrative approach, unstructured interviews with family caregivers of elderly diagnosed with Alzheimer's disease or related dementia. In-home, open-ended interviews lasting 2-4 hours conducted in the family caregiver's preferred language (i.e., English, Spanish, Mandarin or Cantonese).<br><br>Thematic analysis. Narrative analysis	Links loneliness to loss.	1. Lost identities and deteriorating brains<br>2. Confusion and dependency in old age<br>3. Extreme loneliness and family losses	Each story type configures moral concerns in a particular way, and is revealing about how participants experience dementia and about what is "at stake" for them as caregivers. In each type of story, biomedical and folk understandings of Alzheimer's disease are combined. The stories point to distinctive "cultures" of health care important in shaping caregivers' interpretations of dementia-related changes. All narratives include descriptions of encounters with practitioners of biomedicine.

[46] Hislop et al (2015, England)	To examine how the use of mobile ICTs such as smartphones affected the experience of work of self-employed homeworkers, how they managed the work/non-work boundary and how it affected their experience of social and professional isolation.	Interviews with N=14 participants. All were administrators providing remote support to 1+ clients, with almost all work being carried out at home. Topics discussed included the nature of the work, feelings about it, its positive and negative features, the extent to which it was a source of stress or fatigue and how this was dealt with, as well as the role of ICTs and mobile ICTs and management of work-life boundary.	Qualitative: telephone interviews (~45 mins).<br><br>Content analysis. The findings are framed by combining Nippert-Eng's boundary work theory, with an 'emergent process' perspective on socio-technical relations.	Homeworking can create a greater sense of social and professional isolation due to a lack of formal and informal interaction with colleagues, peers, and managers (Mann & Holdsworth, 2003).<br><br>Cooper and Kurland (2002) defined professional isolation as: limited access to development opportunities, promotion, and reward. Morganson, Major, and Oborn (2010) define inclusion as: a sense of belonging, feeling, invited to participate in significant decision-making, and perceiving that your opinions matter.	1. The best & worst aspects of technology- dependent homeworking<br>2. The use of mobile ICT's and their impact on people's experience of work	The use of ICTs meant the home ceased to be the only possible workplace. The enhanced levels of spatio-temporal flexibility of mobile ICT allowed people to socialise through getting out of the home environment, while still being able have access to email and remain contactable by clients. While reducing a sense of general social isolation, the way the homeworkers used their mobile ICTs for work did not address professional isolation. The significant differences which exist between employed and self-employed homeworkers results in professional and social isolation being substantially different.
[47] Hollenbeck et al (2017, USA)	To investigate the virtual exchanges in survivor networks and whether these exchanges are valued for economic, symbolic, or expressive	The health industry is rapidly adopting digital services and face-to-face offerings are being replaced by e-services, e.g. peer-to-peer survivor networks for cancer patients. This study investigates virtual exchanges in survivor networks.	Interpretative approach used to investigate the role of P2PSNs (peer-to-peer online patient survivor networks). Narratives from forums downloaded and coded. Constant comparative method for cross-case comparisons of verbal texts with an emphasis	Weiss (1973): loneliness as a response to a social and emotional crisis stemming from the absence of satisfying relationships. Loneliness not caused by being alone, but by a relational deficit.	1. Gift system built upon agapic love.<br>2. Narratives as acts of selflessness<br>3. Agapic love identity: Extensions of an aggregate self	Those who have experienced trauma need to find others who can they can relate to. A conceptual model is presented for understanding the various gift systems that can emerge in virtual networks. Expressive worth is deemed valuable for cancer survivors and biographical narratives are an important component of

	worth. To address whether the alleviation of loneliness is possible.	All female, mean age = 62yrs. diagnosed with cancer and considering themselves "cancer survivors". Average annual household income for informants is $55,000. Participants had completed active treatment for cancer at least 1 year prior to the study. All 21 informants live in South-Eastern USA.	on peer-to-peer interactions.<br><br>21 semi-structured in-depth interviews with cancer survivors. As a preliminary assignment 1 week before the interview, informants asked to provide an electronic copy of their own narrative.<br><br>Hermeneutic analysis.	Weiss's (1973) "proximity-promoting mechanisms" explain how humans manage feelings of loneliness. Lonely feelings motivate connection and thus ensure safety. A lack of proximity-promoting mechanisms (offline or online) among cancer survivors leads to social isolation which has been shown to be an important predictor of adverse health outcomes and is associated with lower subjective well-being, higher morbidity and mortality rates. Prolonged feelings of loneliness.<br><br>Psychological and emotional healing begins when the survivor breaks down the walls of isolation and individualism that lead to loneliness and depression.		expressive gift systems. Once survivors engage in selfless giving, informants take on an agapic identity which fosters psychosocial well-being.
[48] Holtz et al (2012, Mexico)	To explore the psychosocial stresses faced by women with HIV/AIDS. To identify how	Private, in-depth, face-to-face interviews were conducted with 21 women receiving health services at the HIV/AIDS clinic,	Exploratory study with phenomenological roots. Semi-structured interviews (~30-60 mins).	Despite increasing rates of HIV infection among Mexican women of childbearing age, many must also maintain their role as family caregiver,	1. Fear<br>2. Social isolation<br>3. Anger/rage<br>4. Availability of resources/support<br>Resiliency	HIV-infected women in rural Oaxaca share similar psychological stressors and support needs documented in other groups of poor HIV-infected women. Adding to the

	HIV-infected women managed their lives with HIV/AIDS, and the resources needed to cope with HIV/AIDS.	COESIDA, near Oaxaca City, Mexico.<br><br>N= F, 21, ranged in age from 20-48 years (mean age =32.7 years). Most women had a 3rd grade education. 19 currently or had been married. 5 women's husbands had died from HIV/AIDS and 3 were unable to work due to illness. 13 had HIV-infected children. Participants had 11 children living with HIV/AIDS, and 2 of their children had died from HIV/AIDS. 17 participants worked outside the home. Most frequent occupation was as street vendors (N = 7). Weekly family income for families ranged from no outside income to 1500 pesos. Average weekly family income c $36 US.	Thematic content analysis	with physical and psychological consequences. HIV/AIDS poses a significant psychological burden, causing depression and anxiety while adjusting to diagnosis and facing living with chronic life-threatening illness, shortened life expectancy, complicated medical regimes, stigma from family and community, and loss of social support (Davies et al., 2009; Morrison & Cuadra, 2004). Reluctance to seek out treatment and support further isolated women, and is in contrast with other groups who have publicly demonstrated, demanding access to HIV/AIDS treatment, mental health services, and governmental benefits.		challenge of dealing with a potentially life-threatening illness, the high level of poverty, remote locations of their villages, and the absence of mental health/support services increased the psychological distress and isolation experienced by women in the study.
[49] Honigh-de Vlaming et al (2013, Netherlands)	To investigate how the different intervention components of Healthy Ageing – an intervention	Healthy Ageing is a complex intervention aimed at reducing the prevalence of loneliness among elderly Dutch people. This study aimed to assess how mass media communication materials,	Qualitative. Interviews. Thematic analysis.<br><br>3 Attention attracted by communication materials	Loneliness results in decreased mental and physical health and negatively affects quality of life.<br><br>Pathways for the alleviation of loneliness:	1. Attention attracted by communication materials<br>2. Acceptability of content intervention components<br><br>Themes did not necessarily relate to loneliness but instead evaluate the intervention.	Classical health education approach was not successful in reaching elderly persons at increased risk of loneliness. Important to involve the priority group in adapting the programme and to select practical strategies tailored to

	designed to reduce the prevalence of loneliness among elderly Dutch people – were received by the priority group.	information meetings and psychosocial courses were received by elderly people at high risk of loneliness.<br><br>Community-dwelling clients of the meal delivery service of local elderly welfare organisation were recruited, N = 17. Mage: 84 years and most lived alone.	4 Acceptability of the content intervention components	(1) network developing; (2) reduction of personal norms; (3) coping with feelings of loneliness.<br><br>Frequent involvement in social engagement activities appears related to better self-perceived health, better mental health and better physical functioning and loneliness.	Participants did not feel the intervention was relevant to them, they were too old to change habits and advertisements/posters were also irrelevant to them.	this group. In doing so, it is suggested that the objectives of the programme will become more relevant for the priority population.
[50] Houston et al (2016, Nepal)	To understand how the experience of widowhood affects Nepali women's social support networks and well-being and to examine the nature of social support impacted by a husband's death and its effects.	Between 2011-2012, 42 interviews were conducted in the Kathmandu valley and Surkhet district. Nepali widows aged 16 to 50 who were members of WHR were eligible to participate. Sampling frame was designed around: age (younger and older than 30 years); length of widowhood (1-5 yrs, 6-10 yrs, 11yrs.+), and; education (below and above completed grade 10).<br><br>N=30 single female members of WHR; majority were considered to be high caste (28) while 2 were low caste.	Semi-structured interviews (~45 mins). All interviews conducted in Nepali, audio-recorded. 2 transcripts manually coded by 2 researchers using grounded theory approach (Charmaz, 2006). Iterative process used to design a codebook.<br><br>Results interpreted based on an analytic framework in which social support is conceptualised into 4 major domains: emotional, instrumental, appraisal, and informational.	Social isolation conceptualised as a facet of the multidimensional nature of poverty (Sen, 2000) and as a key social determinant of health (Wilkinson & Marmot, 2003). Among Nepali widows, poverty is closely tied to the familial and societal dynamics surrounding their situation. Social exclusion and poverty may be cyclically related, as poverty fosters social exclusion, e.g. the stigma attached to widowhood, reinforcing a downward spiral into poverty.	1. Loss of emotional support<br>2. Social isolation and mistreatment within the home<br>3. Exclusion from family and community events<br>4. Vulnerability to verbal & physical abuse<br>Loss of instrumental support – economic insecurity	Results highlight the complex ways that social isolation faced by Nepali widows relates to their well-being. Suggests that Nepali widows are socially excluded. Loss of emotional and instrumental support highlighted as major ways through which social exclusion affects their well-being. Participants described lack of these kinds of supports as creating vulnerabilities to violence and contributing to the challenges that accompany and incite poverty.

[51] Howard et al (2014, Canada)	To describe the trajectories of social isolation experienced by adult survivors of a childhood cancer.	Long-term childhood cancer survivors may be at increased risk for poor social outcomes as a result of their cancer treatment, as well as physical and psychological health problems. Yet social isolation, is not well understood. Moreover, survivors' perspectives of social isolation as well as the ways in which this might evolve through young adulthood have yet to be investigated.<br><br>Cancer survivors who were diagnosed with cancer prior to 19 yrs old were recruited through follow-up clinics in British Columbia, Canada. A convenience sample of 30 long-term childhood cancer survivors participated. Participants ranged in age from 22 to 43 years (mean 31 years) and were 9 to 38 years (mean 22 years) from time of diagnosis.	Conducted in the context of a larger study examining medical and psychosocial challenges and needs of long-term childhood cancer survivors. Collection and analysis of data informed by the theory of relational autonomy.<br><br>30 in-depth interviews (45-120 mins); 20 conducted face-to-face, 10 over the phone.<br><br>Thematic analysis & constant comparison, inductive approach.	Social isolation is often conceptualised as a paucity of contact with others (social disconnectedness) and a perceived lack of social support, and feelings of loneliness and not belonging (perceived isolation). Social relationships and networks have been associated with poor health and survival outcomes among the general population, patients with chronic medical conditions, and adults with cancer.	1. Diminishing social isolation: it got somewhat better<br>2. Persistent social isolation: it never got better<br>Delayed social isolation: it hit me later on	Suggests that personal and environmental factors, e.g. self-esteem, supportive care needs and societal attitudes, influence social outcomes and play an important role in the lives of childhood cancer survivors. Future prospective longitudinal research that follows survivors as they age would help illuminate the relationships between early experiences, late medical and psychological late effects and social outcomes in adulthood. Survivors might benefit from information about potential social late effects as well as screening for social late effects during long-term follow-up. The findings suggest that one approach to providing support to prevent or mitigate social isolation is not appropriate — survivors with different trajectories will require different approaches at different points in time.
[52] Howard et al (2018, Australia)	To examine natural disaster risk perceptions reported by members of	This study examines natural disaster preparedness. 17 focus groups were held with 111 participants, members of 5 “at-risk” populations in	17 focus group discussions—of between 1-2 hours—were conducted with 111 community members.	Zavaleta, Samuel, and Mills (2014): social isolation is the inadequate quality and quantity of social relations with other	1. Historic & community knowledge<br>2. Access to physical resources<br>3. Increased reliance on the unknown	Findings highlight the multiple risks and impacts experienced in relation to natural disasters by already vulnerable groups when social isolation is a factor. Participant narratives across at-

	identified vulnerable groups. To determine levels of natural disaster preparedness and capacity to respond and recover. To analyse current and determine possible information and communication strategies to effectively support natural disaster preparedness.	regional Australia: older people, people with disabilities, families with children under five, low-income households, and people from culturally and linguistically diverse (CALD) backgrounds.<br><br>Participant characteristics included: Female (70%); male (30%). Person from a low-income household (47%) person from a household with at least one child under the age of five (17%). Person over the age of 65 years (37%) Person with a disability (31%). CALD person (20%).	Thematic analysis.	people at the different levels where human interaction takes place (individual, group, community and the larger social environment).<br><br>Social isolation considered as either subjective (an individual's perception) or objective (a quantifiable concept), but both concepts seen as coexisting intersecting, and interacting. Social isolation associated with increased morbidity. Predictors of increased social isolation include: biological factors, mental health; sociodemographic factors and physical factors (Aged and Community Service Australia, 2015).	Gendered experiences of preparedness	risk groups described social isolation as intensifying challenges in natural disaster preparedness and response. The impacts of social isolation in relation to natural disasters fell between the remit of even limited existing support networks. Further work in understanding both the impacts of social isolation and the potentially protective effect of relationships and networks is needed.
[53] Hubach et al (2012, USA)	To understand the mechanisms by which loneliness influences HIV risk in young men who have	This study explored the influence of loneliness in YMSM, in terms of HIV risk behaviour. Interviews were conducted with N=22 YMSM, 18–29 years of age, of HIV-negative/unknown status:	Following preliminary analysis of interview data from the first 15 participants, theoretical model began to emerge. Theoretical sampling then used to recruit 7 YMSM participants for	Martin and D'Augelli (2003) found loneliness scores to be higher in YMSM (young men who sleep with men) compared to their young male and female peers. Loneliness in young	1. A desire for connection<br>2. Indications of social and emotional loneliness<br>3. Relief through 'self-treatment'<br>4. The interconnection of desired relief and HIV risk<br>An iterative cycle of loneliness	Findings suggest need for enhanced behavioural and psychological interventions targeting YMSM at individual and group level. Current HIV interventions lack focus on social norms of sexual behaviour in the context of

	sex with men (YMSM) and to develop a theoretical model that can be used to shape social work intervention with this priority population and inform practice.	All reported at least two same-sex sexual experiences since becoming sexually active, and all were either HIV-negative or unknown HIV status. All resident in Southern California and reported at least one bout of loneliness per month for previous year.<br><br>68.2% participants reported at least 1 HIV test in lifetime, most reported last testing for HIV 7 months - 1 year ago (33.3%) or more than 1 year ago (46.7%). Of those testing for HIV, 86.7% reported most recent result as HIV negative, some had not returned for results.	in-depth semi-structured interviews. Sampling and data collection continued until theoretical saturation reached.<br><br>Grounded theory approach/open & axial coding.	men, both emotional and social, may decrease their likelihood to be in stable romantic relationships (Knox, Vail-Smith, & Zusman, 2007). Men often associate negative social outcomes with admitting lonely mental states, therefore they are less apt to seek professional help compared to their heterosexual counterparts. Loneliness influences YMSM's choices about their sexual behaviours. Some lonely and isolated YMSM may engage in activities that are related to unsafe sexual behaviours.		loneliness. Loneliness, psychological and sociocultural stressors should be addressed when providing HIV prevention programs. Social exclusion reinforces YMSM invisibility in society, and creates the impression that loneliness and poor mental health may be the by-product of social exclusion (Leary, 1990). Increasing evidence points to link between social exclusion, poor mental health, and HIV risk among YMSM.<br><br>Results indicated that loneliness is defined in two ways: emotional and social. A cyclical pattern emerged including negative symptoms, "self-treatment" of loneliness through drug use and sex, temporary relief, remorse related to engaging in HIV risk behaviours, negative self-image, the re-emergence of initial loneliness symptoms.
[54] Hurtardo (2014, USA)	To explore experiences of, and explanations for, social isolation among Latina immigrants from Central	This study explores trauma-exposed Latina immigrants' experiences of social isolation in the US and its perceived causes.<br><br>On average, participants were 43 years old (SD = 11.72) and had lived in the	Qualitative face-to-face interviews alongside demographic questions and measures to assess exposure to traumatic events, PTSD and depression.	As social beings, humans are integrated into webs of interpersonal relationships with implications for mental and physical health.<br><br>Berkman et al. (2000) contend that these social	1. Experiences of social isolation<br>2. Barriers to establishing social networks in the UK:<br>• Socio-economic<br>• Environmental<br>Psychosocial	Offers insights into participants' experiences of social isolation that can help develop social support interventions that take into account women's identified barriers to forging meaningful relationships. Little research has focused on the availability of supportive social networks in

	America, South America, and Mexico who were exposed to trauma.	US for 11.39 years (SD = 7.10). Most frequently represented countries of origin were El Salvador (32.1 %), Mexico (17.9 %), Honduras (17.9 %), and Guatemala (14.3 %). Other women were from Bolivia, Peru, Chile, and Colombia. Nearly all (89.3 %) reported at least one form of trauma based on screening with the SLESQ (Corcoran et al. 2000). Approximately half of the women (53.6 %) met criteria for presumptive depression and/or post-traumatic stress disorder.	Consensual Qualitative Research framework (Hill et al. 2005). Constant comparison.	networks provide means of social support, social influence, social engagement, and interpersonal contact, all of which have been shown to impact health.<br><br>Social networks must be present for social support to be provided and received (Cacioppo & Hawkley 2003).<br><br>Social isolation is a risk factor and can have a detrimental impact on mental and physical health. Lack of social support and limited social networks reflect 2 dimensions of social isolation: perceived isolation and social disconnectedness, respectively (Cornwell & Waite 2009).		this population. By including Latina immigrants from diverse nationalities, this study expanded a field that has tended to focus on Latinos from Mexico, Puerto Rico or the Caribbean.
[55] Janta et al (2014, England)	Explores the context in which international and domestic doctoral students encounter loneliness and social isolation,	This study used data gathered from online forums for doctoral students to understand how doctoral students cope with loneliness and isolation, and the tactics used during different phases of doctoral studies to overcome such issues.	Netnography (internet + ethnography). Specific threads were found using the following keywords: 'loneliness' and 'lonely'. Approximately 35 existing threads (in total 122 pages), launched between 2007 and 2011,	Berg et al. (1981) defines loneliness as the realisation of a lack of meaningful contacts with others and a lack or loss of companionship.<br><br>Hortulanus et al. (2006), suggest a lack of meaningful	1. Feeling lonely and isolated<br>2. Strategies for coping with loneliness and isolation<br>• Interacting with peers<br>• The doctoral forum<br>• Professional development<br>Escaping the academic world	Both domestic and international students experience social isolation, suffer a lack of emotional support and may struggle to engage in meaningful relationships with their peers. Social isolation is a problem that needs to be considered at the broader institutional and specific

	the tactics they use to overcome them and the solutions their peers offer.		such as 'dealing with loneliness', 'feeling frustrated and alone' and 'lonely', were downloaded, printed and analysed.<br><br>Thematic analysis.	relationships, which is increasing in the modern world, has a negative effect on the functioning and wellbeing of individuals.<br><br>Feelings of loneliness have been shown to affect academic performance as well as life satisfaction (cf. Lovitts 2001; Ali & Kohun 2009).		departmental level. Any promises regarding support made to prospective students in promotional materials and recruitment activities should be honoured.
[56] Jerrome (1983, England)	To understand the causes of loneliness and reasons why it is sometimes difficult to alleviate through exploring the experiences of women taking part in 'friendship clubs'.	Up to 250 participants were involved in this anthropological study of the significance of friendship for women in later life. All female; ages ranged from 55-90 but majority were in their 60's and 70's. Most lived alone and were single or formerly married. The majority held a number of active friendships with which they were satisfied. Most were in full or part-time work and had joined a club in order to meet people and make friends.	Anthropological. In-depth interviews, participant observation in group activities and one-to-one settings<br><br>Content analysis of club newsletters is the only form of analysis mentioned as being used.	Loneliness is a social and a personal problem; social isolation and loneliness are not the same thing (Hadley & Webb, 1975; Duck & Gilmour, 1981). Quality of relationships and expectations are crucial in producing a sense of relative deprivation and unhappiness with personal relationships. Isolation and loneliness can happen very suddenly with the loss of the partner, over-investment in a career, and reliance on working relationships at the expense of others.	1. A club for lonely people<br>2. Making contact<br>3. Characteristics of lonely women<br>Models of friendship	The experience of these participants offers clues to the failure of some people to make satisfying relationships. It indicates a felt need for friendship, which is seen as a positive resource making possible an expansion of self.<br><br>Friends are chosen because they reflect elements we like about ourselves. People who do not like themselves, not only have no real friends but seem to have difficulty with relationships in general.<br><br>The problem facing those in search of friendship, and professionals who might be helping them in the review of their relationships, is where to find it. Friendship clubs are

				People who have been part of a large and active network of family and friends may gradually become isolated through ill-health, rehousing, because they have outlived their contemporaries, or their children have emigrated.<br><br>Death or departure of significant other is prominent in accounts of loneliness at all ages.		unsuitable as bases for the development of satisfying, informal relationships.
[57] Kelchtermans et al (2011, Belgium)	To disentangle the complex and situated interactions' between principals' thinking about themselves and the particular context they have to work in.	Study was based on a secondary analysis of several studies on the work lives of staff members in Flemish (Belgian) primary schools. The authors argue that the gatekeeper, on the threshold between the outside-school and the inside-school world, is a powerful frame to capture some of the complexities of principals' emotional experience of themselves and their working conditions.<br><br>No detailed information on participants.	Secondary data analysis. Narrative-biographical and micropolitical perspective as a theoretical framework (Kelchtermans, 1993, 1996, 2007).<br><br>Semi-structured interviews. Interpretative analysis.	Structural loneliness and belonging (Nias, 1989; Ackerman & Maslin-Ostrowski, 2004).<br><br>The 'need to belong' plays a part in principals' work lives—especially since many used to be teachers and therefore part of a team. Their structural loneliness makes this a fundamental challenge.<br><br>Sharing leadership is based on the trust and belief that others can successfully deal with particular leadership tasks. The non-sharing principal remains lonely	1. Principal as gatekeeper<br>2. Caught in a web of conflicting loyalties<br>3. Struggling between loneliness and belonging<br>Establishing a professional self-understanding as a principle	It will be important to provide (future) principals with the relevant theoretical frameworks to analyse the complexities of their specific structural position in the organisation. This awareness and understanding allows for a more informed judgement on which to take action. Interactive exchanges among 'peers' may turn training into a powerful network environment that is supportive in developing relevant professional knowledge and skills, as well as a sense of professional self and belonging, that could transcend the loneliness of the gatekeeper (Kelchtermans & Ballet, 2006).

				(negative feeling), but the risk of having to account for the failure of others is limited.		
[58] Kharicha et al (2017, England)	To explore the perspectives of community dwelling lonely older people about seeking support for loneliness from primary and community based services and the features of these services which informed their views.	28 community dwelling people, aged 65+ who reported being 'lonely much of the time' or identified as lonely in a larger study, participated in in-depth interviews. Views and experiences on seeking support from primary care and community based one-to-one and group-based activities, including social and shared interest groups, were explored. Two-thirds of the participants were the 'younger old' and all were able to leave their homes independently.<br><br>N=28 participants: F, 18; M, 10. Age: 65–74 = 19; 75–84 = 5; 85+ = 4. Ethnicity: White UK = 25; Other = 3. 15 lived alone, 13 lived with others.	Interviews<br>Thematic analysis	Loneliness in later life is increasingly considered a public health problem (WHO 2002; DH 2012).<br><br>Loneliness is a subjective experience; an emotional and unpleasant response to a lack of satisfactory companionship (Heinrich & Gullone 2006).<br><br>In later life, loneliness is linked to other experiences associated with ageing, such as loss of family and friends and declining health and income, as well as socio-demographic trends such as longevity, living alone for longer, relationship breakdown, and changes to families and communities (Age UK Oxfordshire 2011)	1. Could befriending be for me?<br>2. Social groups are for others<br>3. Having a common interest<br>4. What can primary care offer?<br>Dealing with loneliness privately	Nearly half of the participants lived with other people and so would not necessarily be seen as socially isolated. Targeting social and other resources on older people living alone would likely miss this group. Older people were reluctant to seek help from their GP or practice nurse for loneliness, and social prescribing initiatives in primary care would require a pro-active approach to identify people who may benefit. Older people with loneliness who are able to leave their homes appeared largely ambivalent about services with a primary social purpose, perceived as being targeted for 'others'. More positive views were expressed of activity-based groups. Participants perceived a limited role for primary care, and many saw loneliness as a private matter that they wished to manage without external support.
[59] Kirkevold et al (2013, Norway)	To explore older people's approaches to living a life characterised	Based on a secondary analysis, data were collected through in-depth interviews at three different sites and by three	Qualitative interpretative design. Interviews were audio taped, transcribed, and analysed applying a	The findings provide an outline of loneliness as feeling isolated from others and a feeling of emptiness, which fits	Interviewees describing themselves as 'not lonely' viewed losses as normal, and they participated in meaningful activities, connected to other	Loneliness was associated with overwhelming losses, inactivity, meaninglessness, and social isolation. The contrasting findings between 'not lonely'

	by losses and 'aloneness' and how this relates to lonelines	different interviewers, one in each country during autumn 2006 and spring 2007.<br><br>N=15 participants (mean age of 79 years) were recruited from Queensland, Australia, 33 in the UK (mean age of 81 years), and 30 in Norway (mean age of 85 years). F, 55; M, 23.	hermeneutic, interpretative process.<br><br>The secondary in-depth analysis of the combined data set was guided by the analytical framework of Kvale (2007).	well with the definition of loneliness given by Weiss et al. (1973).<br><br>Loneliness is closely related to social status and health condition. Older people are vulnerable to experiences of loneliness due to losses, which follow the ageing proces	people and thrived in their own company. Those describing themselves as 'lonely' on the other hand, strove to create meaning in their lives, were overwhelmed by losses, had problems finding meaningful activities and difficulty keeping up social relations.	and 'lonely' older people have implications for nursing in that nurses must seek to identify those who need help in managing their loneliness and give guidance and support.<br><br>The findings indicate that older people's interpretations of losses in their life have a significant impact on whether they experience loneliness or not.
[60] Korumaz (2016, Turkey)	To explore the perceptions of school principals at Turkish elementary schools concerning loneliness, how they define it, what they see as its causes at school, how they overcome it and what the outcomes of loneliness are for them.	This study involved interviews and observation of N=7 Turkish elementary school principals.<br><br>7 participants (F, 1; M, 6); The age of participants varied from 34 to 59. Participants were in different phases of their careers; initiation (1-4yrs), development (4-8yrs) or autonomy (8-12yrs).	Phenomenological approach; triangulation method using three different data-collection methods, face-to-face semi-structured interviews, observations and field notes.<br><br>Thematic analysis.	Weiss (1973): loneliness is a lack of emotional satisfaction with social relations as they manifest themselves in reality. Principals' responsibilities combined with leadership bring loneliness and isolation to life 'at the top'. Principals work in isolation.<br><br>The degree of loneliness varies in severity; shaped by the working environment and the arrangement of positions within the organisational hierarchy (Wright, 2012). Climbing the professional ladder	Three main themes were identified: psychological insight, the organisational climate, and professional effort.<br><br>*Psychological insight* is the notion that all participants agreed on and emphasised when asked to offer a definition of loneliness at schools. Participants agreed that the *organisational climate* at Turkish schools represented the most significant reason for principals' loneliness at work. They invested *professional efforts* to overcome this invisible barrier	Contributes to a wider understanding of how school principals experience loneliness, the reasons for it, and the efforts that principals make to overcome it.<br><br>Principals mostly agreed on the definition, the reason and the methods they use to overcome loneliness. They preferred to define loneliness as a psychological insight; asserting that most common reason for loneliness was the organisational climate of Turkish schools. Principals were reluctant to overcome loneliness through their professional efforts.

				means an ascent to a 'summit of loneliness'. As a result, school leaders may make key decisions in a state of extreme loneliness (Stephenson, 2009).		
[61] Lanyon et al (2018, Australia)	To explore the particular experience of people with severe aphasia participating in groups, in order to elucidate the unique perspective of this population, as well as provide guidance to support their participation.	This study examined the reflections and experiences of N=7 (M, 6; F, 1), aged 49-79 years participants with severe, chronic aphasia.<br><br>They had had aphasia for between 1-10 years and their collective group participation experiences ranged from 2 months-9 years. 4 participants had participated in a speech pathology-led group, 1 in a co-led speech pathology and partner group, 1 in co-led peer and partner group and 1 in peer-led group. 3 were no longer participating in a group, whilst 4 maintained long-term group membership. All had participated in distinct groups. Most had severe expressive and moderate receptive language activity limitation and communicated through a combination of	Interpretative phenomenological approach.<br><br>Semi-structured interviews, field notes and journal entries. Transcriptions of interviews included detailing written, facial, and gestural expression, as well as verbal and vocal output (laughter, sighing). Analysis process involved reviewing video recordings and written transcripts.<br><br>Thematic analysis.	Focused on implied notion of social loneliness as lack of support and poor recognition of authentic self.	1. Assessing risk and realising gains<br>• Home as a communication refuge<br>• Seeking new connections<br>2. Balancing the need for authenticity with structure<br>• Creating a relaxed and social environment<br>• Seeking structure to support engagement<br>3. Acknowledging newfound confidence and skill<br>• The value of an enriched and authentic interaction environment<br>• The art of listening	This study highlights that people with severe aphasia are a group who are particularly vulnerable to harm which can be isolating and lonely. Conscious consideration must be given to addressing the potential barriers they may experience in successfully participating in community groups.

		gestures and isolated words.				
[62] Lee (1994, USA)	To examine experiences of loneliness as perceived by hospitalised adults with mental illnesses.	Interviews conducted with patients with significant mental illness.<br><br>12 adults, M, 9; & F, 3.<br>Age 29-76 (mean 47)<br>1 African American<br>3 with college education and 4 did not complete high school.	Qualitative. Content analysis used to identify major themes and categories.	Links loneliness to social relations	Identified 10 empirical categories which linked to interview questions: loneliness descriptions of self; loneliness descriptions of others; descriptions of not lonely; impact of family on loneliness; impact of friends on loneliness; what helps to feel less lonely; what makes loneliness worse; feelings associated with loneliness; beliefs and attempts to cope with loneliness; and mental illness and loneliness.	This study provided a preliminary examination of the subjective experiences of loneliness in a clinical psychiatric population. Subjects' ability to identify what made them feel less as well as ability to report loneliness experiences and what helped combat loneliness.<br><br>Key issues of disconnection, aloneness, and distractions as a way of combatting loneliness.
[63] Lindgren (2014, Sweden)	To illuminate the experiences of loneliness as described by people with mental ill-health.	Study was based on individual, informal conversational interviews, to derive the experiences of loneliness among people with mental ill-health. Recruited from social centres for those with mental ill health.<br>N=5 participants, 2 women, aged between 25 and 57 years of age.<br><br>Self-reported diagnoses were e.g. depression, anxiety, and psychosis. Length of experience of living with mental ill-health	Qualitative. Unstructured interviews: "Could you please tell me about your experiences of loneliness?"<br><br>Qualitative content analysis.	Links loneliness to social and emotional aspects of life.	Three themes: loneliness as multifaceted and Altering; as emotionally and socially excluding and as looking upon the world through a frosted window<br><br>Discussion links to the conceptualisation of loneliness given by Tillch (1963). Tillich (1963) suggests using two words for the multifaceted experience of loneliness, *loneliness* to express the painful dimension of being alone and *solitude* to express the developing dimension of being alone.	The experiences of loneliness were multifaceted and altering as well as emotionally and socially excluding. The latent meaning of these categories formed the theme looking upon the world through a frosted window.

		ranged between 7 and 30 years.			The experience of loneliness was described as not belonging and being set aside which authors argue links to Peplau & Perlman cognitive discrepancy definition.	
[64] Lou (2012, Hong Kong)	to investigate resilience factors that help Chinese older adults living alone cope with senses of loneliness.	Interviews conducted with N=13 community-dwelling older adults living alone. F, 8; & M, 5; aged 60 or above mean 75.5, ranging from 62 to 88 years old	Interviews. Used topic guide. Analysis used interpretive (hermeneutic) approach.	Uses Routasalo and Pitkala interactionist approach where loneliness in later life is a function of interactions between personal characteristics, resources, and life events. So includes a developmental view of personality and life events.	Authors argue that 3 culturally specific elements to the use of the model:<br>1. Self-management as a way of reducing family burden/enhancing family harmony and legitimating living alone.<br>2. Self-management strategies largely relationally focused; in and enhanced their collective self-esteem /collective wellbeing.<br>Social networks also valued kin/non-kin relationships equally.	Conclusions focus mostly on living alone rather than loneliness.<br><br>Family-oriented and relationship-focused resilience at the cognitive, self and personality, and social relations levels played significant roles in helping the Chinese older adults overcome their high risk of loneliness.
[65] Mackowicz (2018, Poland)	To determine factors that prevent loneliness.	Collective case study based upon qualitative interviews with N=17 centenarians who were not self-assessed as lonely.<br><br>Age range 100-107<br>M, 3; & F, 14; 4 with university education. 7 resident in nursing homes, 2 in convents.	Interviews.<br><br>Hermeneutic analysis conducted by two researchers independently	Links loneliness to interpersonal relationships	Describe commonalities not themes that protect against loneliness: active lifestyle; interpersonal relationships; optimism.	The only common aspect that weakened or neutralised the feeling of loneliness is the lifelong activity (broadly understood) of the respondents, both physical and intellectual and in combination with an optimistic approach to life
[66] McHugh Power (2017, Ireland)	To explore how socially isolated rural older adults	N=13 clients of a befriending service were recruited to participate in	Interviews recorded and transcribed verbatim. IPA used for analysis.	Existential, the cognitive, and the social needs perspective are cited in introduction. However,	Presence or absence of other people and their impact on feelings of loneliness;	Findings from this study have implications for how we theorise about loneliness since they suggest that our current

	understand the meaning of loneliness in Ireland and related implications for understanding of the link between culture and loneliness.	interviews (age range of 60–91 years, 77% female).	3 interviews were discarded because of quality leaving 10 including one male. This interview was excluded.<br><br>Analysis based on 9 interviews all women	authors state that the aim of the research was to understand how older rural adults understand loneliness, without providing a pre-existing theoretical framework of what this may be.	• Loneliness as a consequence of inactivity;<br>• Loneliness as a type of vulnerability; and<br>• Personal preferences, personal characteristics and loneliness.<br><br>It is suggested that themes 1 and 4 are often identified in aloneness research but 2 and 3 less common.	definition of loneliness being the discrepancy between actual and desired social contact (Peplau & Perlman 1982), may be incomplete. As well as considering loneliness in relation to social contact, a more complete definition may also need to take into account feelings of security in one's environment and one's ability to use activity to distract from or prevent loneliness. This is said to corroborate previous findings that loneliness may be related to personality and may remain relatively stable throughout the lifespan.
[67] McLaughlin (2018, England)	To investigate the role social support plays in how or in what ways postgraduate students make the transition to postgraduate level study and how social media use contributes to the development of this support.	Postgraduate students were recruited who had spent at least 1 year outside of higher education following completion of their original undergraduate degree.<br><br>Recruited via email.<br><br>N=9; F, 7; & M, 2, with an age range of 24–53 years.	Qualitative design informed by a thematic analysis strategy, and adopting a contextualist epistemological position.	Weiss -social/emotional loneliness plus cognitive gap.	Being in it together versus doing it alone – the problem of academic loneliness<br><br>Feeling apart versus feeling connected on Facebook.	Based on Weiss's (1982) frameworks, this *academic loneliness* appeared to be primarily a form of social loneliness, as it arose from a missing sense of connection with a group. It also involved some elements of emotional loneliness, as it was characterised by an unmet need for emotional support in dealing with personal difficulties.

[68] Milson (2003, UK)	To compare sexual murderers to rapists who didn't murder.	Case control study – questionnaires (UCLA Loneliness Scale) and semi structured interviews. Study done in prison – no mention of ethics permission, unclear how much participants could consent.<br>Nineteen sexual murderers and 16 non-murdering sex offenders took part in the study. All participants were male, over the age of 21 years, serving a life sentence. The mean age was 43 years, ranging from 25 to 58 years. 90% were White, 10% were African Caribbean.	Qualitative interview. Grounded Theory	Loneliness linked to social isolation.<br><br>Asked particularly about teenage loneliness and social isolation – labelled peer group loneliness.<br><br>Used UCLA loneliness scale to conceptualise loneliness.	The concepts of self as victim; grievance and loneliness may be correlated. The strength of such a co-rrelationship is, however, an empirical question and therefore needs further examination.	The results obtained in the study found that sexual murderers report feeling significantly more peer group loneliness than do rapists in adolescence.
[69] Morgan et al. (2020, New Zealand)	To comparatively outline the meanings of loneliness and social isolation from the perspective of four ethnically diverse groups of older adults (Māori, Pacific, Asian, and NZ European).	Interviews and focus groups with a diverse range of New Zealand older people (Māori, Pacific, Asian, and NZ European)<br><br>Interviewed participants (n=44; F, 36; M,8, aged 55 to 92years), to explore how diverse older adults conceptualize social isolation and loneliness<br><br>Māori (n =10; f, 9; m, 1) ; Pacific (n = 10; f, 9; m, 1); Asian (n = 10; all f); NZ	Qualitative design<br><br>Thematic and narrative approaches to analysis<br><br>A team of researchers (n = 8) collaborated to generate initial categories by discussion, which were subsequently developed into a series of descriptive and latent themes.<br><br>One of the researchers collaborated with the	Loneliness was conceptualised in terms of:<br><br>1. 'Look and feel'. The embodied nature of loneliness, frequently referring to it as physically and spiritually difficult emotion<br><br>2. Lack of desired companionship<br><br>3. State of mind	The ''Look and feel' of loneliness' concept included: physically identifiable characteristics (eg. Hair appearance) and emotions of anxiety, low mood, mental health, frequent crying and fear.<br><br>The lack of desired companionship concept included: lack of meaningful interaction rather than being on one's own per se, the degree of choice participants had in determining their social lives, self-worth (''deserving" (or not) of companionship or	Need for: more culturally appropriate services, increased mental health support, and weekend support provision. Interventions needed to battle racism and ageism in society and to remove any symbolic/structural barriers impeding older people's sense of inclusion in society. Further collaboration with diverse groups is required to improve effectively the healthcare system.

		European (n = 13; f, 7; m, 6); 'other' (n = 1, f, 1)<br><br>Three focus groups to discuss issues and strategies around social isolation and loneliness<br><br>Korean (n=4; f, 3; m, 1)<br>Chinese (n=4; f, 3; m, 1)<br>Mixed ethnicity (n=22; approx. 50/50)<br><br>Note: the great majority of participants were females	lead researcher of each culturally specific dataset to code the transcripts. NVivo-11 was used. When researchers' interpretations differed, the view of the lead researcher of each cultural dataset took precedence	4. The structural and situated nature of loneliness<br><br>Depression or anxiety were perceived as the most "serious" kind of loneliness	interaction), choice due to declining health.<br><br>Loneliness as a state of mind included: loneliness being a personal responsibility / fault, being a feature of being human or a part of their personality<br><br>The structural and situated nature of loneliness concept included: feelings of loneliness and disappointment in relation to the changes that are taking place in their neighbourhoods and family structures and customs.	
[70] Ojembe (2018, Nigeria)	To describe reasons for loneliness among older people in Nigeria.	Face-to-face interviews conducted with purposively selected group of N= 12 older adults aged 58–88 years. (F, 7; & M, 5) living in Port Harcourt Metropolis.	Descriptive phenomenological approach. Thematic Analysis	Loneliness is described as feeling lonely (Fokkema & Knipscheer, 2007) or being alone, which is seen as time spent alone or living alone (Victor, Scambler, Bond, & Bowling, 2000), and has been conceptualized into emotional and social loneliness (Weiss, 1973).	Results of the analysis revealed themes:<br>1. Perception and existence of Loneliness<br>• An unpleasant experience<br>• Feelings of helplessness<br>• Feelings of emptiness<br>• Withdrawal and thoughts of suicide<br>2. Factors for Loneliness<br>• Disability<br>• Living or being alone<br>• Age<br>• Weak family ties<br>• Bereavement<br>• Poor social networks<br>Retirement	Age is not a "standalone" factor for loneliness among older adults in Nigeria. Older adults can be lonely because of a combination of factors, including the presence of physical disabilities; the quality of relationships with adult children, friends, and relatives; and a lack of opportunity to be actively engaged in society.

[71] Ozawa-de Silva (2008, Japan)	To investigate internet suicide pacts and the postings about suicide on internet bulletin boards	Explored internet postings on suicide Web sites organised and run by a regular moderator with features such as a bulletin board (BBS) and chat rooms. Around 40 sites.<br><br>People posting to bulletin boards over several years.	Ethnographic study. No details in paper of methods or analysis.	3 essential characteristics of loneliness:<br>1. Involves perceived deficiencies in one's social world<br>2. A subjective state experienced by the individual rather than an objective feature of the individual's social world.<br>Experienced as unpleasant and distressing (Kraus et al. 1993, p. 37).	Loneliness can be seen to involve two factors: the external environment and effect of social forces on the individual, and the attitude or perception of individuals themselves.	For Japanese people experiencing afflictive loneliness, it would be more comfortable to die with others than alone.
[72] Paque (2018, Belgium)	To explore general feelings among nursing home residents, with a specific interest in loneliness in order to develop strategies for support and relief.	Data were obtained through face-to-face interviews with (N=11) nursing home residents. Age in years: mean (range) 84 (74–92). F, 7; & M, 4. Years since admission: mean (range) 4 (1–10).	Phenomenological study. Qualitative open-ended interviews. Interpretative phenomenological analysis used for data analyses.	Loneliness is more than being alone among others.<br><br>Loneliness defined as a subjective, unpleasant and distressing feeling resulting from the perception of a discrepancy between one's desired and achieved levels of social relations<br><br>Loneliness is the social isolation experienced when one's social needs are not being met by the quantity nor the quality of one's social relationships.	Participants' descriptions of loneliness varied from aloneness to feeling unappreciated, boredom, not feeling at home in the nursing home and loss of autonomy and self determination.<br><br>Loneliness can be related to physical decline and a loss of functional autonomy.	Be aware of these feelings and pay attention to resident preferences while developing (individualised) interventions to prevent loneliness.<br><br>Participants' loneliness was all about the perceived quality of their relationships with important others and an unfulfilled need for meaningful relationships. Feelings of loneliness had little to do with the number of contacts, getting regular visits or participating in group activities, although these aspects seemed to reduce loneliness.

[73] Park (2017, New Zealand)	To explore the perceptions and experiences of social isolation and loneliness of older Asian migrants in New Zealand and to identify the strategies that they use when experiencing social isolation and loneliness in their foreign environment.	Interviews and focus groups conducted with 25 participants. For face-to-face interviews, recruited 10 older people who were migrant parents from Asian countries (mainly China and South Korea). Participants were required to be aged over 65.<br><br>Participants in the three focus groups included older Chinese migrants in one group (N=6: F, 3 & M, 3); Korean migrants in another N=4: all females); and Chinese professionals or service providers (N = 5: F, 3; & M, 2).	Used two qualitative methods (interview and focus group) with a critical, social constructivist perspective<br><br>Thematic analysis.	Loneliness and social isolation are based on cultural norms that do not have universal applicability (Heravi-Karimooi, Anoosheh, Foroughan, Sheykhi, & Hajizadeh, 2010).	These participants experienced feelings such as sadness, uselessness, and hopelessness which, in turn, were closely related to feelings of loneliness in their daily lives<br><br>Such "lonely" feelings were mostly perceived by participants but not expressed (or ignored) by them, as their emotional problems were often subtle and overshadowed by physical symptoms, such as insomnia and tiredness.	Ollder Asian migrants in New Zealand experience a form of "double" isolation and loneliness in at least some points of their migrant lives.<br><br>Ageing in a foreign land involves a range of challenges, among which are social isolation and loneliness mainly caused by the lack of family contact or support in a transnational family setting
[74] Pedersen (2012, Denmark)	To explore the relationship between social relations and social isolation among socially marginalized users of shelters and drop-in centres in Denmark	46 in-depth, face-to-face interviews, 46 participants – M, 39; & F, 7 were interviewed, ranging in age from 22 to 64. Six interviewees were born outside Denmark (in Greenland, Iceland, Germany, Iran or Somalia). All interviewees lived on social security benefits. At the time of interview, 32 were homeless, whereof 29 lived at shelters and three lived on the streets.	Thematic analysis	Authors present a diagram to illustrate loneliness connected to social relations.	Data enabled the construction of a typology of five groups according to different combinations of social relations and perceived social isolation. The five groups were: the socially related and content; the satisfied loners; the socially related but lonely; the socially isolated, and; a group of in-betweens. The relationship between social relations and social isolation was ambiguous and complex and did not seem to be associated with interviewees' degree of social marginalisation.	Variation was found in the social relations and experiences of social isolation among the interviewees, indicating an ambiguous and complex relationship between the two and loneliness

[75] Pettigrew et al (2014, Australia)	To explicate lay theories relating to social isolation and identify instances of to inform future efforts to encourage older people to participate in protective behaviours. Eventual aim being to identify potential interventions.	Sample of 111 community-dwelling Australians aged 40+ participated in a series of individual interviews (N=20) and focus groups (12 groups, N=91). Four focus groups in regional area, remainder in Perth, WA. Quota sampling to ensure age and gender balance. Invited to discuss a broad range of topics related to health and wellbeing through non-directive opening questions. Further discussion emergent. Interviews audio-recorded and transcribed.	Qualitative. Interviews and focus groups. Non-directive grounded theory approach. NVivo.<br><br>Thematic analysis.<br><br>Analysis paid specific attention to data relating to social isolation; thematic analysis identified lay theories relating to causes and consequences in later life, and instances of positive deviance (behaviours that enable individuals to survive and thrive despite experiencing social isolation).	Social isolation connected to social connection and defined as comprising both objective isolation (size of social network) and subjective isolation (perceptions of quality of relationships i.e. loneliness). Recognises the possibility of relationships being negative; that the mere presence of other people does not ensure individuals feel socially connected.	Two lay theories relating to social isolation identified: (1) Importance of social connection; (2) Developing social connection is harder in later life.<br><br>Examples of identified included: (1) a view of the process of overcoming social isolation as incremental, (2) being prepared to instigate social interaction, and (3) the recognition of a need to adopt 'external focus'.<br><br>These strategies reported as being effective in increasing social interaction.	Participants tend to identify social isolation as occurring in others rather than themselves, indicating the presence of stigma and the need for interventions to address this.<br><br>Difficulties associated with encouraging people to instigate new relationships while established social networks shrink.<br><br>Interventions may need to include general population as well as targeted approaches.
[76] Piat et al (2018, Canada)	To examine experience of loneliness among people with psychiatric disabilities who have moved from custodial housing into independent or supported living.	Interviews with tenants (N=24) in 5 supported housing sites across 3 Canadian provinces, and with designated family members (N=15) and case managers (N=19). Group interviews with housing workers (N=17) in each site. Interviews transcribed and analysed thematically. Participants aged 18-64, previously in custodial housing, with diagnosed	Qualitative. Naturalistic / constructivist theoretical framework. Semi-structured individual and group interviews. Inductive, thematic analysis.	Loneliness defined as 'the subjective experience of unmet social and emotional needs associated with social isolation' (ref Perese & Wolf, 2005).	Participants experienced loneliness as something that could be managed or overcome.<br><br>Four themes identified:<br>1. Taking a position on tenant loneliness – eg. denying it, seeing it as something that can be anticipated or managed, the price of freedom<br>2. Confronting loneliness in supported housing – strategies to alleviate	Living in supported housing does not typically engender loneliness. Participants recognised independent living as normalising – even more so when loneliness was viewed as a 'universal human experience'.<br><br>People with psychiatric disabilities may enjoy the opportunity to be alone.

		psychiatric disability but no intellectual deficit. Gender, relationship, profession type and length of stay recorded.			Appraising loneliness through past experience	
[77] Pirhonen et al (2018, Finland)	To explore perceived social isolation of older people moving from private homes to residential accommodatio n and their adjustment to new environment.	Two-month ethnographic observation. 10 unstructured thematic interviews with residents in sheltered housing site. Observations in section where 114 residents lived in 8 group homes. Most cognitively impaired, some with physical or other chronic conditions. Few able to leave site independently. Physical abilities varied from being independently mobile to being bed-ridden. 70% women. Age range 60-100+.	Directed content analysis. Data searched for references to experiences of social isolation. Two phases: (1) first author searched data (2) excerpts categorised on basis of reasons for feeling socially isolated. First and second authors coded, third participated in interpreting. Triangulation achieved by 3 researchers combining 2 qualitative data source types.<br><br>Prior conceptualisations informing: Nussbaum's definition of 'affiliation' (2007, 2011) and recognition of two social worlds: inside and outside the facility.	Explores perceived social isolation, understood as both subjective and objective and linked to situation and surroundings. Social isolation seen as 'a sense of not belonging' (Weiss, 1973), a 'lack of communion' (Victor, Scambler & Bond, 2009). Loneliness in assisted living as an imbalance in one's actual and desired social worlds.<br><br>Uses Victor, Scambler and Bond's conceptualisation of social isolation and Nussbaum's (2007, 2011) concept of 'affiliation'.<br><br>Affiliation is two-dimensional capability: the ability to engage and connect with others and 'being able to be treated as a dignified being whose worth is equal to that of others' (Nussbaum 2011: 34)	Social isolation connected to two separate worlds, inside and outside the facility.<br><br>Themes explored: (1) detachment within the assisted living facility, (2) separateness from the social world outside the facility.	Social isolation seen as 'rupture of affiliation' in the context of the move to assisted living.<br><br>Perceived social isolation reduces affiliation.<br><br>Social isolation results from factors connected to quality of social interaction with co-residents and staff, daily routines of institution, and personal life histories.<br><br>It may be more important to remove structures that further social isolation than to promote the benefits of affiliation or social connectedness.

[78] Pramuditha et al (2014, Canada)	To uncover experience of loneliness among older Sinhalese immigrant women in Toronto, Canada and to explore how they experience loneliness in the post-migration context.	Study conducted for Masters thesis. Grounded in personal experience of first author. Initial and follow-up interviews conducted in Sinhalese with 2 Sinhalese participants asked to tell story of coming to and living in Canada. Both participants female, aged 65+. Arts-informed data collection and verbal interview. Observational field notes. Participants drew pictures of experience of loneliness post-migration and these and personal objects included as data.	Qualitative. Narrative research. Interviews and observation field notes alongside arts-informed data collection tools. Interviews audio-taped, translated and transcribed.<br><br>Resulting narratives checked with participants.	Informed by theory of relational loneliness (Weiss, 1973) – two types of loneliness, social and emotional.<br><br>Reflection on narratives suggests multidimensionality of loneliness (ref Gierveld 1998).	The author notes the impact of emotional state of loneliness on physical health and discusses implications for gerontological nursing in immigrant context.	Even though an immigrant woman may be surrounded by family, she may still be lonely. Importance of 'personal and aesthetic knowing' by nurses and researchers to understand diverse life experiences and support care.
[79] Rokach (1988, Canada)	To develop a model of the experience of loneliness.	Verbatim accounts of loneliness collected from opportunistically recruited participants over a 5-year period. In a group setting, subjects asked to describe their loneliest experience, including thoughts, feelings and coping strategies related to it. Short meetings and informal discussions also held following completion of exercise. N= 528 Participants. F, 298; M, 228. Average age 33.7, majority aged between 19-45.	Qualitative content analysis of verbatim reports of loneliness gathered from opportunistic sample of individuals over an extended period.<br><br>Qualitative content analysis. No prior hypotheses about nature of loneliness formulated and no existing questionnaires used. Subjects asked to respond to the instruction: 'Describe	Super-structures identified include: self-alienation, interpersonal isolation, distressed reactions, and agony. In loneliness these are combined to create a unique phenomenological structure.	1.Self-alienation: has two components = emptiness and depersonalisation<br>2.Interpersonal isolation: has three components = absence of intimacy, perceived social isolation, and abandonment<br>3.Agony; has 2 components = inner turmoil and emotional upheaval<br>4. Distressed reactions: has 4 components = physiological and behavioural distress, self-depreciation, self-generated social detachment, and immobilisation.	Loneliness is a painful experience involving both self-alienation and interpersonal isolation and generating distressed reactions by the individual.<br><br>Experience differs qualitatively among individuals.<br><br>Will always include the 4 major elements but not necessarily all the underlying components and factors. Individual differences determine which factors are prevalent.

		Education levels varied, but 67% had completed 2 years in university or college. 63.2% single.	your loneliest experience'.		Author suggests results support definitions of loneliness by Moustakas (1961) as an 'experience of raw sensitivity' and Young (1982) as 'the absence or perceived absence of satisfying social relationships, accompanied by symptoms of psychological distress'.	Loneliness is not equated with emotional disturbance or mental deficiency. It is all-consuming and affects the totality of the person.
[80] Roos & Klopper (2010, South Africa)	To understand and describe older persons' experiences of loneliness, their perceptions of factors contributing to it and ways of managing it.	Interviews with 31 older persons living in Potchefstroom district in North West Province of SA. Interviews conducted in participants' own language and transcribed verbatim. Location purposively selected because of high number of residential care facilities and older people in the population. Gender: M, 3; F, 28. Mean age = 74. Ethnicity: blacks = 15, whites = 16. 90% of were widows or widowers. Majority owned a house or flat in a retirement village or lived in residential care.	Qualitative. Phenomenological approach. Individual in-depth interviews.<br><br>Emphasis on loneliness as a lived experience. Phenomenological methods (Giorgi & Giorgi, 2008).<br><br>Researchers required to bracket prior knowledge of loneliness, and sensitivity towards subjects' descriptive experiences.<br><br>In discussion, authors identify what they term a 'general structure of the experience of loneliness'.	Loneliness for older people is regarded as a subjective lived experience (Wenger et al, 1996) existing in the form of multiple realities constructed (Victor et al, 2009) through the lack of satisfying human relationships, and causing distress.	Identified themes are grouped:<br>1. Expressions of loneliness as painful and unpleasant, related to the passing of time, loss and limitations<br>2. Causes of loneliness – predominantly the result of limited choices and loss of all kinds<br>3. Coping with loneliness – strategies identified included self-awareness, humour, preparation activities, engaging in meaningful contact, religious faith and activity, and active engagement with others.	Loneliness seen as a multi-faceted, unique, personal experience informed by individual's world-view and situated within a particular socio-historical and cultural context. Needs to be understood in relation to the environment. For example: loneliness for older black participants particularly associated with limited choices available as result of socio-cultural context of discrimination.<br><br>Recommendations for dealing with loneliness informed by psychosocial developmental theory (Erikson, 1963).
[81] Roos & Malan (2012, South Africa)	The purpose of this study was to explore older people's	The research was conducted in a residential care facility in Johannesburg in the	Exploratory research methods	For the purposes of this study, loneliness was regarded as a relational phenomenon, and the	*Loss of meaningful interpersonal interactions.* Almost 40% of the participants reported that they had lost meaningful	The results showed that older people experienced loneliness in terms of having unavailable interactions due to loss, and an

	experiences of loneliness in the context of institutionalized care.	Gauteng Province of South Africa. Data on the subjective experience of loneliness were gathered through the Mmogo-method, whereby drawings were employed to explore matters and issues of importance in the lives of older people that could be used to deal with loneliness.<br><br>Participants were:<br>10 older persons (F, 7; M, 3) with ages ranging between 62 and 82 years. 7 participants were widowed, one was single, one was divorced, and one was married.	The collected data were transcribed and analysed using thematic analysis and key-words-in-context analysis, which contributed to the trustworthiness of the research. The visual presentations and drawings were analysed by means of visual analysis.	theory of complex responsive processes of relating was used as the study's theoretical framework.	interpersonal interactions through death. This specific loss was identified as the main reason for their loneliness.<br><br>*Absence of current meaningful interpersonal interactions.*<br>Three participants reported not having anybody to talk to about their feelings and experiences of loneliness in their current interpersonal context.<br><br>*Regular contact.*<br>The participants who reported having regular contact with other people said that they did not experience loneliness.	absence of meaningful interpersonal interactions. Meaningful interpersonal interactions were described as when the older people had regular contact and a variety of interactions. Ineffective interpersonal styles (e.g. taking a controlling position in relationships and being rigid) elicited rejection and isolation, and were associated with a lack of confirmatory interpersonal relationships.
[82] Roos et al. (2019, South Africa)	To understand loneliness of a group of older women who have been rendered vulnerable by longstanding exclusion from community, services and material resources.	Sixteen Setswana-speaking women in Ikageng, a township in North West Province<br>of South Africa (age 61–73), participated in the Mmogo-method® and open-ended interviews.<br><br>Textual data were analyzed using thematic analysis, visual data analysis of elements and symbolic representations of loneliness.	Qualitative – culturally sensitive methods and interviews<br><br>A critical-realist ontology underpins this qualitative<br>study and informs the notion that multiple, subjective perspectives of older individuals reveal patterns or indications of loneliness as social reality.	Loneliness used as a metric for exclusion from social relations.<br><br>Loneliness defined as a powerfully unpleasant experience of not being able to interact with other people in general, or more specifically as a result of the loss of particular people (including spouses, parents and children) and isolation provoked by the impact of	Social loneliness and emotional loneliness interconnect<br><br>Three types of experiences contribute to loneliness:<br>(1) loss of intimate relationships to particular people (spousal – loss of care and protection)<br>(2) not being able to interact with people poor health, exclusion, forgotten/disrupted communities, discrimination and mobility issues; and (3) impact of painful interactions. (humiliation, shame judgement)	Loneliness is fundamentally important though differently experienced across settings. In predominantly<br>Western context, loneliness is conceptualised as an unpleasant experience arising from a lack of sufficient<br>quality personal relationships. Among older indigenous<br>black South African women left vulnerable in<br>terms of their group history of oppression, loneliness<br>is regarded as an intensely unpleasant experience that

		The Mmogo-method® according is an example of an Afrocentric methodology that is applied in four phases. Phase 1 intro and Rules; Phase 2 Clay, bead, stalks cloth creation of representation of loneliness. Phase 3 explanation. Phase 4 reflection/review debrief.<br><br>Trained field workers conducted open-ended individual interviews of 45–60 minutes with participants. Three open-ended prompts were used: What can we learn from you about loneliness? Help us to understand what contributes to loneliness. What can help to counter loneliness?		relational interactions and group dynamics. Loneliness was mitigated by socializing and gathering for traditional activities, performing spiritual rituals, and keeping busy individually or with others, thus reinforcing a core theme that any social interaction alleviates loneliness.	Participants in this study used three main strategies to deal with loneliness: interacting with anyone who is available; engaging in socio-cultural and spiritual rituals; and keeping themselves occupied.	manifests when there is no one to engage with. The findings of this study point to a previously unexplored understanding of loneliness resulting from disrupted community embeddedness.<br><br>We have much to learn about how loneliness might emerge from their patterns of developing, maintaining and losing social relationships.
[83] Russell & Schofield (1999, Australia)	To explore how aged care practitioners define, identify and manage social isolation in their clients.	10 semi-structured interviews conducted with purposive sample of 18 aged care practitioners, individually, in pairs, or as a group. Included 8 students at University of Sydney, colleagues of students and 5 hospital-based workers. Participants asked for definition of	Qualitative. Interviews.<br><br>Grounded theory approach. Emergent categories generated through constant comparative technique.	Looks at social isolation and contact with others as issue for loneliness.	Notes that all respondents define social isolation with some reference to an 'objective state of having minimal contact', but that they also perceive this to be insufficient in that subjective aspects also need to be considered.	Identification, management and provision of services to isolated older people can be conceptualised as an interpretive social practice occurring in specific relational settings and constrained by social structure.<br><br>Constraints include: inadequate resource allocation, time

		social isolation, perceptions of social isolation among clients and practices used in working with socially isolated clients. All participants female, mixed professions.				pressures and instrumentality and organisational and professional rules.
84 Sa'ar (2001, Israel)	To explore the code of familial commitment among Israeli-Palestinian (I-P) women, including the interplay between power and gender.	Presents 4 demographically varied case examples of Palestinian women living in Israel. Aged between 30-40. Discusses their experience of isolation, gender and familial relations.	Qualitative. Anthropological / ethnographic case studies.<br><br>I-P women's experience of family relations does not always align with the 'official ideology' of the Palestinian family. All four cases discussed in this paper are of women who experience particular loneliness within such a structure. The author explores the reasons for this, referencing gender / power relations.<br><br>Discussion in the light of anthropological literature relating to Arab and Middle Eastern societies.	It is possible to be lonely within a family. Focus on relational aspects, isolation and failed familial fidelity.<br><br>References Winnicott (1958), voluntary isolation as a revelatory experience in the Koran, and From-Reichmann (1959) view of 'real loneliness' as harsh and mentally destructive.	Although I-P men might be isolated or feel lonely, they will generally experience unconditional family fidelity and will very rarely be deserted or ostracised, whereas these are common 'punishments' for women. As a result, I-P women are susceptible to isolation and loneliness and failed familial identity.	Women in I-P families susceptible to becoming isolated and lonely because of the code of familial commitment.<br><br>In gender-segregated societies, the status of women determined according to a gender-specific scale rather than in comparison to men.<br><br>The key variable determining whether or not an I-P woman experiences relational loneliness is her position along a continuum between power and weakness / masculinity and femininity.
85 Salas et al (2018, England)	To explore traumatic brain injury (TBI) survivors' subjective	Study of social isolation and friendship with chronic stage TBI survivors (N = 11, M, 9; F, 2) who attended a social	Semi structured interviews.	Social isolation arises due to physical, cognitive and behavioural changes and	Four main themes emerged from the interviews:<br>(1) The impact of long-term cognitive and behavioural problems on relationships;	Rehabilitation programmes should focus on creating a safe, relational space for survivors and their families, as relating to other survivors is both a way of

	accounts and understandings of the challenges encountered in sustaining friendships	rehabilitation day programme.<br><br>Participants, had an average age of 49 years (SD=9.6; min=30, max=63), and the average number of years since the injury was 17 (SD=8.8; min=5, max=33).	Theory-led thematic analysis of interview data.	emotional responses following TBI.<br><br>Survivors become less socially active and experience a marked decrease in the number of friends.	(2) Loss of old friends;<br>(3) Difficulties making new friends, and<br>(4) Relating to other survivors in order to fight social isolation (sameness).	resisting cultural discourses about disability, and offers a source of self-cohesion in the process of identity re-construction.
[86] Schirmer (2015, Sweden)	To examine how different stakeholders frame loneliness among the elderly as a social problem.	Study of professional perspectives on loneliness. Participants were 23 people from different professions who work with elderly people.<br><br>Participants included F, 19; & M, 4; aged 19-85 (mean = 57). They include priests, social workers, elderly care personnel and geriatric nurses as well as seniors in voluntary organizations and retirement associations.	Semi structured interviews. Inductive and theory driven coding. The analytical strategy was to find the explicit and implicit distinctions used by the respondents in explaining loneliness among the elderly.	Focuses on social explanations of loneliness and the way that these invoke nostalgic and distorted contrasts between traditional and modern societies.	Respondents identify bio-medical, psychological and social causes of loneliness. Social explanations include:<br>(1) city vs village (the suggestion that urban environments promote anonymity and lack solidarity).<br>(2) Past vs present (the attribution of loneliness to changes in family structures and intergenerational contacts.<br>(3) Modern vs past work life (contemporary working arrangements leave less time available for the elderly).<br>(4) Geographical distance/mobile vs proximity/fixed (changes in labour markets have caused greater mobility, increasing the between family members and friends.<br>(5) Technology vs human interaction (technologization of society is said to make people	The lost Gemeinschaft:<br>Such a nostalgic and melancholy view is ahistorical and simplistic. It overlooks the progress of modern society in terms of longevity, social institutions, scientific advances and pluralism of values and lifestyles. At the same time it also downplays negative aspects of traditional societies. In terms of practical relevance, there is merit in offering more ‘Gemeinschaft’ to elderly people through excursions and activities, provided the elderly individual can participate on her/his own terms, and not because of pressure exerted by care personnel or peers.

					feel lonelier by reducing face-to-face interaction).	
[87] Smith (1998, Australia)	To explore the mechanisms of alienation of househusbands and identify some of the adaptations men make to deal with these experiences.	Study of men's (N=11) experiences of alienation following the decision to give up full time careers to stay at home and care for children. to Participants were all supported by women who had given up full time child-care and housework and taken up full time paid work. All were white, educated, English speaking Australians.	In depth interviews. Qualitative coding and theme development	Ideological isolation arising from hegemonic masculinity that renders it socially illegitimate for men to be involved in full time child care, to be disengaged from the workforce, and to be supported by the earnings of women. Social isolation is conceived as a problem of the validation of transgressive gender practices. Househusbands experience negative reactions from others to their form of life; and they are relatively powerless to transform the views of others and therefore lonely.	Men faced negative reactions and difficulties being integrated into the group of their potential peers (mothers). Men's attempts to overcome isolation by positioning themselves as e.g. "competent housekeeper" or "full-time househusband and child carer" were not effective: Hegemonic conceptions of who ought to be minding the children and the house subvert or thwart these men's attempts to validate themselves and these practices.	The influence of hegemonic constructions of childcare and housework roles engenders feelings of illegitimacy results in social isolation of househusbands.
[88] Smith (2012, USA)	To understand how older adults experience and cope with loneliness and its effect on their health and wellbeing.	Study of loneliness with 12 older adults (F, 8; & M, 4; aged 74-98) who identified as lonely and were in contact with services.	IPA.<br>Narrative interviews (3 per participant) conducted every 3 to 4 weeks.<br><br>Coding to uncover the meaning of loneliness to older adults and how	Loneliness is a result of disrupted meaningful engagement with others due to different age-related changes and losses (health, social status, friends, and/ or spouse). Loneliness is an embodied experience can be expressed	<u>Coping practices</u> - a striking theme was the importance of maintaining connections with others, by visiting friends, going to lunch, or telephoning family. Volunteering was another avenue to reach out and help others, which was empowering and satisfying for many.	Findings revealed that many participants experienced loneliness as a result of disrupted meaningful engagement with others due to different age-related changes, including changes in health and mobility, retirement, widowhood. Loneliness was embodied as it was expressed

			they coped with loneliness.	through the body in several ways, including fatigue, tension, withdrawal, and emptiness.	Health and wellbeing - declining physical health and functional limitations interfered with participants' ability to maintain meaningful engagement with others, which contributed to loneliness.<br>Embodiment - participants experienced both emotional and physical sensations of loneliness. Participants with sensory impairments such as loss of hearing or vision are at a heightened risk for loneliness because of the social isolation such impairment creates.	through the participants' bodies in several ways, including fatigue, tension, withdrawal, and emptiness.<br><br>Conclusions from this study reveal the need for nurses to become more aware of loneliness in older adults as a significant health issue.
[89] Stanley et al (2010, Australia)	To explore older people's and service providers' perceptions of loneliness in order to inform programmes that address loneliness and to help maintain an integration of older people in the community.	Study of loneliness in older adults in four large service provision organisations. Participants were 60 people aged 65 and over (30 from each State) including F, 40; & M, 20; with an age ranging from 67 to 92 years. There were 8 focus groups with service providers, averaging 8 participants per group.	Interviews with older people and focus groups with service providers.<br>Inductive thematic analysis of qualitative data by two researchers.	Loneliness and social isolation not specifically defined.<br><br>Risk of loneliness in older people due to losses, physical limitations. Not all people who are socially isolated are lonely. and people who have many connections may still experience loneliness.	Five dimensions emerged:<br>1) private (loneliness as personal, negative and often stigmatized).<br>2) relational (quality, not necessarily quantity, of relationships is important)<br>3) connectedness (to the community and wider society)<br>4) temporal (loneliness is related to time, including time of day, time of the year or more broadly to the time of life).<br>5) readjustment (this theme from focus group data alone), staff emphasised the ability of older people to adjust to change and losses, and to the prospect of their own mortality.	Loneliness is a diverse and complex experience, bound to the context in which it is experienced.<br>It is a subjective experience with overlapping dimensions and is not synonymous with social isolation.<br>Our findings show that loneliness is influenced by private, relational and temporal dimensions and whether older people feel that they have, or are seen by others as having, a sense of connectedness with the wider community. Participants expressed the importance of maintaining social contact and having a sense of connection and belonging to the community.

[90] Sullivan et al (2015, England)	To explore older people's understandings of loneliness.	Study of older adults (N=37) who identified as lonely. Participants were drawn from two previous studies. F, 25; & M, 12; aged 65-87 residing in south or southeast England. The majority were living alone and 14 reported limiting health problems.	In depth interviews. Thematic analysis.	Drawing on Mijuskovic's (2012) theoretical positioning, it is argued that loneliness is difficult for those experiencing it to talk about and make intelligible to others.<br><br>Reviews various models (cognitive and social deficit models; emotional loneliness) and theoretical frameworks (attachment theory, interactionist approach, philosophical perspectives).	1) Fluidity: loneliness fluctuated over the course of the day and over longer periods of time.<br>2) Interaction between various elements in loneliness and coping: intrapersonal characteristics, interpersonal relationships, life events, immediate social environment and wider structural factors.<br>3) Many participants were coping with a combined loss of physical, economic, and social capital and having to routinely access personal resources, albeit sometimes limited, to maintain well-being.<br>4) This could include loss of symbolic capital, and effects of a society that marginalizes older people.<br>5) Importance of the life course: participants continually explained the present in relation to the past.<br>6) Participants emphasized their active social lives as central to their management (& concealment) of loneliness.	Loneliness is not linear as current theories suggest but dynamic and multidimentional. This demands a more integrated theoretical position as in de Jong Gierveld and Tesch-Römer (2012).<br><br>Loneliness is a complex and dynamic experience. Being old and lonely is stigmatizing and difficult to talk about, it is unique to each individual.<br><br>A paradigm shift, including interdisciplinary perspectives, is required to understand loneliness.

[91] Taube (2015, Sweden)	To explore the experience of loneliness among frail older people living at home.	Participants were frail older people (N=12) who were purposively selected from a larger interventional study (RCT) They were aged 68-88 (mean 79 years), F, 10; & M, 2; living alone at home, in need of assistance, able to communicate in Swedish and have reported experiencing loneliness (N= 12).	Semi structured interviews.<br>Thematic analysis of manifest and latent content.	Loneliness is a feeling of being disconnected from other people and society. It is reinforced by societal values of the norm being youth and independence. Older people face barriers including physical and social losses.	The overall theme was 'Being in a Bubble', living in an ongoing world, but excluded because of the participants' social surroundings and the impossibility to regain losses. Loneliness is a constant and incurable state.<br><br>The theme 'Barriers' was interpreted as facing physical, psychological and social barriers for overcoming loneliness. These barriers included the ageing body, fear, the influence of losses, and no-one to share everyday life with.<br><br>A positive co-existing dimension of loneliness, offering independence, was reflected in the theme 'Freedom'. This included having time to reflect and reload, being free to make decisions, being able to create meaningfulness.<br><br>The level of hopelessness the participants experienced was also related to depression: the resemblance in the experiences of loneliness and depression makes it hard to isolate the two phenomena.	This study has shown that the experience of loneliness among frail older people is a struggle in overcoming physical, psychological and social barriers. When not being able to overcome the barriers, there was a sense of hopelessness. There were no expectations that the loneliness would disappear; it was a constant state, albeit not always oppressive. The findings have clinical implications and calls for awareness where the complex situation of being frail is recognized. To promote well-being, a person centred approach, encompassing knowledge regarding frail older people's physical and psychological situation, is suggested when caring for frail older people.<br>With advancing age and frailty, different personal losses are inevitable, which may result in a risk of experiencing loneliness. Thus, facilitating meaningful social connections and activities, and providing support in coping with inevitable losses, could be beneficial for frail older persons. The findings suggest that future strategies for intervening should target the frail older persons' individual

						barriers and promoting the positive coexisting dimension of loneliness.
[92] Tiilikainen (2017, Finland)	To examine emotional loneliness as a lived experience that exists in the form of multiple realities constructed and reconstructed by older people within the context of their different lives and life histories.	Study of older people (N=10) who reported feeling lonely. M, 6; & F,4; aged 70-84, with different backgrounds: widowed, divorced, unmarried or cohabiting, and they lived in different types of rural and urban neighbourhood.	Interviews. Thematic analysis: data driven but with guided by prior theoretical ideas.	Loneliness lacks a clear consensual definition, with concepts of feeling lonely, being alone and living alone often used interchangeably.<br><br>The study draws on Weiss' (1973) conceptualization: emotional isolation refers to the absence of a significant other or someone to turn to. Social isolation relates to loneliness caused by a lack of a sense of belonging or dissatisfaction towards one's social network.<br><br>Emotional loneliness, described as lost and unfulfilled relationships.	Most of the interviewees have faced loneliness that only began in old age, but for some, loneliness has been present for nearly a lifetime.<br><br>The analysis brought up five factors behind emotional loneliness (loss of spouse, lack of companion, longing for a good friend, complex parenthood and troubling childhood experiences) and five factors behind social loneliness (loss of mobility, increased disabilities, individualization, re-location and decrease of social network). After this, the factors were analyzed within three themes: life events, impacts on everyday life and emotional experiences in order to gain understanding of loneliness as a lived experience.	The data show the multifaceted nature of loneliness and its causes. The meaning of lost relationships and the quiet longing for fulfilling ones were manifested as powerful factors behind emotional loneliness in old age.<br><br>Older people referred to loneliness as an inevitable part of ageing, but the cause of loneliness was equivocal. Meaningful relationships have been lost, and new ones have not been found due to increased disabilities or sometimes just not being on the 'same wavelength'.<br><br>Findings emphasize the importance of the quality (not the quantity) of relationships. Loneliness is reinforced in individualistic society, but was reflected as a personal experience embedded in previous life events and present circumstances.<br><br>It would be useful to examine loneliness from a life course perspective.

[93] Topor (2016, Sweden)	To explore the relationship between financial strain and social isolation in people with severe mental illness.	A study of the relationship between experiences of poverty and severe mental illness.<br><br>Participants = 16 people with SMI, M =11; & F= 5. Five of the participants were 34 years old or less and 11 were 35 years+. 11 were living in their own apartment, the other 5 in group homes. 3 of the participants were living with a partner, 1 was divorced and 12 were single. 10 lived in the town and surrounding suburbs and six in the countryside. All were on low incomes.	Grounded theory. Semi structured interviews. Inductive thematic analysis.	Severe Mental Illness has an effect of reducing or changing social networks, although many people with SMI desire to have greater social networks.<br><br>Barriers arising from having limited finances include difficulty inviting people to their home, being unable to attend cultural events, coffee shops or restaurants, and being stigmatized by their old and worn clothing and dental problems.	The overarching theme was "the cost of having friends"; it consisted of five categories: the loss of friends; making do without friends; dependence on friends and family; supported socialisation; and money as an aid to recovery. The participants experienced a connection between their financial circumstances, their social relationships and their lack of initiative in maintaining and developing a social network.	The results underline the importance of considering the person in his/her social context in order to avoid the risk of interpreting rational strategies for coping as psychiatric symptoms. An improved economic situation is not a solution to all cases of social isolation and mental health distress, but it should be included among the various therapeutic resources offered to persons with SMI. The present study is an attempt to understand people in their social contexts and from their own perspectives. In the work to develop evidence-based interventions, users' experiences and preferences are of central importance.
[94] Tuominen & Pirhonen (2019, Finland)	To deepen understanding of the informal social relationships of the oldest old by applying qualitative methods. It considers ideas of the fourth age, socioemotional selectivity theory, and	The data used in this study originate from the Vitality 90+ study carried out in the city of Tampere in southern Finland. It is a multidisciplinary study focusing on longevity and the oldest old. This study utilizes life-story interview data from 2012. Every fifth community-dwelling woman and man living in Tampere (born between the years 1921 and 1922, thus aged 90–91 at the time) was	Qualitative life-story interviews were analyzed using qualitative content analysis.<br><br>The participants were interviewed in their homes. The shortest interview took 34 min and the longest 3 h and 20 min. There were nine interviews which took less than an hour, 24 interviews that took 1–2	The data were analyzed using the inductive approach of qualitative content analysis and typical stages of familarisation and coding.	Company – from neighbours/community and family face to face or telephone and engagement in activities<br>Solitude – can choose it<br>Help – accepting it where needed from family and friends and neighbours - loneliness can be defined by not taking help<br>Emotional Activity - engaging in activity leads to joy/enrichment but loss and grief and poor health define loneliness	We argue that in order to better understand very old age in its complexity, various – and also divergent – perspectives need to be acknowledged. This can best be accomplished by qualitative studies, which allow the oldest old to reveal their perceptions in their own words. Consequently, more studies using a qualitative approach are needed to capture the multiple aspects of social life in the oldest old people.

	gerotranscendence theory from the viewpoint of Finnish community-dwelling nonagenarians.	sent a request to participate in the interview. The request was sent to 99 women and 41 men, of whom 25 and 20, respectively, gave a positive answer. The response rate was 25% for women and 48% for men. The majority of the participants were widowed, lived alone, had no need for help, and rated their health as average.	h, ten interviews which took over 2 h, and two interviews lasting over 3 h. All 45 interviews were tape-recorded and transcribed into 1073 text pages. The interviewers had also documented short details of the interview situation and their personal observations about participants			
[95] van Bergen (2011, Netherlands)	An investigation of ethnic and gender-specific patterns of suicidal behaviour in South Asian-Surinamese, Turkish, and Moroccan immigrant young women	Life History interview with 47 women aged 18 - 40<br><br>Comparison group of Dutch women (N=14) who demonstrated suicidal behaviour or serious suicidal ideation (as indicated by health care staff and/or participants themselves); and were not experiencing a crisis at the time when they were invited for an interview (e.g., suicidal crisis, a psychotic or manic episode).<br><br>Female Migrants (N=33)	Qualitative Life History Interviews 1-3 hours<br><br>Narrative thematic analysis	Loneliness and feeling alone contributory factor to non-fatal suicidal attempts amongst migrant women<br><br>Loneliness is hidden along with other negative emotions to preserve honour and pride in culture, community and family. Appearing to keep family together is central to cultural and gendered identity.	Domains of loneliness are explained as contributory factors to suicide attempts (i) to lack of autonomy, self-worth and value (ii) clash over life choices (husband and wife i.e gendered traditional cultures), (iii) lack of connectedness and affection, (iv) psychiatric illness	Study revealed experiences of disempowerment; unattainable meaningful life. Prevention strategies could include autonomy awareness programs assistance while contesting cultural norms imposed, family counselling programs that enhance mutual relationship building between parents and their children

		South Asian-Surinamese (13) Turkish (10), Moroccan (10),				
[96] van den Berg (2017, Netherlands)	To examine and facilitate social support amongst medical educators to reduce sense of isolation and loneliness in the workplace and create a sense of community at work	Semi-structured interviews with Medical Educators; N=13 (F, 7; M, 8) diverse backgrounds and working circumstances; clinicians and basic scientists to explore social support as a means of alleviating loneliness in the workplace	Qualitative Interviews<br><br>Descriptive phenomenology and thematic analysis	Focuses on defining social support as a job resource provided by emotional and instrumental support in the workplace as a way to reduce isolation and loneliness.	Domains of social support for alleviating loneliness:<br>(i) sources of support and their intent (e.g. a superior with the intent to stimulate personal growth)<br>(ii) the materialisation of support (e.g. sought or offered)<br>(iii) its manifestation (e.g. the act of providing protected time<br>(iv) the overarching effect of social support (practical effects and meaning.<br><br>Receiving support could lead to (i) feeling reassured and confident;<br>(ii) feeling encouraged and determined (iii) a sense of relatedness and acknowledgement of the educator role.	Facilitating social support at work requires attention to the source, manifestation and materialisation of support to reduce sense of isolation and loneliness and contribute to the prevention of burnout, fuel work engagement and improved performance of medical educators
[97] van der Zwet et al (2009, Netherlands)	To explore GPs' experiences with lonely patients and their feelings regarding consultations and resulting behaviours	Semi-structured interviews with Dutch GPs N=20 (M, 18; F, 2)	Qualitative interviews<br><br>'Grounded-theory-like' approach to thematic analysis.	GPs considered loneliness as something subjective, a feeling connected to lack of social relations.<br><br>Highlighted that lonely people consult more often	GPs conceptualised loneliness as (i) unhappiness due to a lack in the number and/or quality of relationships, (ii) loss of contact is from reduced number of social connections (older people); (iii) the quality and character of social interactions, individualistic mindset and macho behaviours (young	Loneliness is subjective and situational; a feeling, which could change over time depending on external factors (e.g. life events) and personality traits (e.g. coping strategies).<br><br>Chronically lonely patients are more likely to evoke negative feelings and behaviour in their

					people) (iv) a situation in which there is a lack of stimuli; an inability to cope; transitory or chronic	GPs. GPs should try to recognize these emotions and make sure they do not harmfully influence consultation.
[98] Vasileiou et al (2017, England)	To examine informal caregivers' reflections on, and accounts of, experiences of loneliness linked to their caregiving situation	Part of a larger mixed-method research project that examines experiences of loneliness. A qualitative interview design with informal caregivers.<br><br>N=16 caregivers. 8 spousal caregivers, 4 daughters caring for a parent, 3 mothers caring for a child (or children), and 1 woman looking after her partner. The cared-for persons were suffering from a range of mental and physical health conditions.<br><br>All caregivers were living with the cared-for person, except for one mother who lived separately from her adult daughter at the time of the interview. Four of the participants were assisted by professional caregivers at home and three regularly accessed respite services. Fourteen caregivers were British (two did not report their nationality).	Qualitative Interviews<br><br>Inductive thematic analysis.	Loneliness defined as "the unpleasant experience that occurs when a network of social relations is deficient in some important way, either quantitatively or qualitatively" (Perlman and Peplau, 1981, p. 31).<br><br>The distressing experience deriving from a discrepancy between one's desired and actual levels of social relations (Perlman and Peplau, 1981)in people whose social relations are likely to alter and be disrupted on account of work situations (i.e., lone and remote working) and major life changes and transitions (i.e., assuming a caregiving role; moving away from home to study).	Loneliness connected to (i) shrunken personal space and diminished social interaction caused by the restrictions imposed by the caregiving role, (ii) relational deprivations and losses and (iii) powerlessness, helplessness, and a sense of sole responsibility. Social encounters were also seen to generate loneliness when they were characterized by some form of distancing i.e not feeling understood and no recognition of the role of the care giver and not having empathy for their role leads to inward loneliness.	Though not all sources or circumstances of loneliness in caregivers are amenable to change, more opportunities for respite care services, as well as a heightened sensibility and social appreciation of caregivers' valued contributions could help caregivers manage some forms of loneliness.

[99] Vasileiou et al (2019, England)	To examine the coping strategies young adults deploy to manage experiences of loneliness whilst studying at University.	A qualitative, cross-sectional study using semi-structured interviews with University students (N= 15) who had moved away from home to study, and who self-identified experiencing loneliness.<br><br>F, 9; M, 6. Median age 20 years old (youngest=18, oldest=29). 9 undergraduates, 6 postgraduates. 7 British, 8 international students (i.e. Spain, Germany [2 students], Mexico, China, South Korea, Vietnam, and India). All students used digital technologies to keep in contact with their family and friends (e.g. Skype, Facebook).	Qualitative Interviews.<br><br>Content Analysis	Loneliness defined as the distressing experience arising from discontent with the degree and quality of social connections (Perlman & Peplau, 1981)	Concealment of loneliness as protective (stigma of loneliness)<br><br>Loneliness domains include feelings of helplessness, needing to escape and submission<br><br>Selective and contextual coping strategies included accommodation, mainly in the form of distraction, support-seeking, social isolation, self-reliance, and problem-solving behaviours	The research illuminates the selective, contextual, and socially-influenced character of coping. Young adults at University do not only strive to manage their loneliness but also to preserve valued social resources, existing and potential. As loneliness is a pervasive experience in this population and has been found to be conducive to psychological distress, any effort to improve students' mental health and well-being and prevent mental health problems requires strategic responses to this challenge from academic institutions.
[100] Walkner et al (2018, USA)	To examine the perspective of community leaders who work with aging women to understand how social isolation affects communication and service provision	Focus groups and interviews conducted with community leaders to examine how agencies and service providers communicate with older people to help them remain socially connected.<br><br>N=307 people participated in 18 focus groups. M, 12; F, 295.	Qualitative interviews and focus groups.<br><br>Key word analysis (NVivo)	Addresses social isolation but links to strategies also identified in loneliness literature.	Reducing social isolation through<br><br>(i) supporting independence<br>Iii) developing communication strategies<br>(iii) improving communication and service provision (e.g. mentorship, buddying befriending across generations)	Social isolation is a problem facing many older women. Being isolated (i.e. losing members of a social network) leads to loneliness which least to poor health. Improving communication and social support through a range of print, oral, and digital forms and developing communication and support plans is required.

[101] Warren (1992, USA)	To present a concept analysis of social isolation (and associated terms)	A theoretical concept analysis;<br><br>(1) identifying the concept; (2) stating the aims of the analysis; (3) examining existing definitions of the concept; (3) constructing cases; (4) testing the cases; and (5) formulating criteria of the concept (Chinn & Jacobs, 1987).	Conceptual Analysis	Social Isolation "the state in which the individual or group expresses a need or desire for contact with others but is unable to make that contact" (Carpenito, 1992, p. 731).<br><br>Reisman (1973) and Weiss (1973): social isolation results from the lack of supportive and meaningful network of emotional involvements.	Subsets of Social Isolation<br><br>Alienation -a ". . continuum process in which a person is separated from other persons, is unattached to other persons, and exhibits few bonds or ties of an enduring or intimate nature" (Urich, 1977, p. 7). Causes affected persons to experience feelings of disconnection, separateness, loss of self, depersonalization, rootlessness, and a lack of community<br><br>Loneliness is described as an unwanted, uncomfortable feeling that occurs when a person lacks meaningful social relationships (Perlman & Peplau, 1982; Tanner, 1973). A lonely person feels socially isolated and disconnected to other members within society.	Use model case and contrary case to assess risk and development strategies to address social isolation in nursing<br><br>MODEL CASE = stigmatized environment, societal indifferent, personal-societal disconnection, personal powerlessness<br><br>CONTRARY = none of the criteria of social isolation present within life: no stigma, loneliness, rejection, alienation, or powerlessness. Fully socially supported
[102] Winterstein & Eisikovits (2005, Israel)	To describe and analyse the ways in which loneliness becomes the central existential theme in the experience of aging battered* women.	In-depth interviews with aging women who lived with violence for a significant portion of their lives. Focuses on loneliness, which appears to constitute the central theme of their existential experience.	Life History interviews.<br><br>Phenomenological approach to lived experience.<br><br>Content analysis	Loneliness as subjective (Rook, 1984; Rokach, 1988).<br><br>Multi-faceted, encompassing both experiential and emotional aspects (Gibson, 2000)	Cultural and gender specificity of loneliness<br>(i) loneliness in Couplehood – through matchmaking – culturally specific;<br>(ii) loneliness in parenthood – complex interplay of meaning but self-sacrifice through staying in the relationship for the children;	Loneliness is experienced on all ecological levels, including the self, the family of origin, the violent partner, the children, and the extended family. It also permeates the social relationships. The combination of loneliness, violence, and old age creates suffering that colours everyday life and becomes not only the constant

	**Battered is the term used by the authors of this study.*	N=21 old 'battered' Jewish women in Northern Israel. 35—85 yeas<br><br>13 living with their partners, 8 separated but not divorced. Diverse ethnic origins and levels of education. 7 worked as professionals in various occupations nurse, childcare worker, freelance decorator, piano teacher, X-ray technician, secretary and kindergarten teacher), 3 were part-time cleaning women, and 11 were housekeepers. Two interviewees were orthodox religious; about half indicated holding traditional beliefs but not observing strictly religious lifestyles; the others reported being secular. The interviewees (except one who had no children) had between 1 - 10 children, all above the age of 35, and all but one were grandmothers.		Loneliness as a state of mind based on the inner-feeling of abandonment, rejection and being erased from the other's consciousness.<br><br>Phenomenological structure of loneliness as self-alienation, self-avoidance, emotional instability and negativity, vulnerability (Rokach, 1988)	(iii) loneliness in a social context and in family through need to keep the violence hidden	background against which life unfolds but also the governing variable in their experience.<br><br>A narrative approach enables the therapist to help old battered women to reframe the experience of loneliness and suffering in a manner in which they can attach strength and competence to the remaining part of their lives.
[103] Wong et al (2017, Hong Kong)	To examine ageing and loneliness in Non-Western urban environment in	Thirty-seven community-dwelling, Chinese adults aged 65+ were interviewed in focus groups and their accounts analysed and interpreted using a	Qualitative Interviews.<br><br>Phenomenological<br><br>IPA	Identifies with Victor et al.'s (2005) 4 main theoretical Perspectives.<br><br>1. Cognitive theory	Perceived insufficient care for older people, a growing distance between themselves and society, and their disintegrating identity in	Rather than leaving the elderly to withdraw to a passive lifestyle and feel uneasy, vulnerable, helpless and angry about their current situation, findings from this study

	non-Western work and to reconceptualise loneliness by exploring older people's experience of alienation at the societal level.	phenomenological approach. Data were collected from four focus groups that comprised of 37 older people, M, 15; F, 22, across two different age groups (aged 65 to 79 and aged 80+.		2. Psychodynamic theory<br>3. Interactionist theory<br>4. Existential theory<br><br>Going beyond loneliness as interpersonal to consider societal and sociocultural.	society to be primary sources of societal alienation.<br><br>In response, older people adopted a more passive lifestyle, attributed marginalisation and inequality to old age, and developed negative feelings including unease towards ageing, vulnerability and helplessness, and anger.	suggest that loneliness may be reduced by addressing the identified sources of societal alienation and increasing the support and empowerment of older people in society so that they can proactively engage in improving quality of life for themselves and for all older people.
[104] Zumaeta (2019, Chile)	To examine the socioemotional costs of being a high-ranking leader in a corporate context.	Fourteen Latin American managers from the researcher's personal and professional network were interviewed (11 senior managers and 3 middle managers). The senior managers interviewed ranged in age from 38 - 60 years (*M* = 47 years), with more than 70% of the sample having more than 20 years of professional experience. Middle managers' ages ranged from 30 - 43 years (*M* = 35 years). Almost all participants were male (*M, 12*). All the participants worked in Chile, and all but two were nationals of that country. The majority of participants (*N* = 11)	Qualitative Interviews<br><br>Thematic Analysis	Loneliness defined as "an individual's subjective perception of deficiencies in his or her social relationships." (Russell, Cutrona, Rose, & Yurko, 1984, p. 1313).	Top executives are more prone to be lonely due to the *pressures* of the role: increased social distance, lack of social support, and exhaustion related to the role. Two ongoing *conflicts* in the position (Role vs. Person and Distance vs. Closeness) and four coping *strategies* were identified (mental and physical disconnection, healthy lifestyle, support from one's network, and affecting and influencing others). Finally, the research revealed that some organizational conditions could also increase the risk of loneliness at the top (e.g., high–power distance culture) Thematic network diagram for loneliness included.	Senior managers are more prone to be lonely inside and outside work due to the demands of the role. The extent to which they feel lonely is dependent on how they manage two ongoing conflicts in the position and the coping strategies used. There are organizational conditions that could increase the risk of loneliness at the top. This study provides an empirical foundation to manage top executives' behaviour to reduce feelings of loneliness and enhance well-being.

		worked in the private sector.				
[105] Chile et al (2014, New Zealand; MM).	To examine the significance of social isolation and factors that create it for residents of inner-city high-rise apartment communities.	Derived from a larger study examining concepts of community and connectedness for inner-city residents in Auckland, NZ. Stratified random sampling identified participants for survey questionnaire. Respondents then invited to participate in semi structured interview (N=30) or focus group (N = unclear). Further stratified sampling selected participants for these, to reflect demographic profile of city (age and ethnicity). Focus groups included participants not already interviewed, samples reflected city's demographic profile.	Multi-stage mixed methods. Survey questionnaires supplemented by semi-structured interviews and focus groups.<br><br>Thematic analysis.	Used and extended Wilson's definition of social isolation as 'the lack of contact or sustained interaction with individuals and institutions' (Wilson, 1987, p60).<br><br>Suggest that social isolation is both physical and psychological distance between individuals and community members, and that it can be identified in terms of<br>(1) structural social isolation<br>(2) functional social isolation.	Quant and qual analysis reported together in exploring ideas of functional and structural isolation. Themes based on recurrent common topics and variations in respondents' perspectives on concepts such as social isolation, social connectedness, sense of community, and belonging.<br><br>No specific conceptualisation of loneliness.	Identified two forms of social isolation (functional and structural) and found that 'living alone' or structural social isolation does not necessarily lead to functional social isolation.<br><br>Suggest a need to enhance opportunities for social connectedness that can reduce social isolation. 'Social connectedness reduces loneliness and feelings of isolation which may result in serious psychosocial and other associated health issues' (p162).<br><br>Suggest interventions that might support greater social connectedness, including friendship clubs and other group activities.
[106] Dong et al (2011, USA, MM)	To investigate the cultural understandings of loneliness, identify the contexts of loneliness, and to examine its effect on the health and	Community-based participatory research, a mixed method study with survey questionnaires and structured focus group methods.<br><br>78 community-dwelling older Chinese people in Chicago Chinatown.	Qualitative analysis of focus group sessions.	"Loneliness is one of the main indicators of well-being. It is manifested by intense feelings of emptiness, abandonment, and forlornness (Meis, 1985).<br><br>The insufficient quality or quantity of an	Loneliness was frequently identified among Chinese adults, shown in both emotional isolation and social isolation.	"Qualitative findings fill in the void in aging minority loneliness literature by confirming loneliness and its relationship to the cultural importance of intergenerational relationships and furthermore provides unique window on understanding loneliness among older adults that lays the

	well-being of U.S. Chinese older adults.			individual's network of social relationships is closely linked to the cause of loneliness (Peplau and Perlman, 1982).<br><br>Older adults are particularly vulnerable to loneliness due to the increase of multiple losses, changes, and transitions in later life (Ryan and Patterson, 1987; Donaldson and Watson, 1996).		groundwork for future research on the well-being of Chinese aging population", p. 157.<br><br>Author's "conclude that loneliness is common, yet understudied among Chinese older adults in the U.S. Investigating how U.S. Chinese older adults understand and perceive loneliness is a critical step in promoting the health and well-being of this increasingly growing population", p. 158 .
[107] Finlay et al (2018, USA; Mixed Methods [MM])	To characterise personal and neighbourhood contextual influences on social isolation and loneliness among older adults in the Minneapolis metropolitan area.	In-depth interviews conducted in participants' homes or nearby public space by researcher and research assistant. Participants asked whether they felt lonely or isolated. Follow-up questions probed for multidimensional definitions of quality and quantity of social engagement.<br><br>Data collected in 3 socioeconomically diverse case study areas of Minneapolis metropolitan area. Nonprobability sampling used to recruit	Parallel convergent mixed methods analytical design (Cresswell, 2015).<br><br>Theoretical framework from discipline of health geography – 'relational' approach to space and place.<br><br>Qual data organised in NVivo 11 and analysed thematically. Secondary analysis (Braun and Clarke 2006) to examine social isolation and loneliness	Social isolation defined as a measurable lack of social relationships.<br><br>Loneliness as an 'affective state reflecting the subjective experience of feeling alone or lonely (Hawkley & Cacioppo, 2007; Klinenberg, 2016, Steptoe et al, 2013).<br><br>Authors note association of both constructs with range of health outcomes and risks, and their attribution to contextual personal, social and cultural factors.	Identified 6 overarching, interrelated categories relating to why participants did or did not feel lonely or socially isolated:<br>(1) *Physical and mental health* – limiting or boosting opportunities to socialise<br>(2) *Personal preference* – preference for or against solitary activities or social circle, anti-social attitudes or behaviour<br>(3) *Sense of aloneness* – lack of daily contact, fear of dying alone, death of family and friends, challenge of making new friends<br>(4) *Safe spaces* – lack of residential safety, transitory	There are many intersections between older people and neighbourhood contexts which influence their daily experiences of social isolation and loneliness.<br><br>Social isolation and loneliness overlapping but distinct constructs in both quant and qual. Participants often conflated the two in interviews.<br><br>Participants challenged conventional definitions of social wellbeing, for example, highlighting personal agency in decision-making about being alone.

		convenience sample (N=124) of participants.<br><br>Criteria for inclusion were: age 55+, not in a care setting, residing in case study areas, cognitive capacity.<br><br>Age range 55-92, 69% female<br>57% white, 25% Black / African American, 18% other, Professional/managerial 41%, other 59%.		References framework of neighbourhood social capital (Carpiano, 2006). Defines social capital with ref to Bourdieu (1986) as 'aggregate of actual or potential resources linked to possession of a durable network of institutionalized relationships' (p26).	and insecure lifestyle patterns<br>(5) *Sense of community* – racial inclusiveness, multigenerational spaces, shared public space, housing characteristics<br>(6) *Services and amenities* – recreational sites available, local services, lack of sites in which to gather<br><br>Living alone strongly associated with social isolation and loneliness, although qual showed that for some participants strong personal preference outweighed this.	Authors highlight that the 'roles of personal agency, resilience, and ability to self-determine social isolation and feelings of loneliness have been emphasized in theoretical work, yet are often ignored in empirical studies.' (Hawkley and Cacioppo, 2010; Harris, 2008).<br><br>Context matters - consistent with Carpiano (2006) framework of neighbourhood social capital.<br><br>Results confirm Cloutier-Fisher and Kobayashi (2009) portrait of 'social vulnerability', demonstrating that individual characteristics are embedded within broader contextual variables (eg a park is a lovely place for one, but a feared site for another).
[108] Heinz (2018, Canada; MM)	To primarily extend previous work on transgender communication dynamics.	This mixed-methods study draws on quantitative and qualitative data on interpersonal communication measures and experiences of Canadian transgender people under the framework of Meyer's minority stress model.	Mixed Methods.<br><br>A qualitative analysis of the overall data (i.e., the text of the answers to all the questions) employed a traditional thematic analysis.	Transgender needs assessments, traditionally conducted from a public health perspective, tend to point to common experiences or perceptions of social isolation, loneliness, and the perception and/or experience of social exclusion as a result of	Isolation: Nineteen participants made references to feeling alone, lonely, or socially isolated in their responses. In some instances, participants reported social anxiety exacerbating isolation; such an anxiety was attributed to negative experiences or expectations of negative encounters. Participants described being isolated or	These results offer a glimpse into the ways in which a sense of real or perceived social isolation and loneliness might interact with communication behaviors and dispositions and vice versa.

		44 participants between the ages of 19 and 65 years (Mage = 33.27 years)		stigma and discrimination, particularly among transgender populations disproportionately affected by intersecting factors such as poverty, HIV status, race, class, culture, migration status, or ability status	isolating themselves by avoiding communication encounters, deflecting personal questions, restricting open communication to close or known others, and opting for silence. Participant 3 wrote, "I keep people at arm's length."	
[109] Hinojosa et al (2011, USA, MM)	To explore perceptions of the relationship between social isolation, depressive symptomatology and the ability to manage activities of daily living post-stroke for white, African American, and Puerto Rican veterans, using post-stroke narratives.	Study examines post-stroke isolation.<br><br>N=77 male veterans: 18 African Americans, 29 Puerto Rican Hispanics, and 30 non-Hispanic whites.	Mixed methods. Face-to-face interviews alongside quant self-report measures of recovery & functioning after discharge.<br><br>Grounded theory. Thematic coding. Matrix to categorise participants into 4 levels of connectedness & social isolation.	Social isolation defined as lacking social access to others, a perceived lack of social support from others, a persistent sense of one's physical and social disconnection from others and the community, an inability to contribute to the family or the community in personally meaningful ways, and the inability to engage in physically intimate relations.<br><br>Social isolation linked to loneliness and increased risks of poor physical and mental health.	Characteristics of connectedness are:<br>1. Availability of others<br>2. Perceptions of support from others<br>3. Ability to contribute<br>4. Having an intimate relationship<br><br>Characteristics of isolation are:<br>1. Unavailability of others<br>2. Perceptions of lack of support from others<br>3. Inability to contribute<br>Not having an intimate relationship	Social isolation was evenly distributed among participants, the numbers ranged from 1/3 to over 1/2 falling into the socially isolated category. Greater family involvement in post-stroke care may improve outcomes. Decreased social isolation is related to decreased depressive symptomatology and increased ability to perform IADLs. Families may need more education regarding maintaining connections and providing support to reduce social isolation. Practitioners can recommend social services, local support groups, and other means of achieving higher levels of social support from the local community.
[110] Parigi & Henson (2014, USA)	Offers a new measure for social isolation for contemporary American	A review and introduction to development of a conceptual framework for development of a new measure for contemporary social isolation. In	Reviews previous research on social isolation. Highlights division in literature between researchers who conceptualise	Instead of asking does new social media make Americans more isolated, should we be saying that, with Sherry Turkle (2011, p11)	Focuses on social isolation in contemporary America with regard to developments in technology and social media.	Difficulties in measuring social isolation have stemmed partly from approaching it through a structural definition. Is it objective or subjective? The author suggests that 'meaning-

	society. Contributes to thinking about concepts of loneliness	particular in relation to new social media.	social media as creating more feelings of isolation, and those who do not. Final section of the paper offers novel conceptual framework for studying social isolation.	"Technology reshapes the landscape of our emotional lives, but is it offering us the lives we want to lead?"<br><br>Social ties are important because they generate meanings for two people that share connection (White 1995a, White 1995b). However, keeping disparate *meanings* together, i.e., ties from diverse social contexts, can create a dissonance (Bearman & Moody, 2004) that produces feelings of loneliness' (p4).<br><br>To assess the current state of social isolation, we need new measures created by combining two perspectives on isolation, namely, those that focus on (1) structural characteristics of individual networks (2) subjective feelings	Identifies debate about relationship between social isolation to fragmentation of society, but suggests that a more fragmented society does not necessarily imply the creation of more social isolation.<br><br>Reviews literature on social isolation in contemporary society, divided between:<br>- Those that see isolation as a (negative) by product of modernity<br>- Those that see it as a structural position that can produce both positive and negative effects	based measures' of structural isolation are potentially more useful than measures based on counts of ties between individuals. Connects to subjective definitions of loneliness especially in regard to emotional loneliness in a sociological framework interconnecting social isolation and loneliness.<br><br>'We follow Harrison White and see ties as stories, ie the subjective representation of objective relationships' (1995a, 1995b)<br><br>Offers conceptual framework 'for studying isolation which includes simultaneous consideration of objective and subjective isolation<br><br>'we see social isolation as *a ratio of non-overlapping contexts to the average time spent per relationship*' (p20)
[111] Rew (2002, USA; MM)	To describe relationships of sexual abuse, social connectedness,	Convenience sample of 96 homeless adolescents were recruited through a community outreach project in central Texas. A	Mixed methods. Survey data, focus group and individual interviews.	Traumatic childhood sexual abuse can result in feelings of social disconnection and difficulty in establishing	Themes explored in qual data:<br><br>Perceptions of social connectedness and loneliness associated with being homeless.	High levels of loneliness among participants.<br><br>Those with histories of sexual abuse perceive themselves to be

	and loneliness with perceived well-being (health status) among homeless adolescents. To explore these perceptions through focus groups and interviews as a means of providing a context for interpreting empirical data.	sub-sample (N=32, aged 16-23) attended one of four focus groups to discuss experiences of homelessness, feelings of loneliness and factors related to perceived health status. Of this sub-sample, a further group (n=10) were interviewed individually in depth. Interviews audio-recorded and transcribed.	Qual data analysed using manifest and latent content analysis procedures (Holsti, 1961; Patton, 1990).<br><br>Statements and phrases coded to match key questions: (1) feelings of loneliness and disconnection related to living on the street (2) current health compared to prior health (3) factors influencing current state of health (4) factors impeding current state of health (5) what is need to thrive on the streets (6) chances for long and healthy life.	and maintaining relationships.<br><br>Experiences of abuse lead to feelings of inadequate social support and loneliness (Goodman and Berecochea, 1994).<br><br>Social disconnectedness or isolation in homeless youth is reinforced if drop out of school, rejected or ignored by peers. 'Such rejection and isolation may lead to feelings of loneliness' (p52)<br><br>References Weiss (1974) and Rook (1984) and Krauss et al (1993).	Positive and negative, and strategies for managing or coping.<br><br>Perceptions of wellbeing and health status related to being homeless	less socially connected, more lonely and less healthy than those who are not homeless.<br><br>Social connectedness inversely related to loneliness and positively related to well-being.
[112] Sawir et al (2008, Australia, MM)	To examine experiences of loneliness in international students in Australian universities.	Study of loneliness in international students (N=200) in nine HE institutions in Australia. F, 101; & M, 99, mostly from Asian nations.	Mixed methods, structured interviews.<br><br>Deductive analysis starts with a theory of loneliness, themes are not emergent, data are reported mostly in terms of percentages.	Cultural loneliness emerged as an important topic.<br><br>This is an additional category to those of emotional (personal) loneliness and social loneliness.<br><br>For international students, like other migrants, loneliness can	First experiences included the sense of being lost and in a strange place, uncertain about what to do and facing settlement problems, e.g. housing, money.<br><br>Personal loneliness was caused by the absence of intimate persons and lack of cultural fit. Social loneliness was caused by barriers to social networking and financial constraints.	Loneliness is better understood when cultural variations and intercultural settings are taken into account.. Cultural loneliness is triggered by the absence of the preferred cultural and/or linguistic environment. The propensity to cultural loneliness explains why some students in this study, who have apparently adequate access to social networks and good personal back up, still

				arise from a sense of loss and isolation as well as anxiety, confusion and disappointed expectations.<br><br>International students may experience an extreme version of social loneliness due to missing their own cultural and linguistic setting.	Only a few linked loneliness to personal attributes. Loneliness was compounded by problematic institutional relations and lack of support.<br><br>Three types of coping strategies are identified: (a) personal strategies; (b) seeking to augment social relationships or the capacity to engage in them; (c) strategies of seeking help, often of a professional or institutional kind.	report a continuing loneliness. The distinction between individualist and collectivist cultures is also a powerful explanatory factor regarding loneliness. Students from collectivist cultures may struggle with adjusting to the expectations of individualist ones.
[113] Tahir et al (2017, Malaysia; MM)	To explore the experience of isolation among head teachers in the early stages of their career and to identify strategies used to overcome it.	Sample of head teachers (N=10) with less than 3 years experience interviewed in depth for reflections on causes, experiences and strategies for managing feelings of isolation. No demographic characteristics reported.	Mixed methods, sequential exploratory design. Questionnaire, followed by interviews.<br><br>No analysis methods reported for qualitative data.	In leadership context isolation may be perceived as a professional necessity (Abrams, 1997). It is defined in terms of 'professional isolation in which head teachers are deprived of support, good relationships and companionship with others in the workplace, which in turn causes concern and anxiety' (p165)<br><br>Isolation is 'a lonely feeling' (p165) that can lead to suffering related to stress and can affect quality of work.	Authors report on perceived causes for isolation and strategies for coping with it.	Levels of professional isolation are reported to be low and when they do occur, short term.

				Also references processes of organisational and personal 'socialisation'. Author tends to bracket 'isolation and loneliness' together.		
[114] de Jong Gierveld et al (2018, USA)	To investigate ways of theorizing and conducting research in the field of loneliness and social isolation.	Examines concepts of loneliness and social isolation, the measuring instruments and the prevalence of loneliness. The chapter continues by an overview of theoretical ideas regarding loneliness, focusing on individual level and societal predisposing characteristics as well as on genetic/evolutionary perspectives on the onset and continuation of loneliness.	Conceptual Overview	Several types of loneliness are distinguished:<br>1. the positive type and concerns the voluntary withdrawal from social contacts and is oriented toward goals such as reflection, meditation, dedication towards writing or painting and communication with God.<br><br>2. combines negative and positive facets. Moustakas (1961, 2012) describes this existential loneliness type as an inevitable part of the human life itself, involving self-confrontation in periods of crisis, an inner process of doubt and uncertainty, feeling totally isolated even if supportive network members are available. At the end this situation	Although personal, loneliness is not an exclusively individual phenomenon but embedded in given forms of social organization and cultural fabrics.<br><br>People's relationship expectations or standards are shaped by the normative climate in which they find themselves. Cultural norms and values can be conducive to loneliness. Norms and values affect people's ideas about the optimal size of the network, and the obligations and duties of family members. Living alone generally gives rise to loneliness, but this is the more so in countries where older adults without a partner are expected to live with their families (e.g., Greece, Italy) and the less so in countries where older adults without a partner prefer to live alone (e.g., Finland).	Future research should address the possibility of changing patterns of social integration and loneliness, as related to characteristics of members of successive birth cohorts.<br><br>An increasing flow of work from disciplines such as psychology, sociology, and epidemiology has broadened the understanding of the mechanisms behind the onset and continuation of loneliness. The review also lead to some additional observations: nowadays we see (a) more longitudinal research based on high quality loneliness measuring instruments; (b) more international comparative research investigating cultural values and norms affecting country level differences in loneliness; and (c) more research that no longer stems exclusively from the US, Canada, Western and Northern Europe, but increasingly address the antecedents and

				provides an avenue for self-growth, power and inspiration.<br><br>3. unpleasant or inadmissible lack of personal relationships.		consequences of loneliness in Central and Eastern Europe, Asia, and even Africa.
[115] Gedvilaite-Kordusiene (2018, Lithuania)	The chapter looks to analyse the feelings of loneliness in a vulnerable group in society: elderly parents who belong to Lithuanian transnational families and live in elderly care institutions.	Drawing on a postdoctoral research project, this study reveals the ways narratives of loneliness manifest in the life stories of older parents from transnational families from Eastern European societies.<br><br>20 interviews with elderly parents. F, 17; M, 3. All aged over 60 and with at least 1 migrant child.	Qualitative book chapter.<br><br>Social constructivist approach to reveal how the members of transnational families construct narratives of loneliness.	Victor, Scambler and Bond's (2008, p.38) interpretation of loneliness as "subjective lived experiences that exist in the form of multiple realities constructed and reconstructed by individual older people within the context of their different lives and life history".	Where loneliness is not explicitly named, it is implied by expressions such as "grey days are passing by" or "every day is the same" or by interviewees specifying a lack of contacts. In other cases, it was named and often accompanied by tears.<br><br>Loneliness was expressed in two groups: One being from sources associated with the migration of children and another associated with other life course events: losing one's home and experiencing smaller social networks.	Loneliness, in many cases does not appear to be a natural part of human existence, but rather just another part in the puzzle of unhappiness shaped by social and economic forces. According to the findings in this study - Loneliness in old age is a context-sensitive phenomenon. And, even as the world becomes more interconnected, elderly parents have limited agency to cope with or mitigate feelings of loneliness.<br><br>Key quotes: Elena said " Yes, I do get lonely, and I can admit that I am a lonely person. If all the family is together, it is such a joy, especially if you have daughters; it so precious. But nothing can be done. If life goes on like this, nothing can be done."<br><br>"Only unhappy people live here. I'm unhappy. Could I be happy? There are only unhappy and lonely people here…"

[116]Lake (1986, UK)	Overcoming common problems series, Loneliness – why it happens and how to overcome it. A self-help book underpinned by theoretical framework	A self-help book underpinned by theoretical framework: explaining loneliness and identifying how to cope and alleviate it.	Theoretical narrative and research from survey	Loneliness is a feeling, a subjective experience that can be felt across the life course and results from a lack of meaningful relationships with others and ineffective communication in social relationships. A progressive illness	Stages of loneliness (i) circumstance, (ii) personality (iii) effects<br><br>Communication shapes development of loneliness<br><br>Shyness and lack of confidence attributed to loneliness<br><br>Key to alleviating loneliness is the development of meaningful, stable relationships in which people are valued and understood and can relate to people including friendship, love and respect<br><br>Life course effects of transition periods e.g. adolescence, loss of partner, poor marriages and some gender effects.	The best answer to loneliness is love. Love is the only way to set a permanent value on people. Love values everything people are no everything they do.<br><br>The second best answer to loneliness is coping but this is control mechanism that limits the love we can give and receive and therefore cannot fully alleviate loneliness.
[117] Rook (1984, USA)	To provide an overview of existing interventions pertinent to loneliness and to discuss issues involved in the design and implementation	Overview of theories/strageies.	Review and guideline recommendations.	Emphasises loneliness resulting from the loss or lack of important social relationships rather than loneliness that reflects a dissatisfaction with ongoing relationships.	Identifies a range of approaches for interventions addressing loneliness including:<br>- individual approaches and therapies<br>- Group approaches<br>- environmental approaches<br><br>Discusses approaches to evaluation, including use of	Produces brief summary guidelines for design and implementation of loneliness interventions.<br><br>Notes ethical issues resulting from over-eagerness to intervene and suggests that existing cultural values may pose constraints on effectiveness.

	of loneliness interventions				contextually appropriate outcome measures.<br><br>Discusses design and implementation of interventions and suggests key issues including: targeting, linkages with service providers, stigma, chronic v situational loneliness, social v emotional, alternatives to social relationships, overlap of loneliness and depression	
[118] Batsleer et al (2018, England – grey literature [GL])	The project had two main aims: One, to locate the voice of young people in discussions of youth loneliness; Two, to provide young people, and those that work with them, with the knowledge and insight to help them navigate unwanted and problematic loneliness.	Three phases to the project, run by 42nd Street, a Manchester-based charity: (1) developing capacity of the co-researchers (2) collection and analysis of data (3) immersive theatre performance sharing the project's findings.<br><br>Project targeted at young people who are lonely, aged 10-25 years (themes extracted relate to those aged 16+).	Creative and collaborative co-research project.	Explores the social conditions and experiences of youth loneliness as expressed by project participants.<br><br>Key quotes from participants:<br>"Loneliness means something different to everyone because everyone experiences things differently. But I don't think people should be afraid of loneliness." (Patience, Manchester, aged 20)<br><br>"If you asked me what represents my feeling of loneliness most, it's when I've been in all weekend on my own	Change and transition may impact on a young person's ability to connect and may lead to loneliness.<br><br>Whether sexual, racial or cultural, difference can create a sense of isolation.<br><br>Poverty can impact on a young person's ability to participate and feel they belong.<br><br>Social media present additional pressures on young people but also<br>offer the possibility of connection and positive relationships.	The idea that we are living amongst an 'epidemic', 'social plague' or 'silent plague' of loneliness is common. Important to recognise that conceptualising loneliness as a plague and contagion reflects the prominence of psychology and social psychology in discussion of youth loneliness.<br><br>We are unlikely to arrive at a single definition of what young people think loneliness is. The authors / co-researchers suggest some key messages:<br><br>"Loneliness... it's when you don't feel connected. You're there, but<br>you're not really there in your head."

				and there's left over pizza in the fridge at the end of the weekend, because I've ordered a pizza but I can't eat it all." (Clayton, Manchester, aged 25)<br><br>"Is there a word for 'hanging out with a lot of people you don't really like, just because if you don't you won't have anyone else to be with?" (John, Manchester, aged 25)		"It can really hurt. It has so much effect on your body as well. It can make you really, really not trust anyone"<br><br>"I'm not sure I can say what loneliness is, define it... but ... but I didn't realise before [the project] that it connects to so many different things, you can find it everywhere, in films, in music, talking to friends."
119 Brown (2019, England GL)	The aim of the report is to present findings from the evaluation of the initial Local History Cafe programme to capture the experiences of beneficiaries who have attended the programme and the views of those running and organising the	An evaluation of a previous intervention of a local history café programme that aimed to:<br><br>Tackle social isolation and loneliness amongst over 50s through a quality heritage and wellbeing programme.<br><br>Support heritage and community organisations to learn new skills and use existing skills, experience and assets to tackle these issues.	Mixed methods, but only the qualitative data is used here.<br><br>A thematic analysis was undertaken where the data is analysed to search for themes and trends (Braun and Clarke 2006). This analytical approach was iterative and inductive, building themes upwards from the views of participants and stakeholders. The research team worked together feeding back findings and	Loneliness may result from lack of friendships or peer contact (*Social Loneliness*). This explains why some loneliness scores rise e.g. if an expectation of meeting new friends isn't realised or if the experiences of loneliness are emotional rather than social in nature.	Themes:<br>1.local History Cafes helped with the 'first steps: welcoming, safe spaces offering a social group to the lonely and isolated. People could gradually engage feeling like a 'social being'.<br><br>Participants could attend due to an interest in a talk, thus removing barriers to access by 'hiding' issues of loneliness, and the stigma attached to it. The talks also provided them with 'something to say', feeling like they could participate in conversations.	Those older people who attend the Cafes tend to be white/British women, who are better educated, in good health and report a good quality of life, characteristics not associated with groups who are at risk of loneliness. However, the Cafes also attracted older people with characteristics of living alone and were widowed and divorced. The focus on history and learning within the Cafes may arguably be more appealing to those with higher levels of education rather than those who are disadvantaged and most at risk of loneliness. However, the specific feature of

	Cafés. In doing so, it considers factors that both help and hinders the effectiveness of Cafes for supporting people with loneliness and social isolation, and how Local History Cafes contribute to the local community.	Apply an asset-based, partnership approach, supporting collaboration across sectors working on social isolation and loneliness.<br><br>The evaluation, from December 2017 to July 2019, is based on 40 pre- and post-surveys with participants who attended the Local History Cafes, ten face-to-face interviews with participants, a focus group with participants and four telephone interviews with stakeholders (both paid employees and unpaid volunteers) who were organising and running Local History Cafes. Documentary evidence from Cafe diaries and Tumblr were also used (localhistorycafe.tumblr.com).<br><br>n = 10) interview participants (F-7; M-3), all White, British heterosexual, with a mixture of marital status.	continually updating thinking as data was emerging.		2.Local History Cafes were inclusive spaces: where participants could actively contribute and have a purpose. The 'social aspect' offered the potential to create a sense of self-fulfilment and agency. Knowing more about your local area could "enlighten" a person, and "widen knowledge", which gives purpose and meaning against the backdrop of uncertainty and change associated with older age.<br>The documenting of local history and sharing of life stories enabled some to create a positive sense of self, a recognition of the importance of "passing on" their memories and stories and in doing so, being involved, socially, and keeping the mind occupied. The sessions were inclusive, across ages, and this inter-generational aspect meant people felt like-minded people could come together.<br>3.The 'local' was positive for making sustainable connections: It led to "recognising what is good about what is on your doorstep" and provided the opportunity for incidental meetings or 'bumping' into	Local History Cafe is the combination of social interaction and education. Traditional education formats often see people attending but not socialising e.g. once the talk is complete, people leave. Local History Cafes subvert this tendency and put social interaction driven by a passion for local history and heritage centre stage. This can have a wider appeal. Local History Cafes, therefore, have the potential to help all older people even for those who may not be lonely and socially isolated, to extend their social networks and so prevent future social loneliness and social isolation.

					people to become deeper, with one calling it the ripple effect where these social connections were promoted beyond the café's existence.<br>4. Barriers to change:<br>Difficult to keep it local as a diverse range of locations used; limited sessions meant it was difficult to meet new people and the session dynamics needed to be managed carefully in terms of the mix of gender and dominating participants; and the cafes were not attracting the hard to reach or most lonely as those who attended were not perceived as lonely, but they perhaps did not display visible signs of this.	
[120] Essex (2010, England - GL)	To understand the lived experiences of parents who have been identified as being socially isolated and had taken part in the Time Together home visiting intervention which was aimed at improving the	Doctoral dissertation. University of East London.<br><br>A qualitative study was carried out to understand the experiences of N=7 parents who had taken part in the UK based Time Together home visiting intervention. All parents had been referred due to concerns about the parent being socially isolated and/or having difficulties in their relationship with their child. All parents	Qualitative. Interviews.<br><br>Interpretative Phenomenological Analysis (IPA). Social constructionist framework.	Social isolation as a feature of loneliness.	Parents experienced a change within their notion of self as a result of the intervention. The relationship with the home visitor often empowered parents. Social isolation was seen as a self-devised strategy used to limit social interactions that evoked feelings of fear.<br><br>Parents' social isolation was complex and influenced by adverse life factors. Often used as a protective strategy - making them feel more in control.	Socially isolated parents experiencing difficulties within the parent-child relationship can be supported to become more involved with their community.<br><br>Parents themselves need to have their needs for connection met to avoid being lonely.

	parent-child relationship.	were interviewed after they had taken part.				
[121] Huijbers (2018, England - GL).	To understand the views and experiences of people using mental health services in Kensington and Chelsea and Westminster in relation to loneliness and social isolation and what might help to prevent or reduce loneliness.	The study involved N=56 participants with experience of secondary mental health services, aged 18-75+, living in Kensington and Chelsea and Westminster, London. Interviews were structured, face to face and conducted by researchers from the User Focused Monitoring project.	Mixed methods study. Qualitative structured interviews.<br><br>Thematic analysis.	Loneliness is 'a subjective, unwelcome feeling of lack or loss of companionship. It happens when we have a mismatch between the quantity and quality of social relationships that we have, and those that we want'. (Perlman and Peplau, 1981). (p5)<br><br>'Social isolation is about the number of connections people have or don't have in their lives and while this is not the same as loneliness it is recognised that practical measures to reduce isolation can also help to tackle loneliness' (p5).	Four key themes were identified, all related to aspects affecting participants' perceived loneliness and social isolation<br>- Settings<br>- Activities<br>- Therapy/treatment<br>People	Context/settings and people impact loneliness as well as activities engaged in and support.
[122] Lukes-Dyer (2018, USA - GL)	To explore older adults' perceptions of risks for social isolation and their perceived barriers to social integration.	Doctoral dissertation. In-person interviews were conducted with (N=10) individuals aged 60+ who lived alone, who also made daily journal entries for 2 weeks. Further member-checking telephone interviews. Descriptive individual narratives	Mixed methods interpretive phenomenological study. Framed within context of ecological systems theory and social baseline theory.<br><br>Participant diaries and interviews.	Loneliness: 'The distress caused by an individual's perception that his or her social relationships are inadequate to fulfil his or her desires or needs' (Cacioppo et al, 2015) (p16).	These related to social isolation and risk factors for it, rather than to conceptualisation of loneliness.	'experienced as objective and/or subjective social isolation, the outcomes are the same regardless, all cause increased morbidity and mortality' (p124)

		created based on both data sources	IPA and Colaizzi method for analysis.	Perceived or subjective social isolation: 'An individual's determination that his or her access to various types of social support is inadequate to meet all of his or her needs' (Cole, 2013) (p17). Loneliness is a component part of perceived social isolation and NOT an equivalent.<br><br>Social isolation is a term lacking universal definition. It is culturally variable. For the purposes of this research it was defined as inclusive of both objective and subjective elements.		
[123] Macomber (2017, USA - GL).	To compare the experience of loneliness in two settings, nursing home and at home, and the influence demographics and social support have on this relationship.	Doctoral dissertation. Semi-structured interviews (N=14) conducted in the Great Lakes Bay Region of the US with cognitively able older adults recently discharged from hospital to home (N=8) or nursing home (N=6) settings. Average age was 76 years; F, 6; M, 8.	Qualitative interviews.<br><br>Interpretative Phenomenological Analysis.<br><br>Theoretical framework: Health Belief Model.	'Loneliness is the difference between the amount of social support you expect to receive and the amount you perceive you are getting. (Peplau & Perlman, 1982) (p1)	Themes:<br>- Expectations and perceptions relating to social support<br>- Differences between expectations and perceptions<br>Themes beyond loneliness, including about their setting and discharge from hospital	The majority of participants did not directly report feeling lonely, although some said they were missing people or feeling bored or frustrated.<br><br>The presence of positive social support or of negative social support was a modifying risk factor for the experience of loneliness.<br><br>Conclusions do not relate specifically to conceptualisation of loneliness.

[124] Moore & Preston (2015, England - GL).	An evaluation of The Silver Line, a telephone helpline service linking older people to services. Focuses on: understanding who the service is reaching, why, and whether it suits their needs; whether its use affected loneliness; why volunteers and staff were involved and who they were.	Semi structured interviews with key informants (N=6), focus groups with Silver Line head office staff (N=6), and with Helpline advisers (N=7), interviews with users of the service (N=42).<br><br>Interviews with service users covered their impressions of the service and various aspects of it.	Mixed methods evaluation. Large scale surveys combined with interviews and focus groups	Loneliness and social isolation are distinct but related concepts.<br><br>Loneliness is complex and multi-faceted.<br><br>Isolation is objective, 'defined by the absence of contacts with individuals or communities. (Refs Dykstra, 2009 and Van Tilburg, 1998)<br><br>Loneliness is subjective 'a negative feeling arising out of a perceived lack in the quantity or quality of one's existing relationships. (Refs to Peplau and Perlman, 1982, Perlman and Peplau, 1981) (p17)	Interviewees describe the 'buried loneliness' of callers to the helpline – pretending they are calling for something else.<br><br>The report discusses the possible existence of a 'self-reinforcing loneliness loop'.	Loneliness, isolation and poor health are interlinked and mutually reinforcing.<br><br>Loneliness is defined as a feeling of isolation. It is multi-faceted.
[125] The Mental Health Foundation (2018, England - GL).	To understand whether the Standing Together peer support groups impacted on outcomes	Focus groups were conducted with residents in an extra care housing group, including those with mental health difficulties, dementia, learning disability and/or	Mixed methods report.<br><br>Qualitative focus groups.<br><br>Process evaluation.	'Loneliness can be regarded as the psychological equivalent to social isolation, whereby an individual perceives their existing social relationships to be	Themes discussed included: loneliness and social isolation, wellbeing, and meaningful activities.	Residents felt that participating in the groups helped combat loneliness by strengthening a feeling of social connectedness and belonging.

	related to loneliness and social isolation, emotional wellbeing and meaningful activity.	significant loneliness. Numbers were (N=45) at baseline and (N=57) at follow-up. F, 39, M, 19. Age range 50-90+.<br><br>This resulted in 18 transcripts for analysis.<br><br>Process evaluation telephone interviews conducted with staff members (N=7) and the ST programme facilitators (N=3).	Analysis used the Framework Method (Gale 2003), with themes identified both inductively and deductively.	deficient in some way, either in the number or closeness of contact.' (Steptoe et al, 2013).<br><br>It can be separated into social and emotional aspects (de Jong Gierveld et al, 2016).<br><br>Social isolation and loneliness are distinct but related, and it is possible to experience each on its own or together.	Some residents distinguished between being isolated and being lonely.	
[126] Qin (2017, USA - GL)	An exploration of the effects of loneliness on consumers' digital engagement with social media advertising. Looks at what it is like for a consumer to experience loneliness.	Individual in-depth interviews (N=8; M, 5, F, 3) with participants from a large university and a church in south-eastern US. Age range 19-72. Varied demographic and consumer profile.	Mixed.<br><br>Four experimental studies.<br><br>Qualitative in-depth interviews.<br><br>Phenomenological inquiry.	Loneliness is defined as perceived social isolation. It is 'an aversive affect based on one's self-perception of a lack of social connectedness with others' (Cacioppo and Patrick 2008). A desire for social connectivity is a fundamental and universal human need, connected to our evolutionary history.<br><br>'Aloneness or solitude describes a physiological state characterized by absence of having people around'. It can result from choice, and	The central theme of this research is consumers' perceived social connectedness (or lack of it) in the digital age.<br><br>Three critical insights:<br>- Loneliness is a feeling of isolation, invisibility, reclusiveness and withdrawal.<br>- Lonely people are passive, cautious, indirect, and avoidant.	Social loneliness is triggered by life events such as moves and changes. Emotional loneliness by loss of relationships with others.

				solitude has a positive connotation. It is different to loneliness, which is a psychological state.<br><br>Previous marketing literature treated loneliness as one-dimensional, this author looks at it as a multi-faced social and emotional phenomenon (Weiss, 1973).		
[127] Quinn & Blandon (2014, England - GL)	Evaluation of an intervention aiming to strengthen social ties and improve emotional wellbeing through participation in a weekly singing group. Also aims to promote intergeneration al communication through performances with groups of young people.	The intervention involved older people living in residential care at risk of isolation. Mostly F, all white British, broad range of musical experience. Includes some participants with dementia and physical impairments.<br><br>Observations conducted of 10 singing sessions. N=19 interviews with residents, staff and music leaders.	Qualitative evaluation. Participant observation, reflective diaries and in-depth interviews. Music elicitation tool.<br><br>Thematic analysis focusing on emotional wellbeing, social isolation and loneliness, intergenerational performance, favourite songs and other key themes and issues.	Cattan et al. (2005) defined loneliness or emotional isolation as a feeling of lack or loss of companionship; while social isolation was the objective absence of contacts and interactions with a social network.	Danger of loneliness and social isolation among residents in care facility.<br><br>Need and desire for contact and social networks.	The project is reported as preventing social isolation and loneliness by creating a social network and bonds between people.<br><br>There is a concern that the most lonely and isolated older people are the ones who are not engaging with any activity.<br><br>Having participant facilitators or champions, who are encouraged to take on the role of encouraging and supporting people to take part, may be one approach to this problem.

[128] Sital-Singh et al (2018, England - GL)	A report focusing on the role of local youth organisations in addressing youth loneliness, from the perspective of youth workers.	Interviews were conducted with (N=12) youth workers, who talked about their work with a youth population aged 9-25. A range of geographical locations was included, one in North-West England, one in Midlands, one in South West.<br><br>Three focus groups (N=33 participants). Some participants took part in both focus groups and interviews.	Mixed methods. Focus groups and in-depth interviews.	Loneliness is a common experience for young people. It is challenging to identify and respond to.	Youth workers think that:<br>- Loneliness is a problem amongst young people<br>- It is made worse by cuts to services<br>- They are aware of the subject's complexity<br>- Identify four key risk factors, including difficult situations, weak social networks, high expectations of social networks, lacking skills to cope with difficulty<br>- It's challenging to identify loneliness in individuals<br>Young people don't generally seek help because they may not identify themselves as lonely or admit to it	Presents a visual model for showing the process of loneliness. Three points:<br>- Some people do and some do not experience loneliness, some can 'break the chain'<br>- It can become a downward spiral<br>- Ways to address it at stages (before risk factors occur, after a disconnection, when the emotions attached to disconnection are experienced, when significant impacts of on-going loneliness occur)<br><br>Youth workers have a wealth of insight from their experience of working with young people.
[129] Todd (2017, England - GL)	To explore how museum-based social prescribing programmes reduce social isolation and increase wellbeing of socially isolated older people.	Doctoral dissertation. Interviews, observation and participant diaries with people who took part in a 10 session Museum on Prescription programme. Participant data drawn from pool of participants aged 64-84 self-identifying as lonely or socially isolated and taking part in programmes being run in 6 museums across London and Kent. N=12 took part in qualitative data collection.	Qualitative. Grounded theory. Critical realist framework. Interviews, participant diaries.	Loneliness has been described as a threat to human survival, as a social species that relies on relationships with others to survive (Cacioppo & Patrick, 2008). This has been suggested as a possible explanation for why chronic loneliness is so damaging to health and wellbeing (Davidson & Rossall, 2015).	Key themes:<br>*(1) Social engagement*<br>- A process of building relationships and meaningful connections which increased engagement in the programme<br>*(2) Evaluating self and others*<br>(3) *Getting to know people*<br>- communication helped people to navigate their role in the group<br>*(4) Sharing experiences*	Research adds to the current evidence for a link between wellbeing, social isolation and physical health by identifying how schemes such as this one is beneficial.<br><br>The process of building new relationships and connections that might endure beyond the intervention, is a complex process, influenced by individual characteristics and by previous experiences

130 Zubairi (2018, Scotland – GL).	Investigation of loneliness and social isolation experienced by under-represented demographics in Scotland. To highlight perspectives and voices not often heard.<br>To understand what loneliness and socialisation is, its causes, why it overly affects some groups, and what can be done to tackle it.	Interviews and focus groups with service users and service providers (N=57). Focus in particular on under-represented demographics in Scotland. Women from BAME backgrounds, people living in socio-economically deprived area, in rural communities, paid and unpaid carers of people receiving palliative care.	Mixed methods. Interviews, focus groups.<br><br>No details on analysis.	Author differentiates between loneliness and social isolation:<br>- 'Loneliness is a subjective feeling associated with someone's perception that their relationships with others are deficient'<br>- 'social isolation is a more objective measure of the absence of relationships, ties or contacts with others' – it can be chosen.<br><br>Refs Mijuskovic (2012) on loneliness as universal.<br><br>Refs Olivia Laing on link between loneliness and social exclusion: 'people whose loneliness arises from a state of loss or exile or prejudice' (p6).	'loneliness is like a tree: it has many branches and leaves' (participant quote) (p3)<br><br>A variety of themes are identified including: differentiation between loneliness and social isolation and the way the concepts are often linked, sometimes causally, by participants; bi-directional relationships between loneliness, social isolation and mental health; an understanding of loneliness and social isolation as a form of social exclusion, and; how power or lack of it relates to loneliness. The authors also describe features of the experience of loneliness that are linked by participants to specific life stages, triggers, environmental factors, and socio-economic variables.	Loneliness and social isolation are a Public Health issue. It affects all population groups impacting on their quality of life, resulting in a range of poor and often life limiting physical health conditions and driving down people's mental health and wellbeing.<br><br>Loneliness and social isolation is often triggered, exacerbated and maintained by the social and economic circumstances in which people live including the level of resources such as financial power, knowledge and social capacity that are available to them. Those who are already at risk of being marginalised have a greater likelihood of experiencing chronic loneliness and social isolation and the associated mental and physical health outcomes.<br><br>Further discussion includes that surrounding the place-based approaches participants felt 'nurtured connectedness' and a sense of belonging.
131 Red Cross (2016, England- GL)	This research aimed to provide a rigorous evidence base -	Research was conducted iteratively across four phases, with each phase flexibly adapting to	Mixed Methods. Analytical approach for the qualitative research was iterative and inductive – building	As with other complicated and entrenched social problems, loneliness impacted on people	Loneliness physically impacts on the person experiencing it (the biological impacts); making daily routines and engaging	This research has demonstrated the seriousness of loneliness, not only to individuals experiencing the issue but also its potential impact on

	providing up-to-date evidence about how the UK public experience loneliness, and public and stakeholder priorities for action.	emerging insight from the phase before.<br><br>Phase 1: Mapping the landscape - 27 telephone interviews with expert witnesses.<br>Phase 2: Contexts and needs - Primary research with six target groups. In-depth interviews with 115 people experiencing loneliness.<br>Phase 3: Brainstorming solutions - Online workshops and in-depths, reaching 21 expert witnesses.<br>Phase 4: Testing and validating - Online forum research, reaching 24 people experiencing loneliness to test and validate support solutions.	upwards from the views of participants – incorporating elements of 'grounded theory' analysis i.e. the thematic review and continual analysis of hypotheses from participants' transcriptions and dialogue.	experiencing it in a number of ways that spanned across people's biological, psychological and social spheres. These impacts made connecting with others even more difficult.	socially with people more difficult.<br><br>The psychological impacts are critical, including lower confidence and negative emotions and beliefs. Participants described feeling 'alone', 'trapped', 'without purpose', 'angry' and 'frustrated'.<br><br>People also acted differently when they were lonely (the social impacts). The social impacts included participants shutting themselves off from others, engaging or talking less, taking less care of their appearance/hygiene, and changing their sleeping and eating habits.	communities, our economy, and wider society. We are now at a critical juncture where supporting those who are at risk of experiencing loneliness to become reconnected with society can help those individuals as well as strengthen their communities<br><br>Key quote:<br><br>*"What does it feel like to be lonely? I can tell you exactly, it's like being in a bubble and you want to get out but you just can't, you try and you can't do it, you just can't get out."* (Male research participant, Wales)
[132] Bates & Machin (2015, England)	Understand women's health and well-being perceptions and locality influences on it.	Study undertaken in 2011 with group of women living in a locality in North East England.<br><br>N=15 women participated in 2 focus groups and 6 individual interviews. Aged between 20 - 70 years; most were mothers and most currently in a relationship	Interpretive qualitative design. Individual semi-structured interviews and focus groups.<br><br>Data analysis informed by discourse analysis in the form of critical language study (Fairclough 2001) which conceptualises a person's language as a form of social practice,	The loneliness women experience can impact on lifestyle factors affecting their health and wellbeing.	Four key themes: health and well-being perceptions; mental resilience; low income and choice; and influence of place. The influence of women's geographical location in relation to amenities and services and loneliness were recurring factors in discussion, each influencing lifestyle.<br><br>Causes of loneliness included family situations: being at home	Changed family circumstances, including children leaving home and bereavement, were often root causes of loneliness. Adaption to change was important in re-engaging with the social world. Getting out of the house was an important milestone in adapting and regaining control of life.<br><br>Taking a life course perspective, an asset-based approach has the

			discourse.		with young children; adult children leaving home and bereavement; family thus perceived less as an asset and more as a health inhibitor. Loneliness impacted on mental health, which ranged from low mood to illness requiring acute care; women addressed mental health issues by finding sense of purpose in activities e.g. hobbies, employment, volunteering and socialising.<br><br>Participants identified that purposeful activities mitigated loneliness, consistent with Bryant et al. (2001) suggestion that health was dependent on having something meaningful to do.	potential to capitalise on the assets and protective factors available to women at different ages in their lives. For example, the health benefits of avoiding loneliness through social participation are important to acknowledge, as is the influence of locality on health, well-being and women's decision-making.
[133] Bennett & Victor (2012, England)	To understand what loneliness means to older widowed people.	A qualitative study exploring the experiences of older widowed men and women with an emphasis on understanding emotional and participatory changes following spousal bereavement.<br><br>N=53 interviews of people coded as being 'lonely', aged between 55 and 98 years, who had been widowed between 3 months and 60 years. Male	Secondary analysis. Interviews, where loneliness was spoken of spontaneously were analysed with focus on explicit discussion of being lonely, lonesome or of loneliness.<br><br>Key themes from this reanalysis were: notions of absence, the spatial/temporal aspects of loneliness and unelaborated loneliness. The first two of these	Gives a model of widowhood and loneliness which should recognise that widowed people:<br>• may experience reductions in actual social contacts<br>• may experience incongruence between actual and desired quality and quantity of social contact<br>• may experience losses in the relationships of community	Absence: a central theme within participants' narratives. In 26 interviews, participants discussed loneliness focusing on absence of the spouse in particular, or the absence of people in general (50% of those participants who reported being lonely).<br><br>Time and Place: links with ideas about absence as an explanation and cause of loneliness, relates to the socio-temporal dimension of loneliness. Present in 18 interviews (34% of those	Evidence supports both the cognitive deficit and loss conceptualisations of loneliness illustrating the complex and dynamic nature of loneliness. Widowed lonely people make implicit comparisons between the social contact they would like, often with their spouse, and what they have, often in the context of the presence of general social contacts. This is true also of the temporal context of their loneliness, e.g. wishing for company at particular times. This also supports the deficit

		mean age – 79.76. Women mean age – 71.27.	were further analysed to understand in detail the experience of loneliness.	• almost always experience the loss of a single and significant attachment figure.	reporting to be lonely) and relates to the seasonal and weekly patterns of life rather than time since bereavement.<br><br>Unelaborated Loneliness: 8 described themselves as being lonely without further elaboration. They may have felt the experience and its underpinning meanings were self-evident.	approaches but is more specific than the theory might imply.<br><br>Suggests that people's experience of loneliness resonates with the concept of ''emotional'' loneliness, resulting from the loss of significant social and emotional attachment.
[134] Costello (1999, England)	To show the external and internal social forces that determine the duration and shape of older adults' experience of grief.	'Descriptive bereavement research study' as part of a wider ethnographic study of institutionalised death/dying in hospital. Concentrated on those who had become bereaved (partner dying in hospital) 12 months prior to the interview.<br>Participants interviewed (in their own homes) ranged in age from 66 to 86, average age 75. N= 16. F, 12; M, 4.	Qualitative interviews<br><br>Thematic content analysis of recorded 30-minute interviews.	Loneliness may be seen as social isolation whether it is imposed or when associated with grief as part of a mourner's coping behaviour. Weiss (1973) argues loneliness is integral to human nature, a view shared by Rokach (1990), who suggests that humans experience the terror of loneliness in death and often much in between.	"In particular, the interviewees disclosed feelings of social isolation and reported feelings of loneliness that, although initiated by loss, were seen as forming part of the social experience of ageing", p. 220.	Conjugal bereavement for some older people is irrevocable. It can have a permanent and often damaging effect on their future lives. Assumptions made about how people cope with conjugal bereavement by working through their grief fail to acknowledge the pervasive nature of loneliness and its effects on the older person.
[135] Davies et al (2016, New Zealand)	To examine older widows' experiences of loneliness.	Purposive sampling to recruit N=40 older widows/widowers, age range 70-97. Subjects widowed for at least two years.	Qualitative narrative analysis with thematic analysis.<br><br>Identification of individual narratives that then informed a collective narrative.	Loneliness defined as both cognitive and affective process: 'a discrepancy between one's desired and achieved levels of social relationships' (Perlman & Peplau 1981, p. 32).	"to our knowledge this is the first study to identify the importance of establishing new routines to develop new connections in widowhood", p. 537.	"The study found that the participants negotiated the experience of loneliness following widowhood from an acute phase of experiencing an absence and the associated loss of routine connection to the establishment of new routines that provided new connections

			Qualitative Interviews undertaken by PhD student.	Not all older people are lonely, loneliness not necessarily a constant state.<br><br>Loneliness is risk factor for both mental health and physical problems (Luanaigh & Lawlor 2008, Hawkley & Cacioppo 2010).<br>It is a multi-dimensional phenomenon, varying in intensity, and across causes and circumstances (Heinrich & Gullone 2006).		and a new sense of identity as an individual rather than a couple", p. 532.
[136] Florczak & Lockie (2018, USA)	To explore the continuing bonds with a deceased spouse and the transformative process after losing a partner.	Individual interviews with bereaved individuals from a bereavement group.<br><br>Descriptions of their experiences after losing their long- term partner.<br><br>Participants n=6<br><br>Aged between 67-78 years.	A qualitative design.<br><br>No formal data collection methodology or analysis used. 'Major themes' identified.	The author talks about 'wrestling with solitude'.<br><br>Bereavement of a long-term partner created a 'suffering loneliness which became a pervasive worry as they did not know how to 'transform' and move on.<br><br>Loneliness seen as frightening and distressing.<br><br>Perhaps a temporary loneliness.	Supportive families did not seem to help with this form of loneliness.<br><br>Missing the everyday companionship of a life partner.<br><br>Awareness of participants that they needed to 'move on' from grief and loneliness.	To move nurses and bereavement groups beyond the stage of grief theory.<br><br>Bereavement as 'wrestling with solitude'.

[137] Graneheim & Lundman (2010, Sweden)	To elucidate experiences of loneliness among the very old who live alone.	N= 30; F, 23; M, 7; age range 85-103.	Interviews subjected to qualitative content analysis and cross-checking among the research team as interpretations developed.	Being alone means spending time alone. Living alone means having a single-person household. These concepts may or may not be related to experiences of loneliness. Similarly, a person who experiences loneliness may be alone, live alone, or live with others (Andersson, 1998).<br><br>Authors refrain from defining loneliness because the focus was to elucidate the lived experiences of loneliness as described by the very old who live alone.	"The descriptions of loneliness were twofold: on the one hand, living with losses and feeling abandoned represented the limitations imposed by loneliness; and on the other, living in confidence and feeling free represented the opportunities of loneliness. The findings indicate that experiences of loneliness among the very old are complex, and concern their relations in the past, the present, and the future", summary p. 433.<br><br>"The experiences of loneliness among the very old were described as living with losses and feeling abandoned, representing the limitations imposed by loneliness, and as living in confidence and feeling free, representing the opportunities available in loneliness", p. 436.	"Experiences of loneliness among the very old can be devastating or enriching, depending upon life circumstances and outlook on life and death. We interpreted these two aspects of loneliness as feelings of homelessness and at-homeness", summary p. 433.
[138] Heravi-Karimooi (2010, Iran)	To explore the lived experiences of loneliness among Iranian elders.	Interviews with N=13 elders aged 65 years or older. Participants:<br>• Score up to 34 on Iranian version of UCLA Loneliness Scale (Lower scores show higher loneliness) (9).<br>• Score 6 or up on Iranian version of Abbreviated Mental Test Score -	Qualitative interviews<br><br>Hermeneutic phenomenology	Loneliness conceptualized in three ways:<br>1. Existentially<br>2. Pathologically<br>Sociologically	Four essential themes identified: "an aversive emotional state", "isolated from intimate relationships", "being deprived from social and external support systems", and "being abused and neglected".	Loneliness presented as a lack of intimate relationships with other significant people resulting in the feelings of sadness, abandonment, impatience and anxiety. Therefore, loneliness can be conceptualized as an aversive emotional state.

		cognitive functioning (29).<br>Residing in urban parts of Tehran city in own homes.				
[139] Karlsson et al (2013, Sweden)	To describe young adults' own perspectives on the experience of having a parent who developed cancer when the young adult was an adolescent.	This qualitative interview study focuses on experiences of parental cancer during adolescence.<br><br>N=6 participants; M, 1; & F, 5. Aged between 20-22 years. 5 of participants had a parent die from cancer. Their age at the onset of parental cancer ranged from 14-16 years.	Narrative interview (40-60 mins).<br><br>Content analysis.	Loneliness described both as physical loneliness and as a feeling of being alone. The young adults felt that no one had really understood what they were going through. Although there were many people present, loneliness was still there.<br><br>Loneliness was also experienced when the parent did not explain the disease and the treatment. The young adults missed their parents even before they died, because the illness had caused so many changes. Loneliness expressed as an uncertainty about the future and what would happen if, or when, the parent died. Their death would mean that an important person was gone forever. The young adults also feared that another loved one would fall ill or die.	The message communicated by the young adults was interpreted into 1 theme: Loneliness despite the presence of others.<br><br>Two domains with three categories each emerged:<br>1. Distance<br>• feeling of loneliness<br>• lacking tools to understand<br>• grief and anger<br>2. Closeness -<br>• comprising belief in future<br>• comfort and relief<br>need for support.	The young adults expressed a lack of support during the parent's illness, and spoke about a distance that turned into a loneliness they had never experienced before. They lacked the tools to understand the situation, and felt grief and anger over what the cancer had caused. They found comfort and relief in the thought that this would not necessarily happen to them again, and gained support by talking to family and friends. An undertone of loneliness pervaded everything they said, even when speaking about positive aspects.<br><br>They would have liked to have had accurate information from health care professionals during their parents' illness.

[140] McInnis (2001, USA)	To explore how older adults define loneliness, what contributes to it, how they cope with it and how it might be prevented.	Phenomenological study of loneliness amongst older people living in the community. Participants recruited from a range of settings/groups. All had experience of loneliness.<br><br>20 participants, M, 3; & F, 17.<br>Age range 71-85; 16 widowed<br>16 lived alone; all resident in community.	Phenomenological research design was used<br>to explore the essential elements of the lived experience of loneliness for the elderly living in the<br>community, and to describe the meanings made of this phenomenon by the person experiencing it. Giorgi's method used for analysis.	Loneliness as loss	1. Loneliness occurred when older adult experienced the perceived absence or fracture of important relationships as a result of either death or separation.<br>2. Loneliness occurred as a response to the pain, darkness, and desolation accompanying the perceived ending of a relationship with their loved ones, and their resistance to invitation of openness to the community in which they live.<br>3. Loneliness is avoided or dealt with by using ways of coping which may or may not be helpful.<br>4. Loneliness is a state of anxiety, fear, and sadness influenced by the actual or fear of dependency, and the decreased level of functioning.<br>5. Loneliness is a state of silent suffering in which the<br>person is reluctant or unable to verbalise his or her loneliness	Majority of the narratives confirmed what has been reported in the literature about loneliness, that it is related to a loss, it is emotionally painful, and it has many dimensions.<br><br>A significant portion of the narratives presented new understanding about loneliness such as that loneliness is often a silent-type of suffering; this has not been well developed in the literature.<br><br>Older persons also perceived loved ones as not caring. They struggled with this feeling because it conflicted with their understanding about the busy lives their children often led. Unlike what is reported in the literature, the fear of being institutionalised was not raised, becoming a burden was the most reported fear and obstacle to expressing loneliness.
[141] Muir & McGrath (2018, England)	To investigate experiences of participants of a walk and talk group for long term mental health service users.	Participants of a Walk and Talk group in an inner-city urban environment, who have serious mental health difficulties. The peer leaders of the group theme walk around local history and geography, rather than	Qualitative study, drawing on ecological ontology.<br><br>Thematic analysis (TA) (Braun and Clarke, 2006) was chosen to analyse the data set, to	Loneliness is causative of many forms of distress; a perceived sense of social connectedness.<br><br>Loneliness as a curtailed experience of meshwork. A person who is socially	Four themes:<br>1. Fading lines: fossilised meshworks of loneliness and loss;<br>2. Therapeutic nodes: atomised sanctuary and respite in everyday space;	This paper demonstrates the potential Ingold's<br>(2011, 2015) work for contributing to understanding of mental<br>distress as holistic, embodied and materially embedded experience.

		psychological concepts such as mindfulness.<br>N=6 participants were recruited. Participants were between 52 - 71 years old, which reflects the age profile of group members; F, 2; M, 4.	help identify meaningful patterns across datasets and rich descriptions of phenomena.	isolated may have a meshwork with less activity, growth or fewer connections than a person who feels socially connected and has a life full of possibility.<br><br>Does not distinguish loneliness from social isolation.	3. Reciprocity and authenticity: strengthening relational meshworks;<br>Remaking everyday spaces: expanding meshworks through collectivity.	The meshwork perspective encourages attention to the whole system of a person's life: their past, their environment, and, how they move, grow and forge paths with others through the substances of the world.<br><br>Contains little that is substantive about loneliness in conclusion.
[142] Nunkoosing (2013, England)	Commentary on another paper about a narrative therapy approach to working with men with learning disabilities who are lonely.	Commentary on a piece of action research (original study).<br><br>(For the original study 11 men and women with learning disability)	Not given	Loneliness in the context of a social model of disability.<br><br>People with learning disabilities are often excluded from the networks of relationships that are important to the maintenance of emotional well-being.<br><br>Mentions: a greater acceptance of solitude (as the less aversive twin of loneliness).	It is not the individual functioning of the men that led to their feeling/emotions of loneliness. Instead, this is a result of disabling and excluding social structures that leave them with limited connections to the network of relationships that gives others the feeling of ''belonging to'' rather than ''being apart from''.	It is possible to debate about how one could be lonely even when one is in the midst of a social network or how some of us prefer solitude.<br><br>The absence of relationships is one of the issues that is at the basis of the reported emotional loneliness.
[143] Pettigrew et al (2008, Australia)	To investigate social and solitary pastimes that might ameliorate experience of loneliness among older	N=19 interviews with people 65+ conducted in participants' homes. Participants describe themselves and a typical day, followed by discussion about loneliness and its management.	Qualitative. Semi-structured interviews audio-recorded and transcribed.<br><br>Transcripts imported into Nvivo. Iterative coding process using 'constant comparative	Weiss (1973) definition of loneliness as 'lack of human intimacy', involving both conceptual dimensions of social and emotional isolation, and being 'unpleasant by definition'. Notes that	Subjects perceived loneliness as inevitable part of ageing process, precipitated by social and emotional isolation as a result of deterioration in physical health, death of others, and increasing 'busyness' of family members.	Both social interaction and solitary activities to be considered when designing interventions to alleviate. Solitary activities seen as useful means of 'self-management'.

	people in order to inform future interventions that could reduce negative consequences of social isolation.	Age range 56-95. Living in Perth, W. Australia.<br>F, 13; M, 6. All recruited through contact with elder care agency and retirement village managers. Subjects described as diverse in economic means, self-perceived health and geographical location in city.	method' (Glaser & Strauss, 1967). Member checking of primary themes.	social isolation on its own not always perceived as negative outcome.<br><br>Authors acknowledge that social isolation is widely experienced but see various resources and behaviours as instrumental in determining whether this results in emotional isolation and therefore loneliness.	Most frequently described aspect of experience of loneliness relates to negative aspects of interactions with others, perceived lack of human contact, and satisfying emotional relationships.<br><br>Behavioural coping mechanisms to address loneliness included eating and drinking rituals, reading and gardening.	
[144] Riches (1996, England)	To examine marital adjustment following the death of a child and its effect on parental self-narratives.	Small-scale case study discussed in the light of ideas transferred from earlier research. Conversational interviews conducted by the researcher, a lecturer in research methods in counselling, with female subject. Subject was volunteer, self-selecting, daughter had died 5 years previously, a health worker and member of national self-help network for bereaved parents.	A single case study, drawing on data from other published research. Uses a 'narrative technique' and reports on in-depth interviews, audio-recorded, transcribed and transcripts entered into a 'qualitative software package'.<br><br>Narrative. Data was compared to author's previous observations and interviews and reviewed for 'emergent themes'. Tentative conceptual analyses from these were reported as sometimes	Giddens' work on identity and reflexive self-narrative is used to conceptualise the impact of death on close family members on intimate loneliness.	A form of 'intimate loneliness' is experienced by bereaved parents comprising a complex set of responses to grief.<br><br>These are emotional/internal psychological, physical and structural/cultural.<br><br>Together they can lead to issues around communication between partners. The way in which these issues arise and the ways in which they are managed play a role in the reconstruction of self-identity by the bereaved parent following her loss.	Improved links between professionals and self-help groups and more effective referral systems could capitalize on the support which these informal networks appear to offer in overcoming the intimate loneliness experienced by bereaved parents.

			checked back with participants.			
[145] Rokach (1989, Canada)	To contribute to study of loneliness using a content analysis of real experiences to examine its various causes. This analysis to help in the design of prevention and intervention strategies, and in identifying tools for early identification of the lonely.	Verbatim accounts of loneliness collected from opportunistically recruited participants over a 5-year period. In a group setting, subjects asked to describe their loneliest experience, including thoughts, feelings and coping strategies related to it. Short meetings and informal discussions also held following completion of exercise. N= 528 Participants. F, 298; M, 228. Average age 33.7, majority aged between 19-45. Education levels varied, but 67% had completed 2 years in university or college. 63.2% single.	Qualitative content analysis of verbatim reports of loneliness gathered from opportunistic sample of individuals over an extended period.<br><br>Narrative reports generated analysed by scanning a randomly selected sample of loneliness accounts to determine categories to be used. The remaining narrative reports were then analysed for the presence or absence of derived set of categories of loneliness antecedents. Interrater reliability was calculated on 23 accounts and averaged 94%.<br><br>The author illustrates some of the analysis with examples and quotes.	'Loss' and 'inadequate social support' share similarities between loneliness and feelings of nonbelonging, disconnection and social alienation, what Weiss (1973) terms the 'loneliness of social isolation'.<br><br>Themes within the category of 'personal shortcomings' are far more subjective and cognitively modulated, often precipitated by negative self-perceptions or real deficits. The author notes the connection with the cognitive model of loneliness (Peplau, Miceli and Morasch, 1982).<br><br>The theme of 'crisis' is aligned with Moustakas' (1961) identification of loneliness as an existential phenomenon.	Model of the antecedents of loneliness, with three conceptual clusters:<br>*1.* Relational deficits – missing relationships or those that don't fulfil person's needs<br>• Social alienation<br>• Inadequate social support system<br>• Troubled relationships<br>*2.* Traumatic events – significant and dramatic changes<br>• Mobility / change<br>• Separation from family and significant others<br>• Loss<br>• Death<br>• Relational break-up<br>• Crisis<br>• Awareness of life's limitations and structure<br>*3.* Characterological and developmental variables – individual factors contributing to susceptibility to loneliness<br>Personal shortcomings, Developmental deficits	Loneliness is a potent fact of individual experience.<br><br>The loss of an important person or relationship and the existence of an inadequate social support system are the most common causes of loneliness.<br><br>Personal shortcomings, crisis and the realisation of limitations in the face of the bigger scheme of things are also common.<br><br>The "explicit inclusion of loneliness as a factor in the analysis of human problems could lead to new insights into the treatment of maladjusted human functioning", p. 383

[146] Sagan (2008, England)	To explore mentally ill adults' experience of attending a basic literacy/ expressive writing course in a community mental health setting.	A three-year study with mentally ill adults (N=12) who participated in a basic literacy/creative writing course at their local mental health drop-in centre. Participants were aged between 24 and 65 years with low literacy levels. Most were from White British backgrounds, but three had Afro-Caribbean heritage and two self-identified as Asian/British.	Ethnography: observations, biographical interviews and a single case study.<br><br>Discourse analysis and thematic analysis of interview data.	Loneliness is linked with loss coping, representing a conflict between seeking integration and fearing it.<br><br>Loneliness is viewed as a construct of postmodern society, and influenced educational policies that overvalue outcomes and undervalue learning. These combine to isolate the unqualified and unskilled.	One overriding theme linked the participants: learning to write was viewed as instrumental in their recovery.<br><br>Loneliness was the overarching theme that emerged. The precursor of loneliness is loss: practical (marriage, jobs, lifestyle); cognitive (memory, ability, co- ordination); emotional (loss of the ability to love, the feeling of joy, delight and hope). In the short term, their participation accentuated loneliness and powerlessness, causing struggles with self-image. Overall, the process was therapeutic.	Loneliness may be an integral part of being able to learn and build a new identity – and engagement with biographic material may be healing in this regard.<br>Building a new identity is an elaborate and lengthy process and increases emotional aspects of being lonely particularly fraught in learners with mental health difficulties and painful histories; the paper also, therefore, outlines the shortcomings of current adult and community educational policy.
[147] Theeke et al (2015, USA)	To explore the experience of living with loneliness and multiple chronic conditions for rural older women in Appalachia.	Convenience sample of (N=14) participants, all female (only women responded to advertisements). Recruited from northern West Virginia, in the Appalachian region. Screened for loneliness and diagnosis of at least one chronic condition. Mage=74.4 years. Mean loneliness score = 47.18 (indicating moderate to high loneliness). 5 were married, 5 separated or divorced, 3 widowed, 1 never married. All had	Interviews. Guided by story theory (Liehr & Smith, 2008) and phenomenology (Van Manen, 1997). The aim was to understand 'the very essence of loneliness as experienced by the participants' (p63). Saturation after 14 interviews.	Loneliness is a unique construct, different from (but predictive of) depression (Cacioppo et al. 2006). This is an indicator that loneliness may have its own unique emotional and psychosocial characteristics.<br><br>Authors identify a knowledge gap in understanding the emotional experiences of lonely people and the structure of meaning in the experience as it	Four categories identified, including 13 themes:<br>(1) Negative emotions of loneliness. 5 themes of sadness, disconnection, fear, anger, and worry<br>(2) Positive emotions when not lonely. 2 themes of joy with others, and pride in self.<br>(3) Loss of independence and loneliness. 3 themes of functional decline contributes to loneliness, burden, and gratitude for help.<br>Ways of managing loneliness. 3 themes of remembering	Contributes new knowledge about the experience of anger, fear, and worry when lonely. Negative emotional concepts identified in the study described as 'integral' to the loneliness experience. Discerning the relationship between fear and loneliness could contribute new clinically relevant knowledge. Anger has not previously usually been associated with loneliness, and worry has also rarely been examined in this context.<br><br>Participants described a cyclical (rather than unidirectional)

		completed at least high school education. Household income 'relatively' low.		relates to chronic conditions.	holidays and happier moments, staying busy, and getting out.	relationship between loneliness and functional decline – with functional decline contributing to negative emotions and loneliness, as well as directly to loneliness.
[148] Wijesiri et al. (2019, Sri Lanka)	To explore loneliness in older people (aged 65+) living in care homes in Colombo, Sri Lanka	The participants (*n* = 75) were randomly selected from three different types of care homes: governmental, non-governmental and private, and loneliness was screened using the Revised University of California–Los Angeles (R-UCLA) Loneliness Scale. The 15 participants with the highest score on the loneliness scale were purposively selected for individual interviews.<br><br>Five participants from each care home (*n* = 15) with the highest scores (>60) on the loneliness scale was purposively selected for an open interview to provide an understanding of the loneliness experience.	Qualitative interviews (open)<br><br>Inductive Content Analysis<br><br>All interviews in Sinhala. The interviews took place at the care home in a calm, quiet and convenient area and lasted approximately 45 min (range 30–50 min). All interviews were audiotaped with the consent of the participants and transcribed verbatim. The transcripts included notations of nonverbal expressions, such as silence and laughter, and were translated from Sinhala to English by the first author (MW), who is fluent in both languages.	Explores emotional aspects of loneliness.	Loneliness was experienced as coping with inner pain, which older people described as emotional suffering caused by feeling abandoned from the family network and fettered to the care home, anxious about their future and depressed.<br><br>Participants managed their loneliness by accepting their loneliness, practising religion, staying in touch with family, engaging in daily activities and participating in social activities.	Loneliness among older people was found to impose emotional suffering due to feelings of abandonment by the family and confinement to the care home; such feelings were caused by both physical and socio-cultural limitations in daily life. Therefore, reliable and socio-culturally validated tools for the detection of loneliness would be useful for nurses to become aware of potential stressors and emotional suffering. Similarly, loneliness-alleviating interventions need to be culturally applicable, as staying in touch with family, participating in social activities and practising religion seem to be highly important in the management of loneliness in this family and religion-oriented culture. An insufficient ability to prevent and alleviate

						loneliness among older people residing in care homes could result from a lack of staff education. Thus, nurses should be provided specialised education to support professional care for older people residing in care homes
[149] Wiseman (2008, Israel)	To examine intergeneration al consequences of extensive trauma experienced by parents for the loneliness experienced by their children.	Intergenerational consequences of extensive trauma experienced by parents for the loneliness experienced by their children were explored with adults who grew up in Holocaust survivor families.<br><br>52 adults (M, 26; F, 26). Age 31-46 years (*M* _ 39.35, *SD* _ 3.43); mean number of years of education was 15.12 (*SD* _ 2.49); 94.2% married with children; mean number of children 2.75 (*SD* _ .87).	Qualitative.<br><br>Relationships Anecdotes Paradigm Interviews Narrative recollection<br><br>Phenomenological-structural approach.<br><br>Narrative analysis.	Loneliness is an affective and cognitive reaction to a threat to social bonds and hence a universal experience inherent in the human condition (Rotenberg, 1999).	4 major categories of loneliness experiences in the context of growing up in Holocaust survivor families:<br>(a) echoes of parental intrusive traumatic memories<br>(b) echoes of parental numbing and detachment<br>(c) perceived parents' caregiving style<br>(d) social comparison with other families, in particular the lack of grandparents.	Vulnerability of children of Holocaust survivors to failures in intersubjectivity in their past and current relationships. Corrective emotional experiences of shared meaning and understanding are particularly important for those who grew up with a sense of loneliness in the context of the echoes of parental trauma.<br><br>A more phenomenological approach, based on narrative analysis of descriptive personal accounts of loneliness experiences, is needed to understand the subjective meanings of loneliness for different people in various contexts (Stokes, 1987; Wiseman, 1995).

[150] Barg et al (2006, USA, MM)	To understand how older adults and their primary care providers overlap and diverge in their ideas about depression. To trace the concept of loneliness and its relationship to depression, in older adults. To explore ethnic differences between African American and White participants.	Participants from a large US mid-Atlantic city screened for depression in primary care.<br>Two parts:<br>*Spectrum I (quant) Spectrum II (qual):* series of sub-samples of first part interviewed for thoughts on depression.<br><br>Interviews (N=102, 46% African American) included generation of 'freelists' (N=60), followed by responses to vignettes about depression / open-ended questions. All conducted in own home by professional researchers. Recorded, transcribed verbatim.	Mixed methods. Iterative.<br><br>Participant generated 'freelists'; semi-structured interviews.<br><br>Grounded theory analysis of interview transcripts using constant comparative analysis.<br><br>Cultural consensus analysis of freelist data (saliency scores).<br><br>Meta-inference methods used to interpret findings across qual and quant strands.	Older adults may be inclined to describe depressive symptoms in terms of loneliness.<br><br>Social support factors, loss, personality factors, and differences between the experience of being alone and 'feeling lonely'.	Participants spontaneously linked loneliness to depression. Spoke about this in three ways: (1) loneliness as natural part of ageing (2) lonely people withdraw and are responsible for their loneliness (3) loneliness is a gateway to depression.<br><br>Findings suggest that loneliness may be seen by participants as an 'idiom of distress' (O'Nell, 1996) – and a signal for depression - ref Durkheim 'anomie'.	Attention drawn to older adults' linkage of feeling of loneliness to depression. Reinforces the importance of loneliness as a construct central to experience of late-life depression.<br><br>Participants saw loneliness as leading to depression, as a normal outcome of ageing, and something that it was the responsibility of the individual to avoid.<br><br>Older adults perceived loneliness as both cause and effect of depression; connected it to mental illness; acceptance of loneliness as 'to be expected' seen as minimising suffering that might result from depression.
[151] Drageset et al (2015, Norway; MM)	To investigate loneliness and social support among cognitively intact nursing home residents with cancer, with the aim of identifying ways to improve their care.	Part of a larger study. 60 nursing home residents (all with cancer diagnosis, age 65+, cognitively intact, resident for more than 6 months). At follow-up in 2011, semi-structured interviews conducted with 9 surviving respondents. They were asked what they considered loneliness to be, what factors contributed to it, and its management and	Mixed methods. Qual: semi-structured interviews with sub-sample of participants.<br><br>Qualitative content analysis.	'Loneliness is a subjective and painful feeling common among older people' (p1529). It may result from a lack of satisfying human relationships (Andersson 1998) or from lack of belongingness (Nicholson, 2009).<br><br>Reference to Weiss (1973) conceptualisation of loneliness as both	Six sub-themes identified, and 2 higher level themes:<br>*Experiencing loneliness*<br>- Feelings of inner pain<br>- Feelings of loss<br>- Feeling small<br>*Decreasing loneliness*<br>- Importance of being engaged<br>- Significance of contact with others<br>Importance of occupying oneself	Loneliness is a prominent experience for nursing home residents with cancer. Social relationships are important for coping.<br><br>Loneliness is significantly associated by participants with disease and physical health.<br><br>A sense of competence and self-esteem influences loneliness, as suggested by Weiss (1973).

		to relate this to their own life experiences.		emotional and social, with these elements either co-existing or occurring independently. Emotional isolation can result from absence of close relationship, lack of social integration can cause 'social loneliness'.	'the experience of loneliness of residents with cancer could be seen as a part of feeling small, feeling of loss, and inner pain' (p1534).<br><br>Residents' experiences accord with Hauge and Kirkevold's description of loneliness as unpleasant (2010). Relate to feelings of embarrassment, shame or withdrawal.	Effective coping strategies include social interaction and distracting activity.
[152] Marcille et al (2012, USA; MM)	The purposes were to identify loneliness in rural women with chronic conditions and to identify the major themes represented in their descriptions of their feelings of loneliness.	Secondary analysis of data collected during a 22-week computer-based support and health online intervention in which rural women participated in virtual forums that allowed them to share life experiences.<br><br>The virtual forum, Koffee Klatch, allowed the women to interact directly with one another through initiating or responding to postings. The postings or exchanges included the sharing of life experiences, voicing concerns, and offering advice and support—much as they might have done had they been sitting together in a face-to face group. The researchers	Mixed methods.<br><br>Secondary analysis of a RCT intervention study.	Loneliness can affect the social domain of one's life (Boiven, Hymel, & Bukowski, 1995; Heinrich & Gullone, 2006) and be a negative influence on one's quality of life (Mullins & Dugan, 1990).<br><br>Loneliness is a factor to be considered in planning nursing care for individuals who may be at risk because of physical and/or emotional isolation.<br><br>Notes that a universal definition is not available.	Loneliness themes identified were longing for loved ones, changing relationships, listening in the background, and impact of rural factors.<br><br><u>Longing for loved ones</u>. There were eight postings related to yearning for family members.<br><br><u>Changing relationships</u>**.** Another theme was identified that revealed trying times among the women and their spouses as well as siblings. There seemed to be detachment in some of the marital relationships or lack of support.<br><br><u>Listening.</u> Rather than post online, some women preferred to "stand back in the shadows" and listen. One such participant described herself as a "reader"	The presentation of loneliness will differ from person to person.<br>Providing holistic care for women with a chronic illness includes addressing the possibility of loneliness, which usually hovers beneath the surface; however, it is necessary to assist women in adapting to such changes in their lives that the illness has imposed. An important message to be captured from this research was that the women in this study were very lonely by self-report on a questionnaire, but in their day-to-day lives it was not directly discussed or addressed with the other women in the study.

		monitored the exchanges but did not actively participate.<br><br>57 women were used for the secondary analysis. 96% Caucasian, 4% American Indian. Mean age: 52.2 years			who did not post much but loved listening and learning from others.	
[153] Merz & Gierveld (2016, Netherlands; MM)	To examine whether childhood experiences of parental relationships, current support from siblings and the evaluation of family ties are associated with reduced loneliness in older ever-widowed men and women.	Using Dutch survey data and 18 in-depth interviews from a sub-sample, this study examines the role of family relationships through the lifespan in reducing loneliness among ever-widowed older adults (i.e. persons who have at some time during their life experienced the death of a spouse). Particular attention was paid to childhood memories, family ties and support from siblings.<br><br>18 of 52 older adults in the sub-sample were eligible for this study. All interviews took place in respondents' homes. F, 10; & M, 8. All aged 50+	Mixed method.<br><br>The analysis procedure started with open coding of the interview texts (LaRossa, 2005. Next the coding categories were examined and compared for similarities and differences and brought together in several schemes of related categories.	Perlman and Peplau, (1981) defined loneliness as 'the unpleasant experience that occurs when a person' s network of social relations is deficient in some important way, either quantitatively or qualitatively'.<br><br>Central to this definition is that loneliness is a subjective and negative experience, and is the outcome of a cognitive evaluation of the match between the quantity and quality of existing relationships and relationship standards.	<u>The benefits of warm relationships with siblings.</u> Exchange of emotional support, being one's best confidante, were central categories of quotations of older adults involved in warm and positively evaluated sibling bonds. Moreover, most of the widows and widowers cited in this context were characterised by low levels of loneliness.<br><u>The enduring discomfort of weakened sibling relationships and the wish for restoration</u><br>Data showed the effects of missing the exchange of emotional support with brothers and sisters. Several of the interviewees were stricken by grief, as became clear from the comments elicited, but also from their behaviour: some of the respondents started crying while describing their broken-off relationships with siblings.	Both the quantitative and qualitative results showed that family bonds, in particular sibling relationships and emotional support, may be major resources in reducing loneliness. In addition, childhood memories of relationships with a father have been found to predict loneliness.<br>Attachment concepts may help link personal childhood experiences to social circumstances (Merz, Schuengel and Schulze, 2008) as attachment ties can be considered the first social ties through which children develop and experience future relationships. This process may be key to elucidating the complex interplay among family relations throughout the whole life-course, from childhood well into old age.

[154] Corcoran & Marshall (2018, UK – Chapter [CH])	To assess the literature to discuss how evidence-informed urban design can reduce the experience of loneliness.	Conceptual overview with short extracts of qualitative data from the author's Prosocial Place research programme and other sources.	Conceptual Overview	Defined by Peplau and Perlman (1982) as the discrepancy between desired and achieved levels of social relations, feeling disconnected from other people is a fundamental aspect of loneliness.<br><br>This is as much about the experienced quality of social contact as about the amount of social contact with these factors appearing to matter more-or-less at different stages of adulthood (Victor and Yang; 2012). It extends across subjective time from retrospection to prospection meaning that chronic loneliness is bound up with languishing (the state of low wellbeing) and depression (Cacioppo et al. 2006).	On the face of it, rural lifestyles appear lonelier, suggesting that the dominance of city living may be good for loneliness. For example, Savikko et al.'s (2005) large scale postal survey of older Finnish people showed that the experience of loneliness was associated more with living in rural compared to urban areas. However, unwanted isolation seems to be strongly associated with urban living. The data collected within the UK's North West Mental Wellbeing survey of 2009 (repeated in 2012; Deacon et al., 2009) is testament to this. Compared to the other areas of the North-West coast of England, people living in the city of Liverpool reported lower sense of belonging; participating in fewer organisations and activities;	Emotional loneliness encompasses feelings of desolation and insecurity that result from missing or losing an intimate attachment and so having no-one to turn to. In seeming contrast, social loneliness is characterised by the perceived lack of a circle of friends and acquaintances who can provide a sense of belonging, companionship and community. When thinking about the role that urban design or place-making has in addressing loneliness the obvious conclusion would be that the focus should be on social loneliness. While this seems un-contestable, some qualitative data we have gathered suggests that it may be premature to draw such a narrow conclusion. This data demonstrates that an ambivalent attachment to place, a reaction most closely aligned to emotional loneliness, can itself determine one's sense of belonging – a characteristic of social loneliness.
[155] de Jong Gierveld & van Groenou (2016, USA – [CH])	To review the literature and provide insight around the concept of older couple	This chapter assesses the concept and measurement of loneliness, a theoretical model to investigate the interplay of the main determinants of loneliness,	Conceptual Overview	The couple relationship is a major factor in alleviating loneliness. Midlife and older adults without a couple relationship, especially	An optimal functioning bond with the spouse (in first or subsequent marriage) decreases both the risks of emotional and social loneliness.	Because of extended life expectancy, many people remain in their first marriage until late in life. For others, the opportunities to engage in new partner relationships after

	relationships and loneliness.	several types of couple relationships, and the association between couple relationships and loneliness; the chapter rounds off with suggestions for interventions to alleviate loneliness.		after widowhood or divorce, are at serious risk of loneliness.<br><br>It is the discrepancy subjectively experienced between the desired personal relationships and actually realized relationships—feelings of missing certain personal relationships—that is of crucial importance for the onset and continuation of loneliness. The absence of loneliness includes feelings of social embedment.	Remarriage is significantly associated with higher levels of emotional loneliness, compared with men and women in first marriage (De Jong Gierveld et al., 2009).<br><br>Married persons are better protected against loneliness; those without a spouse and living alone are significantly lonelier. However, the dichotomy of married versus not married masks differences within the respective groups; marital history and gender have to be taken into account to nuance loneliness experiences among those living alone.	widowhood or divorce increase significantly. This is a positive development, as being single is the most important determinant of loneliness. With the increased variety of couple relationships, a more nuanced view of the benefits of these relationships is needed. Social embedding of the couple and the quality of the relationship are more important than just being in the relationship. Partnered older adults whose spousal relationship quality is hampered have an increased risk of emotional and social loneliness.
[156] Haines (2018, England - GL).	To evaluate a project which aims to reduce loneliness and social isolation in older people in Harlow, Essex.	Evaluation study. 43 interviews were carried out with staff and participants of clubs taking part in the project. Project staff (N=4), volunteers (N=10), partners (N=6, 2 of whom were also volunteers), participants (N=25). Interviews were semi-structured and encouraged reflection and sharing of experiences and views.	Mixed methods study.<br><br>Document analysis of project documents. Interviews with staff, volunteers, participants and partners. Observation of project groups in action.<br><br>Qualitative content analysis was used to extract concepts and themes from transcripts.	Social isolation and loneliness are often used interchangeably, but although similar, they are distinct concepts.<br><br>References links between loneliness and health (Campaign to End Loneliness data), and research suggesting that social isolation and loneliness are associated with poorer health outcomes.<br><br>Notes that both concepts are capable of change.	Analysis mainly focuses on outcomes of the project.<br><br>Participants described triggers for loneliness, such as death or loss of a partner.	The groups provided emotional and informational support and gave an opportunity for social interaction, and a sense of ‘neighbourhood’ or ‘community’ – which, it is suggested might alleviate feelings of loneliness and social isolation.

[157] Hall (2012, USA - GL)	To investigate the experience of being a married woman who feels alone in her marriage relationship.	Doctoral dissertation. Interviews with N=9 middle-aged women who professed to have lived with 'coupled loneliness'. Subjects invited to share feelings and perceptions as related to the topic. Ages range from 41-54, all were white, middle class, professionally employed, and well educated.	Phenomenological.<br><br>Qualitative interviews. Emphasis on study of life experiences.	Conceptualises idea of 'coupled loneliness'<br><br>Informed by Moustakas (1994)	Five essential themes. Feelings of:<br>- Disappointment (discrepancy between expected and real outcomes)<br>- Abandonment (lack of support, encouragement and interest)<br>- Devalued (emotional wounding)<br>- Powerlessness (inability to bridge lack of communication)<br>Guilt (for focusing on unhappiness in the marriage)	The essential constituents of the experience of living with coupled loneliness are vitally important to the physical, emotional, mental and psychological wellbeing of the women who live it.
[158] Henrich (2019, England - GL)	To examine the lived experience of loneliness in intimate partner relationships (LIPRs) amongst a sample of 6 middle-aged men.	Audio-recorded, in-depth, semi-structured interviews that focused on exploring men's lived and felt experiences of LIPRs.<br><br>(n = 6) participants aged 40-65 years.<br>The participants were recruited via snowball sampling. The interviews were conducted between May and August 2018, at a location agreed between the researcher and participant.	The transcribed interview data was analysed using interpretative phenomenological analysis (IPA). Data analysis was further guided by Smith et al. (2009), who provide a six-step approach to data analysis.	Conceptualisations are:<br>1.<u>distressing feelings</u> of loneliness and isolation from emotional abandonment, feeling attacked, criticized and unable to escape, and reliant on their partners for their self-worth to alleviate the situation.<br><br>2.<u>together but separate</u>, although physically together and sharing tasks, can feel separate and disconnected emotionally and from physical intimacy and unable to share experiences leading to pain and loneliness.	LIPRs were characterised as:<br>1.<u>Distressing feelings</u>: feeling attacked, criticized and emotionally abandoned, not recognised for what they do; existence feels futile & empty; feelings of despair, isolation, dread & a longing for connection and to be seen. Some expressed a passivity that their worth is reliant on partners, possibly due to feeling overwhelmed by loneliness & unable to escape; hopelessness due to an inability to change their situation, thus feeling lonely, but hope in the possibility of meeting someone new.<br>2.<u>Together but separate:</u> a paradox, as participants were both 'with' but also separate &	These findings fit within the literature in that:<br>1. distressing feelings reflected in Hall's (2012) study on LIPR;<br>2. the paradox that participants felt together, but separate fit with Dahberg's (2007) study suggesting LIPRs are confusing, eliciting contradictory feelings (e.g., closeness and isolation);<br>3. making sense of LIPRs seen in Brown's (2018) study that loneliness in close relationships is not always recognised immediately and some time to recognise and name their experiences as loneliness.<br>4.Masculinity as a straight-jacket theme is the only point not in the literature. This links to Butler's (1990) theory of gender performativity in which

				3. <u>gendered ideas of masculinity</u> 'man up' having to be strong, wear the trousers, governing the relationship and leading to an inability to share their experiences with other men and a 'double loneliness'.<br><br>4. taking time to realise they are lonely, anger covering deep wounds and hurt.	disconnected from their partners; a lack of physical intimacy, exacerbating their feelings of raw pain and isolation, or engaging in sex, but feeling disconnected and lonely in the experience; unable to share experiences and feelings with partner.<br><u>3.Masculinity as a straight-jacket</u>: a fear of sharing feelings of LIPRs with other men as it might compromise their masculinity, resulting in a "double loneliness"; men should be strong and not show their vulnerability: "man-up" reflects the idea that real men should be brave and face loneliness; feeling lonely makes them weak and that "she wears the trousers"; a devaluation of masculinity where they felt their partners did not value their role as men, linking into narratives about gendered roles such as authority and manhood, leading to tension in their identity as men and painful experiences.<br><u>4.Making sense of LIPRs</u>: feeling it was their own fault, searching for causes, taking on an insidious quality, as it creeps into one's relationship slowly, often unnoticed; many expressed anger, but which	gender is a series of repeated socially embedded acts positioned in a heterosexual framework that lead to feelings of performing it in the right or wrong way. Performing one's gender in ways that do not align with this framework results the individual becoming culturally illegitimate and can lead to acts of normative violence, such as bullying & social exclusion.<br><br>Within Connell's (2005) concept of hegemonic masculinity, gender is a configuration of social practice, such that the western cultural idea of masculinity is self-sufficiency, emotional resilience, and occupying the role of 'provider', and 'protector', Non-hegemonic aspects of masculinity i.e. gay masculinities can be subordinated to this. Thus, there is a policing function around concepts of masculinity.

					covered deep wounds and hurt; and many turning inwards and reflecting on their experiences.	
[159] Cherry & Smith (1993, USA)	To explore experiences of loneliness amongst AIDS patients in Florida, USA.	This study examines the way the accepted typology of loneliness – emotional isolation, social isolation, and existential loneliness – is revealed in the narratives of AIDS patients.<br><br>N= M,8; all with AIDS, aged 24-46.	Narrative qualitative design. Analysis focuses on meaning of story selection and story content, including plot, characterisation, and cues for interpretation. Demonstrates that the typology can be identified in first-person accounts as well as in quantitative studies. Nine plot lines, three for each loneliness category identified, extend and enrich the loneliness construct and suggest practical ways to improve care of people with AIDS.	Authors do not settle on one working definition but comment on the combination of social isolation and emotional isolation (Weis, 1973) and existential loneliness (Moustakas, 1961). Deficits in a person's social relationships lead to loneliness. Loneliness differs from social isolation in that loneliness is a subjective experience. Deficits in a person's social relationships can result from lack of involvement in a satisfying social network or from the absence or loss of meaningful friendships.	Feelings of alienation and stigmatisation based on perceived isolation from society. Many patients described the world as an 'us versus them' scenario. Separation from society and overzealous stigmatization signalled out men with HIV/AIDS and left them marginalised and lonely.	Narratives in this study show the very real feelings of loneliness experienced by men with AIDS, and point towards better ways to care for AIDS patients.<br><br>Alienation, rejection and feeling marginalised / outsider.
[160] Chung et al. (2020, Hong Kong))	To describe the experience and coping of Existential Loneliness in Hong Kong Chinese and Swedish older	Using Thorne's (2004) interpretive description, two series of one-on-one, in-depth semi-structured interviews were conducted about their experience of EL 6 months apart between March 2017 and February 2018.	Qualitative design.<br><br>The interviews were audio-taped & transcribed verbatim in the original language, and then translated into English.	EL is conceptualised as "overcoming", rather than "being trapped in an anxious state" without meaningful interpersonal relationships, and "imprisoned" in feelings	Two Themes:<br>1: *Feeling EL*- to overcome it, you need to feel EL first.<br>A: *Loss of control of life*: Chinese and Swedish felt this was due to psychological decline, and the ensuing feelings of powerlessness (to make their own choices) and vulnerability,	The study sheds some light on the cross-cultural experience of EL. While the overall experiences of EL are comparable between the Chinese and Swedish participants, strategies that address the disrupted self will need to be tailored to how

	adults living in LTCFs.	Participants were interviewed a second time in order to verify the initial findings based on the first set of interviews and explore any changes in their EL experience over time.<br><br>(n = 13) Chinese and (n = 9) Swedes living in LTCFs in Hong Kong, China and Malmo, Sweden, respectively.	Analysis began with developing the coding framework following Thorne's interpretive description of describing and comparing interviews, noting similarities and differences.<br><br>All transcripts were coded using the QSR NVivo 11 data management software. The research team then conducted thematic analysis to reflect EL, then finalising the themes and subthemes into a coherent set of constructs, where their relationships were examined, and until a coherent interpretive description of participants' experiences was achieved. Data saturation was reached of the analytical categories.	of being useless and unconnected.<br><br>The participants conceptualised EL in terms of:<br>1) the loss of control of life, 2) failure to be understood, 3) experience of isolation, and 4) inability to identify meaning in their lives.<br><br>EL appears similar to the concept of disrupted self—a self-based notion of aging as having a meaningful and productive life, with meaningful roles and life goals, contribution to family and society and being in control of their lives and their world.<br><br>EL includes the loss of their identity and ability to perform meaningful acts.<br><br>Over time, EL was felt to be unavoidable and beyond their control, so they chose to cope with it. Having no alternative ways to manage the	and that family members made decisions for them.<br>B: *Failure to be understood*: Both participants felt down as no one understood their feelings despite their communicating them.<br>C: *Experience of isolation*: both felt this, distance from others and questioning where they belonged, due to physical decline and death of loved one, and feeling like a burden.<br>D: *inability to identify meaning*: both felt present and future looked bleak, feeling unworthy and insignificant.<br>Theme 2: redefining being trapped as a thing that *can be overcome through self-regulation*.<br>A: *accepting and reframing reality*: accepting limitations, weaknesses, that change necessary, and being in the present rather than worrying about the past/future.<br>B: *reflecting the self:* opportunity to examine thoughts and feelings and redefine purpose.<br>C: *questing for meaning:* Chinese participants recognise EL as challenging, accept feelings towards it, regulate negative emotions by reframing their experience, and identifying new opportunities and finding a new reason for living.	people define themselves (e.g., based on social and cultural roles vs. individual pursuits, achievements, and relationships), which in part is shaped by their cultural background.

				disrupted self, they turned inwards by self-regulation. They hoped to lessen the sense of negativity of EL by accepting and reframing their experience, examining oneself, questing for meaning, and redefining success in life.	D: *redefining success in life*: no embedding their success in the past.	
[161] Dahlberg (2007, Sweden)	To "present a structure of meaning regarding the phenomenon of loneliness" (p. 197)	An attempt to answer questions about the essence and definitions of loneliness through capturing the phenomenon's existential meaning without reduction of its complexity. Study also poses question of how loneliness is related to health and wellbeing.<br><br>Interviews all conducted by different students. N=26 interviews analysed, M and F, age range 12-82 years. These were the "high-quality" interviews selected from around 100 interviews conducted. There is an "overweight" of middle-class, and female informants.	Interviews. Phenomenological approach, "Reflective Life World Research", drawing upon Husserl and Merleau-Ponty.<br><br>Analysis and synthesis in a "dynamic approach to data", leading to a structure of meaning as respective meanings (or "figures") supersede or blend into each other.	Conceptualisation is taken from the data:<br><br>Loneliness can be a "hard" phenomenon, without any others; it can mean being lonely with others, sensing isolation even in the midst of a crowd or a group; it can be strange, wrong, ugly, shameful; it can be something very good, chosen or voluntary, restful and creative.<br><br>Citing Sartre too, loneliness is seen as the absence of presence.	Loneliness is a phenomenon "closely related to its context", p. 205.<br><br>"The phenomenon of loneliness stands out in meaning as ''figure'' against a ''background'' of fellowship with ''important'' people. In order to understand loneliness and its meanings, we must first consider this ''background of fellowship'' and its relation to loneliness", p. 197.	"The descriptions in this research touch upon experiences that reveal the search for more immanent and transcendent meanings with the goal of finding oneself in the roar of everyday existence", p. 205.<br><br>Complex definition but absence of presence, transcendent experience.

[162] Goldberg (2001, USA)	To understand loneliness as a consequence of time disturbance (from the point of view of a psycho-therapist)	This starts with an existential reflection by/from *Hamlet* on the nature of time and how time is judged. It is then based upon a single case study to show how "loneliness is dependent upon a disturbed consciousness of time" (p. 269).<br><br>One participant, a woman in her early 30s referred by family physician because of severe depression. Seen as a suicide risk by the physician should her "philandering husband" leave her. Taught structural engineering at a university.	Qualitative, a single case study of a patient in therapy.<br><br>Much of the analysis is the application of philosophical / psychoanalytical concepts and theories to the patient's experiences.	"Loneliness" is used alongside "despair" as a premise for understanding the source of unhappiness.<br><br>Loneliness is a condition in which people find themselves entrenched in lives that are barren of interesting and trusting companionship. The failure to foster caring for others leads directly to feelings of inadequacy, depression, and intense loneliness.<br><br>Loneliness is the denial of the present moment, its possibilities and its demands, which poses an existential dilemma.	The subject is said to have a distorted picture of herself in adulthood, becoming a "warped person' as a consequence of earlier insecurities. Humanistic psychologist John Cohen is quoted on this.<br><br>Much analysis comprises cross-references to author's own writings.	Desperate loneliness can rob individuals of their own agency.<br><br>An image of oneself outside of reality and outside of normal time.
[163] Goosens et al (2015, Belgium)	To explore communication difficulties and the experience of loneliness in patients with cancer dealing with fertility issues.	N= 28; F, 21; and M, 7; patients with cancer with potential fertility problems as a result of treatment.	Semi-structured face to face interviews. Grounded theory approach using the constant comparison method with simultaneous analysis and data collection.	Loneliness identified as a consequence of a medical situation, but not defined or conceptualised.	"Loneliness was a central theme in the experience of potential fertility loss among patients with cancer. Feelings of loneliness resulted from communication difficulties between the patient and members of his or her social environment or healthcare professionals because of several underlying processes and influencing factors", p. 34 summary chart.	"Loneliness was a strong and common feeling among patients with cancer. Patients, members of their social environment, and healthcare professionals experienced difficulties in communicating about fertility in the context of cancer, leading to patients' feelings of loneliness", p. 34 summary chart.<br><br>Feeling like one is outside of the social experience.

[164] Hemberg et al (2018, Finland)	To explore and understand experiences of suffering from loneliness in older adults receiving home care.	Face-to-face interviews; N=17 participants (F, 12; M, 5) aged 72–95 years in different life situations receiving home care.	Qualitative interviews. Hermeneutic framework	Highlights different definitions including (1) Peplau and Perlman (1981) – discrepancy between desired and achieved levels of social relationships; (2) Savikko (2008) – subjective experience of lack of satisfying relationships.<br><br>Conflates loneliness and social isolation – argues two types of loneliness as social isolation (lack of personal relationships, and emotional isolation (lack of intimacy).<br><br>Loneliness differentiated from being alone.	Being 'homeless' in/through life – loneliness expressed as suffering.<br><br>Subcategories of 'existential' suffering through loneliness: (1) loss of communion with one's partner or other loved ones; (2) loss of meaningful social activities due to isolation and; (3) loss of health due to frailty and vulnerability.	When existential suffering arises due to loneliness, this constitutes a profound potential threat to older adults' dignity and, thereby, health. The alleviation of such suffering is essential.
[165] Larsson (2017, Finland)	To explore frail older (>75) persons' Existential Loneliness as interpreted by their significant others.	Significant others of frail older people defined as older persons >75 in need of long-term care related to multiple diagnoses, physical disorders, and functional impairments receiving care from formal caregivers from the municipality or the county council.<br><br>23 frail elders were identified and 19 significant others. The	Qualitative interviews with significant others. Two interviewers used: one to take notes/one to ask questions. Interviews lasted 40-90 minutes (median 51)	Theoretical underpinning is existential loneliness.	Three themes identified: limitations in body and space; process of disconnecting, loss of people places etc; disconnection from the outside world	Significant others perceive that the older persons experience existential loneliness (1) when they are increasingly limited in body and space, (2) when they are in a process of disconnecting, and (3) when they are disconnected from the outside world

		significant others were predominantly female=13. Mean age 63 (range 48-96). 10 were children/in-laws, 4 wives, remainder other relatives. 19 significant others generated 18 interviews (one done as a couple)				
[166] Larsson et al. (2019, Sweden)	To contrast frail older (>75) persons' experiences with their significant others' perceptions of existential loneliness (EL).	Interviews were conducted with frail older people (n = 15) and with his or her significant other(s) (n= 19), to explore their experiences and perceptions of EL.<br><br>Field notes from the interviews were also collected as data.	Qualitative design<br><br>The data collected were arranged in 15 cases, each included the narratives of one older person, his or her significant other(s) and the field notes.<br><br>The 15 cases were analysed by a group of four authors, using Braun and Clarke's (2006) guide on thematic analysis in psychology.<br><br>The analysis included familiarisation with the data, comparing authors' impressions, coding by the first author, group discussion until reaching an agreement on the final themes.	A deeper feeling of loneliness, called EL, a feeling that can come and go.<br><br>For frail older people EL is linked to meaningless waiting, lack and longing for a deeper connectedness and restricted freedom. Waiting makes older people feel that their life has become meaningless, empty and isolated, giving rise to EL.<br><br>EL for older people rises when they feel excluded when they are among people whom they do not feel any connectedness with, or when they have no one to share essentials in life with.<br>The older persons experience EL when they	Significant others have a limited understanding of the older people's EL.<br><br>Meaningless waiting in contrast to lack of activities<br><br>Longing for a deeper connectedness in contrast to not participating in a social environment<br><br>Restricted freedom in contrast to given up on life.	The findings add knowledge on the meaning of EL for frail older people. Also, significantly, by illustrating that there is a gap in the understanding of older people's EL, this study highlights the need for listening more to their needs, in order to be able to provide effective healthcare.

				are not given the opportunity to express their wishes or choose by themselves in their everyday life, since they are dependent on other people's help.		
[167] Nillson (2007, Norway)	To investigate the existential significance of loneliness for the person living alone who suffers from a serious mental illness.	A year-long empirical investigation, for which data includes 400 pages of text from participatory observation of an ethnographical nature and research conversations.<br><br>N=8 patients, both sexes, ages between 20 - 50.<br><br>All: had an earlier shorter or longer admission to a psychiatric institution; are described by the Psychiatric health service as being seriously mentally ill; had diagnoses of schizophrenia; lived in their own apartment and had frequent contact with the psychiatric nurse in the community health service.	Qualitative study. Researcher participation described as an element in a combination of participatory observation, ethnographical approach and conversational research interview.<br><br>Gradual hermeneutic understanding of the phenomenon loneliness attempted through the use of six different theories congruent with the caring science paradigm. By employing the broadest possible approach, attempts were made to ensure that the knowledge gathered was indeed knowledge about the field of loneliness (26). The purpose was to	Participants experience the pain of loneliness as almost always present, like an invisible follower in feelings, such as anxiety, defencelessness and loss, but made visible in an external mask of self-isolation.<br><br>The experience of a lack of feeling at home appears to be related to two conditions: experience of an inner homelessness and the other is that although their place of residence has become an arena for the carrying out of different types of care, the informants still feel lonely.	At the doing level, loneliness is experienced as aloneness and isolation in the sense of being excluded from 'normal life'. The informants miss company and feel they are forsaken by their earlier friends. Loneliness is experienced as aloneness and isolation from 'normal life'.<br><br>Loneliness manifests itself as pain and is almost always present as an invisible companion, in the form of an outer isolation of the self. It is possible to observe the informants' greatest loneliness – a fundamental loneliness – which appears to be connected to God having abandoned them.<br><br>Informants' interests in art and their activities e.g. painting, poetry and music etc., can be understood as the positive form of loneliness (solitude), where the different forms of art can increase the self-image	The informants' loneliness is visible at the 'doing' and at the existential levels.<br><br>Informants experienced loneliness as so tormenting that suicide was considered as a possible release.

			achieve a deeper pre-understanding before starting on an empirical investigation.		and liberate positive forces. This can be seen as a health potential on both the doing level and on the existential level	
[168] Nortvedt (2015, Norway)	To explore how immigrant women on long-term sick leave in Norway due to chronic pain experience their illness and their relationships at work and in the family.	First author conducted participant observations during two rehabilitation courses, each spread over 10 days during the course of 10 weeks each (totalling 45 hours).<br><br>The sample included immigrant women aged 30 - 56 years. Eleven were on partial sick leave and three were on full sick leave. The women came from low or middle-income countries.	Observations and qualitative interviews. Hermeneutic approach to the analysis.	Lonesomeness was related to the experience of being locked inside the home due to lack of a social network or the lack of possibilities to live in another place or country.	One main finding: 'A lonely life'. Two related subthemes: 'Shut inside the home' and 'Rejected at the workplace'.<br><br>Not being understood because of their invisible illness or their cultural and linguistic differences implied a lonesome life.	Immigrant women on long-term sick leave due to chronic pain experience hardships in their daily life, both at home and at the workplace. This became apparent through the descriptions of low self-worth, shame, lonesomeness, rejections from spouses and neighbours and their own confinement because of painful bodies and distressed minds.<br><br>Invisibility of culture and illness defines loneliness
[169] Nystrom (2006, Sweden)	To analyse and describe lived experiences of aphasia and the struggle to regain the ability to communicate (part of a larger study).	Interviews conducted, then repeat interviews when needed to check meanings and conclusions. Published books and unpublished diaries also used. Participants were purposefully selected from two branches of the Swedish National Fellowship for Aphasia, to obtain variation in age, gender, type of aphasia and time since cerebral lesion. Type of aphasia was reported by the participants and/or their spouses.	Qualitative - lifeworld hermeneutic approach	Not conceptualised.	Existential loneliness creates feelings of alienation.<br><br>Previously unknown feeling of loneliness, which increases and extends to all domains of everyday life, can follow the single-handed struggle to regain the ability to communicate.	A person with continuing aphasia must cope with existential loneliness as a consequence of inability to communicate.

[170] Rosedale (2009, USA)	To describe the experience of loneliness for women more than a year following breast cancer treatment.	Interviews with purposive sample of 13 women between 1-18 years after treatment. Recruited through cancer survivor's network. Pre-interview demographic and treatment questionnaire and participants asked to describe experience of loneliness and to share written / artistic expressions on experience. These materials used as prompts in interviews. Interviews recorded and transcribed verbatim. Research field notes and reflexive journal. Interview transcripts analysed, assisted by Atlas.ti. Member checking. All participants agreed description produced.<br><br>Detailed demographic and clinical characteristics recorded, including:<br>- race (African American, 3; Caucasian, 13)<br>- marital status (married, 6; divorced, 6; never married, 1)	Qualitative. Phenomenological (Streubert's phenomenological method based on Husserl).<br><br>Phenomenological analysis process to create formal description of phenomenon of 'survivor loneliness'. Analysis constant and immersive throughout data collection.<br><br>Study focused on loneliness as central concept.	'Survivor loneliness' is conceptualised - comprises a sense of being alone in awareness of mortality, invalidated in the experience of ongoing symptom burden, experience of a changed sense of identity and connection and an altered threshold for distress.<br><br>This phenomenon is not adequately explained by reference to single theoretical framework.	Themes of phenomenon described:<br>(1) Emerging consciousness: loneliness as part of ongoing experience<br>(2) Transcending time: at the beginning people feel sorry for you, but then it means less to them<br>(3) Misunderstanding: others misunderstand impact of experience<br>(4) Inauthentic mirroring: pressure to be a 'hero'<br>(5) Fragile vital connections<br>(6) Withholding truth: not sharing or masking aspects of experience	First study that directly investigates and describes experience of loneliness for women diagnosed with breast cancer and describes a new concept – 'survivor loneliness'.<br><br>Transcending time and consciousness and being misunderstood.

		- education (high school, 2; associate degree, 4; bachelor degree, 4; master's, 3)<br>- socioeconomic status (declined, 1; less than $50K, 1; $50-99K, 8; $100-150K, 2; more than $150K, 1). Also recorded surgical and/or adjuvant treatment received.				
[171] Sagan (2017, Scotland)	To investigate the experience of loneliness among people diagnosed with borderline personality disorder.	Freely associative interviews in person or by Skype on the subject of participants' experience of loneliness. Study underpinned by critical constructivist perspective on narrative. Thematic analysis (Braun and Clarke, 2006).<br><br>Participants were N=7 adults with diagnosis of Borderline Personality Disorder (BPD) willing to talk about experiences of loneliness. Aged 25-61. Ethnicities including White British, British Asian, European. Identified through mental health online networks.	Qualitative, narrative phenomenological approach.<br><br>Data analysed by coding dominant categories that were then reanalysed to identify sub-themes. Thematic analysis (Braun and Clarke, 2006) used to identify a 'meaning imbued understanding of experience'.<br><br>Emphasis was on exploring the narrative of lived experience of loneliness.	Loneliness is defined with reference to its phenomenological experience. More attuned to the possibility of loneliness as potentially restorative and creative' (Moustakas, 1961; Mijuskovic, 2012; Willock et al, 2012) (p214). The study also references the notion that solitude is an ontological necessity (Heidegger).<br><br>In the psychoanalytic context: references to Winnicott - the capacity to be alone as 'both a developmental achievement and an existential necessity' (1958) (p.214). Also, to the social needs	All participants disclosed difficult or traumatic early years experiences which they felt had left them with chronic feelings of emptiness.<br><br>Participants saw loneliness as an inherent part of themselves, a trait not a state.	Their descriptions suggest that they experienced it as a Heidegerrian 'inability to find or to feel at home in the world', something that is further entrenched by stigma (p217). Early, or repeated trauma leads to those with BPD dissociating from others: 'trauma and un-relatedness lie at the core of the loneliness experienced by people in this study' (p217).<br><br>Some of the strategies used by people to manage the experience were risky or harmful.<br><br>The study highlights the 'complexity and specificity of loneliness when interfaced with particular conditions' (p217).

				perspective (John Bowlby) and Hojat who sees loneliness 'as a pathological state […] due to a breakdown of social needs in early childhood'.		
[172] Sand et al (2006, Sweden)	To explore experiences of existential isolation, including emotions and perceptions, in palliative patients with cancer and their families.	Study of existential isolation in cancer patients (N=20, F, 12; M, 8) and family members (n = 20) in an advanced hospital-based home care service. Participants were Swedes who defined themselves as nonreligious. The mean age was 63 and the range was 21 to 91 years of age.	Hermeneutic method. Semi structured interviews. Analysis sought to gain a deeper understanding of expressed meaning and implicit issues, for example, it was assumed that informants might not give explicit expressions due to fear or defence mechanisms.	Existential isolation is a basic experience that is shared by everyone. EL is particularly threatening and also shaming.	Loneliness is difficult to talk about directly. Experiences of being alone in "a world of one's own" were common. EL is founded in:<br>1) bodily changes that cause feelings of separation, unfamiliarity and powerlessness.<br>2) reduced ability to protect themselves from thoughts and feelings relating to the impending death.<br>3) other life changes, reduced social interaction,<br>4) the responses of others: being treated insensitively, being left alone when he or she needed support, being avoided because of fear.<br>5) the feeling that healthy people are incapable of understanding.	The data revealed experiences of existential loneliness with the impending death as a primary source. While existential loneliness is impossible to eliminate and is a basic given for all humans, respect and empathy shown by caregivers can assuage feelings of loneliness.
[173] Sjöberg (2018, Sweden)	To describe how EL was narrated by frail elderly people.	Study of existential loneliness in frail older people (N=22) who were receiving long term care and services.<br>Participants were aged 76 to 101 years (median 85)	Narrative interviews. Conventional inductive stepwise qualitative content analysis.	Existential loneliness (EL) is an unavoidable part of the human condition, due to an awareness of mortality and	Two categories and seven sub categories emerged.<br>(1). Being acknowledged by others (being the focus of others' concern; encountering intimacy; and having	Existential loneliness can be eased by experiencing meaningful togetherness with others and oneself when something else<br>comes to the forefront, pushing EL to the background.

		including M, 12; F, 10. Six were widowers, nine were widows, 18 lived in single households and four were cohabiting. Participants were required to have the ability to participate in an interview in Swedish and were required to have experienced EL.		described as disconnection from life. Losses experienced by older people, increase the risk of loneliness. EL is linked with both social loneliness and emotional loneliness. It is a sense of being fundamentally separated from other people and the universe.	meaningful exchanges of thoughts and feelings).<br>(2) Bracketing negative thoughts and feelings (adjusting and accept the present situation; viewing life in the rear-view mirror; being in contact with spiritual dimensions; and having the opportunity to withdraw and distract themselves.	Frail older peoples' opportunities to ease EL may be facilitated<br>by health care staff (HCS) providing person – centred care and<br>create possibilities for solitary time and meaningful togetherness.
[174] Stein et al (2014, Israel)	To examine the qualities and characteristics of loneliness as part of combat-related trauma in veterans of combat and captivity.	Study of loneliness in veterans who have given testimony at the Israel Trauma Centre for Victims of Terror and War (NATAL) between 2007 and 2012. Participants were 19 combat veterans (aged 31–68 (M=52.7), and seven ex-POWs, ages 56–67 (M=61.75).<br>12 (46%) had exhibited PTSD symptomatology during the screening.<br>The time elapsed since participants' combat exposure or repatriation from captivity was generally over 30 years.	IPA<br>Life story interviews of 3-8 hours duration.<br>Thematic analysis.	Loneliness is regarded as fundamental experience of aversive isolation, distinct from social isolation.<br><br>Current definitions of social, emotional and existential loneliness do not address trauma.	The analysis led to the identification of four recurring themes.<br>1) 'A different world', the sense of extreme isolation and alienation.<br>2) Those Who Have Been There (and Those Who Haven't)" - The Sense of Two Populations.<br>3) "No One Can Understand"—The Sense of Failed Intersubjectivity 4) "There Are No Words"—The Sense of incommunicable experience.	Loneliness is not a lack of intersubjectivity, but a *failed* intersubjectivity found in regard to experiences from the battle or captivity or experiences that took place in the posttraumatic reality.<br>A new term is proposed: "loneliness of experiential isolation," or in short "experiential loneliness."<br><br>Achieving a sense of shared identity is unattainable for this group, whose traumatic experiences lay outside the range of usual human experience and language.
[175] Sundström (2018, Sweden)	To explore health care professionals' experiences of their encounters with older	Part of the larger LONE study exploring existential loneliness among frail older people Focus groups (N=11) held at participants' workplace in mixed groups of 3 to 8, led by two	Focus groups. Thematic analysis.	Existential loneliness is a condition, experience and process related to feeling disconnected from the world, lost without a purpose, and adrift in life.	The overarching theme was barriers. This encompassed:<br>(1) Insecurity when trying to interpret and understand needs and desires;	Professionals perceived existential loneliness to appear in various forms associated with barriers in their encounters, such as the older people's bodily limitations, demands and needs perceived

	people they perceive to experience existential loneliness.	researchers using a topic guide.<br><br>Participants were 61 health professionals working with older people in health and community settings in rural and urban areas of Southern Sweden.		It can arise when people face new situations or in times of uncertainty such as during an illness. Responses can vary from fright, excitement, acceptance and growth.<br><br>Draws on Emmy Van Deurzen's framework with four dimensions: physical, social, personal and spiritual worlds (van Deurzen, 2012).	(2) Reluctance to meet demands and needs perceived as insatiable;<br>(3) Insecurity about how to break through the personal shield.<br>(4) Fear and difficulty in encountering existential issues. Characteristics such as empathy, compassion, courage, curiosity, and open mindedness were helpful in overcoming barriers, and the abilities to listen, to empathize, to reflect, and to switch perspective to the older person's life world seemed significant in the encounters.	as insatiable, personal shield of privacy, or fear and difficulty in encountering existential issues. Encountering existential loneliness affected the professionals and their feelings in various ways, but they generally found the experience both challenging and meaningful.
[176] Sundström et al. (2020, Sweden)	To explore existential loneliness among older people in different healthcare contexts from the perspective of healthcare professionals.	Focus group interviews were conducted with healthcare professionals (*n* = 52) in home, residential, hospital and palliative care settings. The analysis was performed in two steps: firstly, a within-case analysis of each context was conducted, followed by a cross-case analysis.<br><br>Telephone interviews conducted to describe contexts	A multiple case study design.<br><br>Inductive analytical approach conducted in two steps: a *within-case analysis* and a *cross-case analysis. T*he texts related to the following areas: (a) the professionals' views on the *origin* of existential loneliness; (b) the surroundings and the *place* where care was provided; and (c) relationships and	Existential loneliness defined as immediate awareness of being fundamentally separated from other people and from the universe, and typically, because of this awareness, experiencing negative feelings, i.e., moods and emotions.	For this review the cross case develops ideas about concepts – and the place-based context application is key. All explain EL in relation to death or disease/disability or and loss of social contact but precise meaning depends on place in which it is felt and there are complexities. So, in palliative care the home may provide for private and personal care but can also strain relationships and the historical can be a reminder one might never go home.	The context of care matters and influences how professionals view existential loneliness among older people and the opportunities they are offered to address existential loneliness. Further, existential loneliness needs to be considered in relation to the care environment and the specific care provided. The contexts' different purposes most certainly influence professionals' opportunities and

			the *role* of professionals in relation to older people's existential loneliness. The text related to the three identified themes was then re-read in depth to construct a "portrait" or story of each context.			abilities to focus on patients' existential concerns. It seems necessary to provide opportunities for structured reflection on existential matters and other phenomena relevant to caring, such as meaning, hope and consolation, based on professional's own experiences. To increase professionals' confidence in addressing existential loneliness, the support must be tailored to the specific setting and context of care. Professionals' opportunities to create trustful relationships also need to be supported by person-centred leadership focused on expanding their capacities.
[177] Kvaal et al (2014, Norway; MM)	The purposes of this paper are to focus on loneliness and social provision among older inpatients suffering from chronic illness.	To describe and compare the perceived social provision for a group reporting never feeling lonely with that of a group reporting feeling lonely and to explore the meaning of loneliness.<br><br>Participants (N = 101) were recruited from geriatric wards. The mean age was 81.3 years (range: 65–96	Mixed Method<br><br>Qualitative content analysis.	Within a phenomenological approach, the essential structure of loneliness has been identified as follows: 'loneliness is to be without the others', while there is also 'loneliness with others'. Loneliness has the potential of strength, which cannot be used because of its general	Emotions. The negative emotions expressed were thematised as 'sadness', 'anxiety and restlessness', 'anger' and 'guilt'.<br><br>Social relationships. Several expressions of the feeling of loneliness naturally included social relationships. These expressions were sorted into the themes 'being left alone', 'being confined' and 'feeling	The qualitative findings served our purpose, which was to deepen our understanding of the Loneliness among older lonely respondents' experience of loneliness. The negative emotions identified, such as the lack of meaningful and supportive social relationships, as well as the existential emptiness describe the personal consequences of feelings of emotional loneliness.

		years), 68% were women, and 66% lived alone.		negative meaning: 'loneliness is strange, wrong, ugly or even shameful'.<br><br>When the power of loneliness is given room and loneliness is voluntary, another meaning arises: 'Loneliness is restful and creative'. Loneliness can be experienced as a pleasure as well as suffering.	useless'.<br><br>Existential dimensions. The last category of the respondents' expressions contains existential dimensions of the days passing by. The words 'empty' and 'boredom' were frequently used together, with days described as being empty, slow and meaningless	Furthermore, the findings suggest that this state may inhibit trust and the initiation of new social relationships in a hospital setting.<br><br>Experience of loneliness was a state dominated by emptiness and negative emotions. The meaning content of loneliness can be mainly expressed as 'confined with emptiness and negative emotions'. The expressions indicated the respondents' emotions and evaluations of their social relationships, as well as the existential dimensions of experienced loneliness. However, some respondents also expressed loneliness as a state with potential for growth.
[178]Le Roux (1999, South Africa - GL).	To describe how the experience of loneliness unfolds in the context of the psychotherape utic dialogue. To explicate the meaning of loneliness as experienced by a patient, in the	Doctoral dissertation. N=1 long term psychotherapy patient is the subject of this study. Extensive case notes, made over 4 years, are the data source. Participant was aged 30, professional, married without children, experiencing anxiety and panic attacks.	Qualitative. Descriptive dialogic case study.<br><br>Paragraph by paragraph analysis to identify meaning units, then integrated into themes.	Loneliness is seen as an existential given. It should be differentiated from depression.<br><br>This study focuses on the disconnectedness of people to their world and to themselves.<br><br>Heidegger's existential phenomenology is one of two approaches used to	Themes relevant to conceptualisation of loneliness:<br>- Distancing of self from affective experience<br>- Feeling unprotected<br>- Inability to be alone or to tolerate silence<br>- Loss<br>- Disconnection from others	The author describes an 'unfolding' of loneliness accomplished as a part of the therapeutic relationship.<br><br>Feeling distance, unprotected, disconnected

	light of Heidegger's phenomenology and Winnicott's object relations theory.			frame discussion. In relation to this, loneliness only becomes problematic or pathological when a person cannot share their world and their experience of it with others.<br><br>Winnicott's 'object relations theory' is the other; he describes a self, struggling for individual existence, but doing so in relation to others and to the environment. For the author this is particularly important because loneliness is viewed as 'inextricably linked to one's ability to enter into relationships with others.' (p65)		
