# Supplementary material for: A Conceptual Review of Loneliness in Adults: Qualitative Evidence Synthesis
Source: Int J Environ Res Public Health. 2021 Nov 2;18(21):11522. doi: 10.3390/ijerph182111522 (PMC8582800; doi:10.3390/ijerph182111522)
Supplement: Supplementary file 1 [file ijerph-18-11522-s001.zip › SupplementaryMaterial S4.Mansfield.QualityPublished.pdf]

Supplementary Material S4 CASP Quality Assessment (Published Literature)

Authors (date)	Is the research design appropriate for addressing the aims of the research?	Was the recruitment strategy appropriate to the aims of the research?	Was the data collected in a way that addressed the research issue?	Has the relationship between researcher and participants been adequately considered?	Have ethical issues been taken into consideration?	Was the data analysis sufficiently rigorous?	Is there a clear statement of findings?	Contribution of the research to conceptualising loneliness	Total score Maximum = 8
[24]Adams (2016)	Y	Y	Y	CT	Y	Y	Y	Y	7
[25]Anaker et al (2018)	Y	Y	Y	Y	Y	Y	Y	Y	8
[26]Apostolopoulos et al (2016)	Y	CT	Y	Y	N	Y	Y	Y	6
[27]Ballin & Balandin (2009)	Y	N	N	Y	Y	Y	Y	Y	6
[28]Bantry-White et al (2018)	Y	Y	Y	CT	Y	Y	Y	Y	7
[29] Barbosa Neves, Sanders & Kokanović (2019)	Y	CT	Y	CT	Y	Y	Y	Y	6
[30]Barke J. (2017)	Y	Y	Y	Y	Y	Y	Y	Y	8
[31]Bess & Doykos (2014)	Y	N	Y	N	N	Y	Y	Y	5
[32]Bower et al (2017)	Y	Y	Y	Y	Y	Y	Y	Y	8
[33]Breck et al (2018)	Y	Y	Y	N	Y	Y	Y	Y	7
[34]Canham (2015)	Y	N	Y	CT	Y	Y	Y	N	5
[35]Cela & Fokkema (2017)	Y	Y	Y	Y	N	Y	Y	Y	7
[36]Cloutier-Fisher et al (2011)	Y	Y	Y	CT	N	Y	Y	Y	6
[37]Cohen-Mansfield & Eisner (2020)	Y	Y	Y	CT	Y	Y	Y	Y	7
[38]Cross (2011)	Y	Y	Y	CT	N	CT	Y	y	5

Authors (date)	Is the research design appropriate for addressing the aims of the research?	Was the recruitment strategy appropriate to the aims of the research?	Was the data collected in a way that addressed the research issue?	Has the relationship between researcher and participants been adequately considered?	Have ethical issues been taken into consideration?	Was the data analysis sufficiently rigorous?	Is there a clear statement of findings?	Contribution of the research to conceptualising loneliness	Total score Maximum = 8
[39]Esposito (2015)	Y	Y	Y	Y	Y	Y	Y	Y	8
[40]Fry et al (2017)	CT	CT	CT	N	N	CT	N	Y	1
[41]Goll et al (2015)	Y	Y	Y	Y	Y	Y	Y	Y	8
[42]Hauge & Kirkevold (2010)	Y	Y	Y	Y	Y	Y	Y	Y	8
[43]Heenan (2011)	Y	Y	Y	N	N	N	N	Y	4
[44]Hemingway & Jack (2013)	Y	Y	Y	Y	Y	Y	Y	Y	8
[45]Hinton & Levkoff (1999)	Y	Y	Y	CT	Y	Y	Y	Y	7
[46]Hislop et al (2015)	Y	Y	Y	CT	Y	Y	Y	Y	7
[47]Hollenbeck et al (2017)	Y	Y	Y	N	Y	Y	Y	Y	7
[48]Holtz et al (2012)	Y	Y	Y	Y	Y	Y	Y	Y	8
[49]Honigh-de Vlaming et al (2013)	Y	Y	Y	CT	CT	Y	Y	Y	6
[50]Houston et al (2016)	Y	Y	Y	Y	Y	Y	Y	Y	8
[51]Howard et al (2014)	Y	Y	Y	Y	Y	Y	Y	Y	8

Authors (date)	Is the research design appropriate for addressing the aims of the research?	Was the recruitment strategy appropriate to the aims of the research?	Was the data collected in a way that addressed the research issue?	Has the relationship between researcher and participants been adequately considered?	Have ethical issues been taken into consideration?	Was the data analysis sufficiently rigorous?	Is there a clear statement of findings?	Contribution of the research to conceptualising loneliness	Total score Maximum = 8
[52]Howard et al (2018)	Y	Y	Y	CT	Y	Y	Y	Y	7
[53]Hubach et al (2012)	Y	Y	Y	Y	Y	Y	Y	Y	8
[54]Hurtardo et al (2014)	Y	Y	Y	Y	CT	Y	Y	Y	7
[55]Janta et al (2014)	Y	Y	Y	CT	Y	CT	Y	Y	6
[56]Jerrome (1983)	Y	CT	Y	Y	CT	CT	Y	Y	5
[57]Kelchtermans et al (2011)	Y	Y	Y	CT	CT	Y	Y	Y	6
[58]Kharicha et al (2017)	Y	Y	Y	Y	Y	Y	Y	Y	8
[59]Kirkevold et al (2013)	Y	Y	Y	CT	Y	Y	Y	Y	7
[60]Korumaz (2016)	Y	Y	Y	CT	Y	Y	Y	Y	7
[61]Lanyon et al (2018)	Y	Y	Y	Y	Y	Y	Y	Y	8
[62]Lee (1994)	Y	Y	Y	Ct	Y	CT	Y	y	6
[63]Lindgren (2014)	Y	Y	Y	CT	Y	CT	Y	Y	6
[64]Lou (2012)	Y	Y	Y	CT	Y	CT	Y	Y	6

Authors (date)	Is the research design appropriate for addressing the aims of the research?	Was the recruitment strategy appropriate to the aims of the research?	Was the data collected in a way that addressed the research issue?	Has the relationship between researcher and participants been adequately considered?	Have ethical issues been taken into consideration?	Was the data analysis sufficiently rigorous?	Is there a clear statement of findings?	Contribution of the research to conceptualising loneliness	Total score Maximum = 8
[65]Mackowicz (2018)	Y	Y	Ct	n	N	CT	Y	Y	4
[66]McHugh (2017)	Y	CT	Y	N	N	CT	Y	Y	4
[67]McLaughlin (2018)	Y	Y	Y	CT	Y	Y	Y	Y	7
[68]Milson (2003)	Y	Y	Y	N	N	CT	Y	Y	5
[69] Morgan et al. (2020)	Y	Y	Y	CT	Y	Y	Y	Y	7
[70]Ojembe (2018)	Y	Y	Y	CT	Y	Y	Y	Y	7
[71]Ozawa-de Silva (2008)	CT	CT	CT	N	N	CT	Y	Y	2
[72]Paque (2018)	Y	Y	Y	CT	Y	Y	Y	Y	7
[73]Park (2017)	Y	Y	Y	CT	Y	Y	Y	Y	7
[74]Pedersen (2012)	Y	Y	Y	CT	CT	Y	Y	Y	6
[75]Pettigrew et al (2014)	Y	Y	Y	CT	Y	CT	Y	Y	6
[76]Piat et al (2018)	Y	Y	Y	CT	Y	Y	Y	Y	7

Authors (date)	Is the research design appropriate for addressing the aims of the research?	Was the recruitment strategy appropriate to the aims of the research?	Was the data collected in a way that addressed the research issue?	Has the relationship between researcher and participants been adequately considered?	Have ethical issues been taken into consideration?	Was the data analysis sufficiently rigorous?	Is there a clear statement of findings?	Contribution of the research to conceptualising loneliness	Total score Maximum = 8
[77]Pirhonen et al (2018)	Y	Y	Y	Y	Y	Y	Y	Y	8
[78]Pramuditha et al (2014)	Y	Y	Y	CT	Y	Y	Y	Y	7
[79]Rokach (1988)	Y	Y	Y	N	CT	CT	Y	Y	5
[80]Roos & Klopper (2010)	Y	Y	Y	CT	Y	CT	Y	Y	6
[81]Roos & Malan (2012)	Y	Y	Y	CT	Y	Y	Y	Y	7
[82] Roos et al (2019)	Y	Y	Y	CT	Y	Y	Y	Y	7
[83]Russell & Schofield (1999)	Y	Y	Y	CT	Y	Y	Y	N	6
[84]Sa’ar (2001)	Y	CT	CT	N	CT	Y	Y	Y	4
[85]Salas (2018)	Y	Y	Y	Y	Y	Y	Y	Y	8
[86]Schirmer et al (2015)	Y	Y	Y	N	Y	Y	Y	Y	7
[87]Smith (1998)	Y	Y	Y	N	CT	CT	Y	Y	5
[88]Smith (2012)	Y	Y	Y	CT	Y	Y	Y	y	7
[89]Stanley et al (2010)	Y	Y	Y	CT	Y	Y	Y	Y	7

Authors (date)	Is the research design appropriate for addressing the aims of the research?	Was the recruitment strategy appropriate to the aims of the research?	Was the data collected in a way that addressed the research issue?	Has the relationship between researcher and participants been adequately considered?	Have ethical issues been taken into consideration?	Was the data analysis sufficiently rigorous?	Is there a clear statement of findings?	Contribution of the research to conceptualising loneliness	Total score Maximum = 8
[90]Sullivan et al (2016)	Y	Y	Y	CT	Y	Y	Y	Y	7
[91]Taube et al (2015)	Y	Y	Y	Y	Y	Y	Y	Y	8
[92]Tiilikainen et al (2017)	Y	Y	Y	CT	Y	Y	Y	Y	7
[93]Topor et al (2016)	Y	Y	Y	CT	Y	Y	Y	CT	7
[94] Tuominen & Pirhonen (2019)	Y	Y	Y	N	Y	N	Y	Y	6
[95]Van Bergen et al (2012)	Y	Y	Y	N	CT	Y	Y	Y	6
[96]van den Berg et al (2017)	Y	Y	Y	Y	Y	Y	Y	Y	8
[97]van der Zwet et al (2009)	Y	Y	Y	N	N	Y	Y	Y	6
[98]Vasileiou et al (2017)	Y	Y	Y	N	Y	Y	Y	Y	8
[99]Vasileiou et al (2019)	Y	Y	Y	N	Y	Y	Y	Y	8
[100]Walkner et al (2018)	Y	Y	Y	N	Y	N	Y	Y	6
[101]Warren (1993)	y	CT	CT	CT	CT	y	y	y	4
[102]Winterstein & Eisikovits (2005)	Y	Y	Y	N	N	Y	Y	Y	6

Authors (date)	Is the research design appropriate for addressing the aims of the research?	Was the recruitment strategy appropriate to the aims of the research?	Was the data collected in a way that addressed the research issue?	Has the relationship between researcher and participants been adequately considered?	Have ethical issues been taken into consideration?	Was the data analysis sufficiently rigorous?	Is there a clear statement of findings?	Contribution of the research to conceptualising loneliness	Total score Maximum = 8
[103]Wong et al (2017)	Y	Y	Y	N	Y	Y	Y	Y	7
[104]Zumaeta (2019)	Y	Y	Y	Y	Y	Y	Y	Y	8
[105]Chile et al (2014)	Y	Y	Y	CT	Y	CT	Y	CT	5
[106]Dong et al (2011)	CT	Y	CT	N	N	CT	Y	Y	3
[107]Finlay et al (2018)	Y	Y	Y	CT	Y	Y	Y	Y	7
[108]Heinz, M (2018)	Y	Y	Y	N	Y	Y	Y	N	6
[109]Hinojosa et al (2011)	Y	Y	Y	CT	Y	Y	Y	Y	7
[110]Parigi et al (2014)	Y	CT	CT	CT	CT	CT	Y	Y	3
[111]Rew (2002)	Y	Y	Y	CT	Y	CT	Y	Y	6
[112]Sawir et al (2008)	Y	Y	Y	N	N	CT	Y	N	4
[113]Tahir et al (2017)	Y	Y	CT	N	Y	N	Y	N	4
[114]De Jong Gierveld et al (2018)	Y	CT	CT	CT	CT	CT	Y	Y	3
[115]Gedvilaite-Korduseine (2018)	Y	Y	Y	Y	CT	Y	Y	Y	7

Authors (date)	Is the research design appropriate for addressing the aims of the research?	Was the recruitment strategy appropriate to the aims of the research?	Was the data collected in a way that addressed the research issue?	Has the relationship between researcher and participants been adequately considered?	Have ethical issues been taken into consideration?	Was the data analysis sufficiently rigorous?	Is there a clear statement of findings?	Contribution of the research to conceptualising loneliness	Total score Maximum = 8
[116]Lake (1980)	Y	CT	Y	N	CT	Y	Y	Y	5
[117]Rook (1984)	Y	CT	CT	CT	CT	CT	Y	Y	3
[132]Bates & Machin (2015)	Y	Y	Y	N	Y	Y	Y	Y	7
[133]Bennett & Victor (2012)	Y	N/A	Y	N/A	Y	Y	Y	Y	6
[134]Costello (1999)	Y	Y	Y	CT	N	CT	N	Y	5
[135]Davies et al (2016)	Y	Y	Y	Y	Y	Y	Y	Y	8
[136] Florczak & Lockie (2018)	Y	Y	Y	Y	Y	Y	Y	Y	4
[137]Graneheim & Lundman (2010)	Y	Y	Y	Y	Y	CT	Y	N	6
[138]Heravi-Karimooi (2010)	Y	CT	Y	CT	Y	Y	Y	Y	6
[139]Karlsson et al (2013)	Y	Y	Y	Y	Y	Y	Y	Y	8
[140]Mc Innes (2001)	Y	Y	Y	N	N	Y	Y	Y	6
[141]Muir & McGrath (2018)	Y	Y	Y	Y	Y	Y	Y	Y	8
[142]Nunkoosing (2013)	Y	CT	CT	CT	CT	CT	CT	Y	1

Authors (date)	Is the research design appropriate for addressing the aims of the research?	Was the recruitment strategy appropriate to the aims of the research?	Was the data collected in a way that addressed the research issue?	Has the relationship between researcher and participants been adequately considered?	Have ethical issues been taken into consideration?	Was the data analysis sufficiently rigorous?	Is there a clear statement of findings?	Contribution of the research to conceptualising loneliness	Total score Maximum = 8
[143]Pettigrew et al (2008)	Y	Y	Y	CT	CT	Y	Y	Y	6
[144]Riches (1996)	Y	CT	Y	Y	Y	Y	Y	Y	7
[145]Rokach (1989)	Y	Y	Y	N	CT	CT	Y	Y	5
[146]Sagan (2008)	Y	Y	Y	Y	Y	Y	Y	Y	8
[147]Theeke et al (2015)	Y	Y	Y	Y	Y	Y	Y	Y	8
[148] Wijesiri et al (2019)	Y	Y	Y	N	Y	Y	Y	Y	7
[149]Wiseman (2008)	Y	Y	Y	N	N	Y	Y	Y	6
[150]Barg et al (2006)	Y	Y	Y	Y	Y	CT	Y	Y	7
[151]Drageset et al (2015)	Y	Y	Y	Y	Y	Y	Y	Y	8
[152]Marcille et al (2012)	Y	Y	Y	N	N	Y	Y	N	5
[153]Merz & Gierveld (2016)	Y	CT	Y	N	Y	Y	Y	Y	6
[154]Corcoran & Marshall (2018)	CT	CT	N	CT	CT	CT	Y	Y	2
[155]de Jong Gierveld et al (2016)	Y	CT	CT	CT	CT	CT	Y	Y	3

Authors (date)	Is the research design appropriate for addressing the aims of the research?	Was the recruitment strategy appropriate to the aims of the research?	Was the data collected in a way that addressed the research issue?	Has the relationship between researcher and participants been adequately considered?	Have ethical issues been taken into consideration?	Was the data analysis sufficiently rigorous?	Is there a clear statement of findings?	Contribution of the research to conceptualising loneliness	Total score Maximum = 8
[159]Cherry & Smith (1993)	Y	Y	Y	N	Y	Y	Y	Y	7
[160]Chung et al (2020)	Y	Y	Y	Y	Y	Y	Y	Y	8
[161]Dahlberg (2007)	N	N	CT	N	N	CT	N	Y	1
[162]Goldberg (2001)	Y	Y	Y	y	N	CT	Y	Y	6
[163]Goosens et al (2015)	Y	Y	Y	CT	N	Y	Y	Y	6
[164]Hemberg et al (2018)	Y	Y	Y	N	Y	Y	Y	Y	7
[165]Larsson (2017)	Y	Y	Y	CT	Y	Y	Y	Y	7
[166]Larsson et al (2019)	Y	Y	Y	Y	Y	Y	Y	Y	8
[167]Nillson (2007)	Y	Y	CT	CT	CT	CT	Y	Y	4
[168]Nortvedt (2015)	Y	Y	Y	Y	Y	CT	Y	Y	7
[169]Nystrom (2006)	Y	CT	Y	CT	Y	Y	Y	Y	6
[170]Rosedale (2009)	Y	Y	Y	CT	CT	Y	Y	Y	6
[171]Sagan (2017)	Y	Y	Y	Y	Y	Y	Y	Y	8

Authors (date)	Is the research design appropriate for addressing the aims of the research?	Was the recruitment strategy appropriate to the aims of the research?	Was the data collected in a way that addressed the research issue?	Has the relationship between researcher and participants been adequately considered?	Have ethical issues been taken into consideration?	Was the data analysis sufficiently rigorous?	Is there a clear statement of findings?	Contribution of the research to conceptualising loneliness	Total score Maximum = 8
[172]Sand et al (2006)	Y	Y	Y	CT	Y	Y	Y	Y	7
[173]Sjöberg et al (2017)	Y	Y	Y	Y	Y	Y	Y	Y	8
[174]Stein et al (2014)	Y	Y	Y	Y	Y	Y	Y	Y	8
[175]Sundstrom et al (2018)	Y	Y	Y	Y	Y	Y	Y	Y	8
[176] Sundström et al (2020)	Y	Y	Y	CT	Y	Y	Y	Y	7
[177]Kvaal et al (2014)	Y	Y	Y	Y	Y	Y	Y	Y	8
