# Supplementary material for: A Conceptual Review of Loneliness in Adults: Qualitative Evidence Synthesis
Source: Int J Environ Res Public Health. 2021 Nov 2;18(21):11522. doi: 10.3390/ijerph182111522 (PMC8582800; doi:10.3390/ijerph182111522)
Supplement: Supplementary file 1 [file ijerph-18-11522-s001.zip › SupplementaryMaterial S5.MansfieldQualityGL.pdf]

Supplementary Material S5: Quality Assessment (Grey Literature)

Author surname & date, type of output	Credibility Rating	Reasons
[118]Batsleer et al (2018) Evaluation Report	Moderate	Description of methods. Non-theoretical detail reported although evidence that it formed the framework for coproduction. Self-reflective section considering the view from the co-researchers.
[119]Brown (2019)	Moderate	Detailed description of the data collection, analysis and a thorough examination of the themes from the qualitative data. Limitations are also considered, but there is a lack of theoretical underpinnings to the research.
[120]Essex (2010): PhD Thesis	High	Highly detailed methods and analysis. Small sample, but strong data and theoretically informed narrative. Limitations considered.
[121]Huijbers (2017) Evaluation Report	Low	Description of methods. Relies on face value reporting of participants' accounts. Identifies themes in its analysis but not informed by theory.
[122]Lukes-Dyer (2018) PhD Thesis	High	Detailed description and reflection on methods, approach and limitations to the qualitative study. Analysis informed by identified theories.
[123] Macomber (2017) PhD Thesis	High	Detailed description and theoretical reflection on methods, approach, and limitations. Attention to assessment of quality for the qualitative elements. Analysis is theoretically informed
[124]Moore & Preston (2015) Evaluation Report	Moderate	Descriptive detail about the choice and use of evaluation methods. Little detail on participants and little discussion of potential limitations.
[125]Mental Health Foundation (2018) Evaluation Report	High	Descriptive and theoretical detail about methods and analysis. There is some useful reflection on evaluation limitations and recommendations for future similar studies.
[126]Qin (2017) PhD Thesis	Low	Very brief descriptive and theoretical detail about methods and analysis. While there is discussion of the limitations of the project in general, there is little discussion of the qualitative element specifically.
[127]Quinn and Blandon (2014) Evaluation Report	Moderate	Describes methods and analysis. Identifies the limitations. Lacks analytical and theoretically informed detail.
[128]Sital-Singh et al (2018) Evaluation Report	Low	No analysis reported, methods brief and descriptive, limitations not considered.
[129]Todd (2017) PhD Thesis	High	Comprehensive description and analysis underpinned by theoretical detail. Limitations noted.
[120]Zubairi (2018) Evaluation Report	Moderate	Methods identified but lacks details. No discussion of limitations, no detail about analysis methods, and no detailed information about participants.
[131] British Red Cross (2016)	Moderate	Description and consideration of methods, approach and analysis. No reflection on methodological limitations. Informed by subject appropriate theory. Comprehensive data.

[157]Haines (2018) Evaluation Report	Moderate	Descriptive detail on methods and for analysis of data. Indication of attempts to improve data reliability and avoid bias. Presentation of identified themes and analysis is not underpinned by theory. Little discussion of limitations.
[158]Hall (2012):	High	Thorough description and consideration of methods, approach and limitations. Small sample, but study fully informed by identified theory.
[159]Henrich (2019)	High	Highly detailed description of data collection methods and data analysis, with a strong theoretically informed approach to the analysis. Limitations are also considered.
[179]Le Roux (2001) PhD Thesis	Moderate	Detailed description and reflection on methods, approach and limitations to the study, all informed by identified theory. Its single individual case study approach might be considered a limitation. Longitudinal design (following the same individual over a 4 year period) is a strength.
