# Supplementary material for: A Conceptual Review of Loneliness in Adults: Qualitative Evidence Synthesis
Source: Int J Environ Res Public Health. 2021 Nov 2;18(21):11522. doi: 10.3390/ijerph182111522 (PMC8582800; doi:10.3390/ijerph182111522)
Supplement: Supplementary file 1 [file ijerph-18-11522-s001.zip › SupplementaryMaterial S6.Mansfield.CerQual.pdf]

Appendix S6: CERQual Qualitative Evidence Profile

Review findings	Studies contributing to the review finding	Methodological limitations (ML) component	Relevance component	Coherence component	Adequacy of data component	Overall CERQual assessment of confidence	Explanation of judgement
Conceptualising and understanding social loneliness (n=108)	24-131	Minor concerns for ML (13 published studies several ML, 61 minor ML, 20 studies maximum quality). (5 grey literature high, 6 moderate, 3 low)	Minor concerns for relevance (all studies examined social loneliness)	Minor concerns for coherence (data reasonably consistent within studies, low consistency across studies on population and context)	Minor concerns about adequacy (13 published studies thin data, 81 moderate richness of data). (5 grey literature high, 6 moderate and 3 low)	**High Confidence**	Graded as high confidence due to minor concerns with methodological limits, coherence and adequacy
Conceptualising and understanding emotional loneliness (n=27)	132-158	Moderate concerns for ML (4 published studies several limitations, 14 minor ML, 6 max. quality). (2 grey literature moderate and 1 high)	Minor concerns for relevance (all studies examined emotional loneliness)	Moderate concerns for coherence (data reasonably consistent within studies, low consistency across studies population/context)	Moderate concerns about adequacy (4 published studies thin data, 20 moderate richness of data.) (1 grey literature moderate,2 high)	**Moderate Confidence**	Graded as moderate confidence due to moderate concerns with methodological limits, coherence and adequacy
Conceptualising and understanding existential loneliness (n=20)	159-178	Moderate concerns for ML (2 published studies several ML, 10 minor ML, 7 max. quality). (1 grey literature moderate)	Minor concerns for relevance (all studies examined existential loneliness)	Moderate concerns for coherence (data reasonably consistent within studies, low consistency across studies on population and context)	Moderate concerns about adequacy (2 published studies thin data, 17 moderate richness of data). (1 grey literature moderate)	**Moderate Confidence**	Graded as moderate confidence due to moderate concerns with methodological limits, coherence and adequacy
